# Extending a Gray Lattice Boltzmann Model for Simulating Fluid Flow in Multi-Scale Porous Media

**DOI:** 10.1038/s41598-018-24151-2

**Published:** 2018-04-11

**Authors:** Jiujiang Zhu, Jingsheng Ma

**Affiliations:** 1 School of Civil Engineering, Wuyi University, Jiangmen, Gunagdong Province, China; 2 Institute of Petroleum Engineering, Heriot-Watt University, Edinburgh, UK

## Abstract

A gray lattice Boltzmann model has previously been developed by the authors of this article to simulate fluid flow in porous media that contain both resolved pores and grains as well as aggregates of unresolved smaller pores and grains. In this model, a single parameter is introduced to prescribe the amount of fluid to be bounced back at each aggregate cell. This model has been shown to recover Darcy-Brinkman flow but with effective viscosity and permeability correlated through the model parameter. In this paper, we prove that the model parameter relates to the fraction of the solid phase of a sub-pore system for a specific set of bounce-back conditions. We introduce an additional parameter to the model, and this enables flow simulation in which cases with variable effective viscosity and permeability can be specified by selecting the two parameters independently. We verify and validate the model for layered channel cases and mathematically analyze fluid momentum and energy losses for the single- and two-parameter models to explain the roles of the parameters in their conservation. We introduce a strategy to upgrade our model to an isotropic version. We discuss the fundamental differences between our model and the Brinkman body-force LBM scheme.

## Introduction

The pore systems of natural porous media, such as rocks and soils, are multi-scaled, containing pores and grains of variable sizes. Since not all pores and grains can be fully resolved in a grid-based model, grid cells need to represent resolved and unresolved pores and grains simultaneously. For each cell that contains an aggregate of unresolved pores and grains, the subscale effect of that aggregate on the fluid flow needs to be accounted for. In our previous work^1^, we reviewed several gray lattice Boltzmann models (GLBMs)^2–5^ of a BGK^6^ lattice type that have been developed for this purpose and proposed an improved single-parameter model. In this model, the parameter, denoted as *n*_*s*_, prescribes the amount of fluid particle to be bounced back at each cell. This model has been shown to recover Darcy-Brinkman flow and to possess better properties than its counterpart models in terms of the physical interpretability of the parameter as well as numerical accuracy and stability.

For convenience of the discussion to follow, we recap key steps of this model as follows.

Let *f*(**ξ**, **r**, *t*) denote the particle distribution function (PDF), meaning the probability of finding a fluid particle with a velocity **ξ** at location **r** at time *t*. Then, the Boltzmann equation reads

1 $$\frac{\partial f({\boldsymbol{\xi }},{\bf{r}},t)}{\partial t}+{\boldsymbol{\xi }}\cdot \frac{\partial f({\boldsymbol{\xi }},{\bf{r}},t)}{\partial {\bf{r}}}+\frac{1}{\rho }{\bf{F}}\cdot \frac{\partial f({\boldsymbol{\xi }},{\bf{r}},t)}{\partial {\boldsymbol{\xi }}}=-\,\frac{f-{f}^{eq}}{\tau }$$

where *ρ* is the mass density of the fluid, **F** is a body force applied per unit volume, *τ* is the relaxation time, and *f*^* eq*^ is the equilibrium PDF. On a standard lattice of a DdQb stencil with directions **e**_*α*_(*α* = 1, 2, …, *b*), assume that the mass of each particle is *m*, the length of the space lattice is Δ*x*, the time step is Δ*t*, and the velocity **ξ**_*α*_ = Δ*x***e**_*α*_. Then, define the following discrete variables:

2 $${f}_{\alpha }={f}_{\alpha }({\bf{r}},t)\equiv mf({{\boldsymbol{\xi }}}_{\alpha },{\bf{r}},t)$$

Then, our previous GLBM is defined for dimensionless quantities of the dimensional counterparts above (see Table 1 and related explanations) and consists of the following steps:

**Table 1:** Definitions of dimensionless variables of GLBM.

*c* = $\frac{{\rm{\Delta }}x}{{\rm{\Delta }}t}$	$\tilde{\tau }=\frac{\tau }{{\rm{\Delta }}t}$	${\tilde{\tau }}^{\ast }=\tilde{\tau }+\frac{1}{2}$	$\tilde{{\bf{r}}}=\frac{{\bf{r}}}{{\rm{\Delta }}x}$
$\tilde{{\bf{u}}}=\frac{{\bf{u}}}{c}$	$\tilde{\theta }=\frac{\theta }{{c}^{2}}\equiv \frac{1}{3}$	$\tilde{\rho }=\frac{\rho }{{\rho }_{c}}$	${\tilde{f}}_{\alpha }=\frac{{f}_{\alpha }}{{\rho }_{c}}$
${\tilde{f}}_{\alpha }^{eq}=\frac{{f}_{\alpha }^{eq}}{{\rho }_{c}}$	$\tilde{{\bf{F}}}=\frac{{\bf{F}}{\rm{\Delta }}t}{{\rho }_{c}c}$	*ρc* is reference mass density	

Collision step:

3 $${\tilde{f}}_{\alpha }^{c}(\tilde{{\bf{r}}},{\tilde{t}}^{\ast })={\tilde{f}}_{\alpha }(\tilde{{\bf{r}}},\tilde{t})-\frac{{\tilde{f}}_{\alpha }(\tilde{{\bf{r}}},\tilde{t})-{\tilde{f}}_{\alpha }^{eq}(\tilde{{\bf{r}}},\tilde{t})}{{\tilde{\tau }}^{\ast }}+\frac{\tilde{{\bf{F}}}\cdot ({{\bf{e}}}_{\alpha }-\tilde{{\bf{u}}})}{\tilde{\theta }}{{\rm{\Gamma }}}_{\alpha }(\tilde{{\bf{u}}})$$

Repartitioning step:

4 $${\tilde{f}}_{\alpha }^{out}(\tilde{{\bf{r}}},{\tilde{t}}^{\ast \ast })=(1-{n}_{s}){\tilde{f}}_{\alpha }^{c}(\tilde{{\bf{r}}},{\tilde{t}}^{\ast })+{n}_{s}{\tilde{f}}_{\bar{\alpha }}^{c}(\tilde{{\bf{r}}},{\tilde{t}}^{\ast }),\alpha \ne 0$$

Streaming step:

5 $${\tilde{f}}_{\alpha }(\tilde{{\bf{r}}}+{{\bf{e}}}_{\alpha },\tilde{t}+1)={\tilde{f}}_{\alpha }^{out}(\tilde{{\bf{r}}},{\tilde{t}}^{\ast \ast }),\alpha \ne 0$$

Particularly for a D2Q9 lattice stencil, the equilibrium distribution takes the following form:

6 $${\tilde{f}}_{\alpha }^{eq}(\tilde{{\bf{r}}},\tilde{t})=\tilde{\rho }{{\rm{\Gamma }}}_{\alpha }(\tilde{{\bf{u}}})=\tilde{\rho }{w}_{\alpha }[1+\frac{({{\bf{e}}}_{\alpha }\cdot \tilde{{\bf{u}}})}{\tilde{\theta }}+\frac{{({{\bf{e}}}_{\alpha }\cdot \tilde{{\bf{u}}})}^{2}}{2{\tilde{\theta }}^{2}}-\frac{{\tilde{{\bf{u}}}}^{2}}{2\tilde{\theta }}]$$

where **e**_*α*_ and *w*_*α*_ can be found in the literature^1^.

In the reference paper^1^ and this paper, we use $\bar{\alpha }$ to represent the direction opposite to *α* and “~” on the top of a variable to denote its dimensionless counterpart. Table 1 shows the definitions of the dimensionless variables used in the above equations. For any other dimensionless variable that appears in this article but not in the table, an implicit dimensionless-scaling rule is applied with respect to the corresponding base, which takes either Δ*t*, Δ*x* or *ρ*_*c*_. For example, the dimensionless channel width can be defined as $\tilde{h}=\frac{H}{{\rm{\Delta }}x}$, where *H* is the physical width of a channel.

In Eqs (3) and (6), $\tilde{t}$, ${\tilde{t}}^{\ast }$ and ${\tilde{t}}^{\ast \ast }$ signify the cyclic transition of the operations, from prior-collision to collision, repartition, and streaming.

In our previous GLBM, there is a repartitioning step that accomplishes partial bouncing back as specified by ${n}_{s}$. This model has been proved to recover the Darcy-Brinkman flow defined in Eq. (7)

7 $${v}_{eff}{\nabla }^{2}\tilde{{\bf{u}}}-\frac{{v}_{f}}{{k}_{eff}}\tilde{{\bf{u}}}+\frac{\tilde{{\bf{F}}}}{\tilde{\rho }}=0$$

where *ν*_*eff*_ and *κ*_*eff*_ are the effective viscosity and effective permeability, respectively, and *ν*_*f*_ is the viscosity of fluid

8 $${\nu }_{f}=\frac{\nu }{{\rm{\Delta }}t{c}^{2}}=\frac{1}{3}({\tilde{\tau }}^{\ast }-\frac{1}{2})$$

By analyzing our GLBM^1^ and one of the counterpart models^5^, we show mathematically that the relationship ${n}_{s}=\frac{{\lambda }_{s}}{1+{\lambda }_{s}}$ must hold, where *λ*_s_ is the model parameter of the counterpart GLBM. n_*s*_ and *λ*_s_ are valid for 0 ≤ *n*_*s*_ ≤ 0.5 and 0 ≤ *λ*_s_ ≤ 1, respectively. *λ*_s_ is regarded by those authors^5^ as the local fraction of the solid phase. They acknowledged that, in reality, *λ*_s_ alone cannot determine how much fluid would be bounced back or repartitioned by the solid phase. At present, there is no proof of the existence of reasonable fluid and solid interaction conditions for this relationship to hold. This implies that even if the details of the local distribution of a pore structure are known, one would not know how to estimate *n*_*s*_ at each cell.

The second limitation is that our single-parameter GLBM does not recover independent *ν*_*eff*_ and *κ*_*eff*_. For a channel model filled with a homogenous and isotropic porous material, we show that *ν*_*eff*_ and *κ*_*eff*_ are related through *n*_*s*_ for any given fluid whose viscosity must be prescribed by choosing an appropriate ${\tilde{\tau }}^{\ast }$. Therefore, this single-parameter GLBM is inflexible and incapable of simulating fluid flow in porous media with independent *ν*_*eff*_ and *κ*_*eff*_.

This paper aims to show how these two limitations can be overcome. We first prove that ${n}_{s}=\frac{{\lambda }_{s}}{1+{\lambda }_{s}}$ holds for a set of partial bounce-back conditions. Then, we introduce an additional parameter, $\eta$, to define a fraction of post-partition particles that are subject to streaming. We derive mathematic formulae for *ν*_*eff*_ and *κ*_*eff*_ with respect to *n*_*s*_, $\eta$ and ${\tilde{\tau }}^{\ast }$. For a channel model filled with a homogenous and isotropic porous material, we show that this two-parameter model allows one to adjust the two parameters independently to simulate the Darcy-Brinkman flow with realistic effective viscosity and permeability. We also derive mathematic formulae for fluid momentum and energy losses for our single- and two-parameter models to explain the roles of the parameters in their conservation.

In addition to validating our model numerically for layered channel cases, we examine the model accuracy and applicability with respect to the lattice orientation alignment relative to the channel direction. For the Brinkman body-force (BF) LBM scheme^7,8^, it is already known that the resultant Darcy-Brinkman flow has an angular-dependent effective viscosity coefficient^9^. We show that our model can be adopted further to eliminate this anisotropic effect.

The remainder of the paper is organized as follows. Section 2 proves the existence of a set of conditions for ${n}_{s}=\frac{{\lambda }_{s}}{1+{\lambda }_{s}}$ to hold. Section 3 introduces a two-parameter model and derives and examines the relationships of *ν*_*eff*_ and *κ*_*eff*_ with respect to *n*_*s*_, *η* and ${\tilde{\tau }}^{\ast }$. Section 4 presents numerical experiments for three cases. Section 5 examines the accuracy and applicability of the model on lattices whose cardinal directions are at 0° and 45° to the channel direction with respect to analytic solutions for a number of cases. As reported in^9^, there is a discrepancy between the numerical simulation of a BF scheme and its macroscopic equation recovered by Chapman Enskog expansion; even if the macroscopic parameter was kept as a constant, a different relaxation time resulted in a different velocity profile. In Section 5.3, we demonstrate that the GLBM proposed in this paper is consistent with the macroscopic equation recovered by this model. As long as the macroscopic parameters were kept constant, different model parameters exactly predicted the same velocity profile. In Section 5.4, we introduce a strategy to upgrade our model to recover isotropic flow on a non-channel-aligned lattice. This is then followed by a discussion (Section 6) in which the differences between our model and a body-forced LBM are revealed. The final section (Section 7) offers our conclusion.

## Link between the fraction of fluid particles to be bounced back and the fraction of the solid phase

In our previous model^1^, *n*_*s*_ was introduced as the sole model parameter to capture the effect of subscale porous media on fluid flow; its physical meaning is clearly defined as the fraction of fluid particles to be bounced back by the solid phase. This parameter needs to be estimated in order to use the model. In another single-parameter model^5^, *λ*_*s*_ was introduced as the model parameter, and it was interpreted by those authors as the fraction of the solid phase. In our work^1^, we proved that *n*_*s*_ and *λ*_*s*_ are related via ${n}_{s}=\frac{{\lambda }_{s}}{1+{\lambda }_{s}}$ for any porous model with a homogenous and isotropic pore structure^1^. However, because *λ*_*s*_ alone cannot determine how much fluid would be bounced back or repartitioned by the solid phase without considering the structures of the pores, it is not clear under what conditions that relationship can be used to derive a reasonable estimate of *n*_*s*_.

For a homogenous and isotropic porous material, we show below that the relationship above holds for ${n}_{s}$ and *λ*_*s*_ under the following assumptions: (1) the fraction of fluid particles to be bounced back is a constant and equal to the volume fraction of the solid phase, and (2) the fluid particles that have already been bounced back may be repeatedly bounced back. Note that inside the local porous medium, the fluid particles are subject to repeated bouncing back such that the total number of fluid particles in an LB direction is the summation of all fluid particles that come out from that direction.

To prove this, let *l*_*i*_ denote the fraction of fluid particles that are valid for further bouncing back after the first *i* times of bouncing back; let *s*_*i*_ and *b*_*i*_ denote the fraction of accumulated fluid particles that go forward and backward, respectively. Based on the assumptions above, we have ${b}_{1}={\lambda }_{s}$, ${s}_{1}=1-{\lambda }_{s}$ and ${l}_{1}={\lambda }_{s}$ at *i* = 1. At *i* = 2, the fraction of fluid particles that have been bounced back, i.e., *l*_1_, will be bounced back again, and therefore, we have ${b}_{2}={b}_{1}-{\lambda }_{s}^{2}$, ${s}_{2}={s}_{1}+{\lambda }_{s}^{2}$ and ${l}_{2}={\lambda }_{s}^{2}$. In general, at $i=n$, we have ${b}_{n}={b}_{n-1}+{(-1)}^{n+1}{\lambda }_{s}^{n}$, ${s}_{n}={s}_{n-1}+{(-1)}^{n}{\lambda }_{s}^{n}$ and ${l}_{n}=\lambda {l}_{n-1}={\lambda }_{s}^{n}$. By straightforward manipulations, we can derive the following relationship for *n*_*s*_and *λ*_*s*_:

9 $${n}_{s}={\lambda }_{s}(1-{\lambda }_{s})[1+{\lambda }_{s}^{2}+{\lambda }_{s}^{4}+\cdots ]=\frac{{\lambda }_{s}}{1+{\lambda }_{s}}$$

Therefore, based on our previous results and Eq. (9), we can determine *n*_*s*_ from the fraction of the solid phase as a reasonable estimate for a local homogenous and isotropic porous medium. This is important for the proposed application^10^.

## A two-parameter GLBM

For any single-parameter GLBM, it is difficult to capture the full flow behaviors characterized by both the permeability and effective viscosity^11^. Here, we introduce a two-parameter GLBM with an additional parameter to our single-parameter model to overcome this limitation. In the single-parameter model, recall that the post-collision fluid particles in each direction are divided into two proportions, defined by *n*_*s*_ and 1 − *n*_*s*_; the former defines the amount to be bounced back, whereas the latter defines the amount to be allowed to pass through. Then, we define the extra parameter, $\eta$, to be the amount of post-partition particles for streaming. The precise role of $\eta$ is explained as follows.

In our single-parameter model, $${\tilde{f}}_{\alpha }(\tilde{{\bf{r}}},\tilde{t})$$ stands for the probability distribution function in the $$\alpha $$ direction. The post-collision fluid particles $${\tilde{f}}_{\alpha }^{c}(\tilde{{\bf{r}}},{\tilde{t}}^{\ast })(\alpha \ne 0)$$ are divided into two parts: $$(1-{n}_{s}){\tilde{f}}_{\alpha }^{c}(\tilde{{\bf{r}}},{\tilde{t}}^{\ast })$$ and $${n}_{s}{\tilde{f}}_{\alpha }^{c}(\tilde{{\bf{r}}},{\tilde{t}}^{\ast })$$, where the former is streamed along $$\alpha $$, whereas the latter is bounced backward along $$\bar{\alpha }$$, the direction opposite to $$\alpha $$. In the two-parameter model, each of the two parts is further divided into two parts: $$\eta (1-{n}_{s}){\tilde{f}}_{\alpha }^{c}(\tilde{{\bf{r}}},{\tilde{t}}^{\ast })$$ and $$(1-\eta )$$$$(1-{n}_{s}){\tilde{f}}_{\alpha }^{c}(\tilde{{\bf{r}}},{\tilde{t}}^{\ast })$$, as well as $$\eta {n}_{s}{\tilde{f}}_{\alpha }^{c}(\tilde{{\bf{r}}},{\tilde{t}}^{\ast })$$ and $$(1-\eta ){n}_{s}{\tilde{f}}_{\alpha }^{c}(\tilde{{\bf{r}}},{\tilde{t}}^{\ast })$$; the fluid particles associated with $$\eta $$ are allowed to be streamed, whereas the fluid particles associated with $$(1-\eta )$$ are added to $${\tilde{f}}_{0}(\tilde{{\bf{r}}},{\tilde{t}}^{\ast })$$, the null-direction PDF component, i.e., $$\alpha =0$$, with an increment of $$(1-\eta )\sum _{\alpha \ne 0\,}{\tilde{f}}_{\alpha }^{c}=(1-\eta )(\tilde{\rho }-{\tilde{f}}_{0}^{c})$$. Therefore, the two-parameter model has a new repartitioning step defined in Eq. (10) below.

Repartitioning:

10 $${\tilde{f}}_{\alpha }^{out}(\tilde{{\bf{r}}},{\tilde{t}}^{\ast \ast })=\{\begin{array}{ll}\eta [(1-{n}_{s}){\tilde{f}}_{\alpha }^{c}(\tilde{{\bf{r}}},{\tilde{t}}^{\ast })+{n}_{s}\,{\tilde{f}}_{\bar{\alpha }}^{c}(\tilde{{\bf{r}}},{\tilde{t}}^{\ast })] & {\rm{if}}\,\alpha \ne 0\\ (1-\eta )\tilde{\rho }+\eta \,{\tilde{f}}_{0}^{c} & {\rm{if}}\,\alpha =0\end{array}$$

Following the same procedure^1^, we can prove that the two-parameter model recovers the Darcy–Brinkman equation in the finite difference form as follows:

11 $$A({\tilde{u}}_{x}^{j-1}+{\tilde{u}}_{x}^{j+1}-2{\tilde{u}}_{x}^{j})-B{\tilde{u}}_{x}^{j}+C\frac{{\tilde{F}}_{x}}{\tilde{\rho }}=0$$

where

12 $$\{\begin{array}{c}A=(1-{\rm{2}}\gamma )(1-{n}_{s})+\frac{(1-2{n}_{s})}{6({\tilde{\tau }}^{\ast }-1)}\\ B=\omega (1-{\rm{2}}\gamma )+\frac{(1-2{n}_{s})}{3}(\frac{\eta }{{\tilde{\tau }}^{\ast }}-\frac{1}{{\tilde{\tau }}^{\ast }-1})\\ C=[{\rm{2}}\gamma \omega -\frac{(1-2{n}_{s})}{3}(\frac{\eta }{{\tilde{\tau }}^{\ast }}-\frac{1}{{\tilde{\tau }}^{\ast }-1})]{\tilde{\tau }}^{\ast }\end{array}$$

13 $$\{\begin{array}{c}\omega =\frac{{\tilde{\tau }}^{\ast }}{\eta ({\tilde{\tau }}^{\ast }-1)}+\eta (1-\frac{1}{{\tilde{\tau }}^{\ast }})(1-2{n}_{s})-2(1-{n}_{s})\\ \gamma =\frac{(1-2{n}_{s})\eta }{{\rm{3}}[{\tilde{\tau }}^{\ast }-\eta ({\tilde{\tau }}^{\ast }-1)(1-2{n}_{s})]}\end{array}$$

When Eq. (11) converges to the Darcy–Brinkman equation, we prove that the effective viscosity and the effective permeability are given by Eqs (14) and (15), respectively. For a complete derivation, the reader is referred to Appendix A.

14 $${v}_{eff}=\frac{A}{C}=\frac{{\tilde{\tau }}^{\ast }[6{\tilde{\tau }}^{\ast }-5-{n}_{s}(6{\tilde{\tau }}^{\ast }-4)]+(1-2{n}_{s})\eta ({\tilde{\tau }}^{\ast }-1)[1+6({n}_{s}-1){\tilde{\tau }}^{\ast }]}{6[{\tilde{\tau }}^{\ast }(1-2{n}_{s})-(1-2{n}_{s})\eta ({\tilde{\tau }}^{\ast }-1)][{\tilde{\tau }}^{\ast }-(1-2{n}_{s})\eta ({\tilde{\tau }}^{\ast }-1)]}$$

15 $${k}_{eff}=\frac{C}{B}{\nu }_{f}=\frac{(1-2{n}_{s})\eta }{1-(1-2{n}_{s})\eta }\frac{(2{\tilde{\tau }}^{\ast }-1)}{6}$$

Using Eqs (14) and (15) and (11) can be rewritten as

16 $${v}_{eff}({\tilde{u}}_{x}^{j-1}+{\tilde{u}}_{x}^{j+1}-2{\tilde{u}}_{x}^{j})-\frac{{\nu }_{f}}{{k}_{eff}}{\tilde{u}}_{x}^{j}+\frac{{\tilde{F}}_{x}}{\tilde{\rho }}=0$$

## Numerical experiments

**Figure 1:**
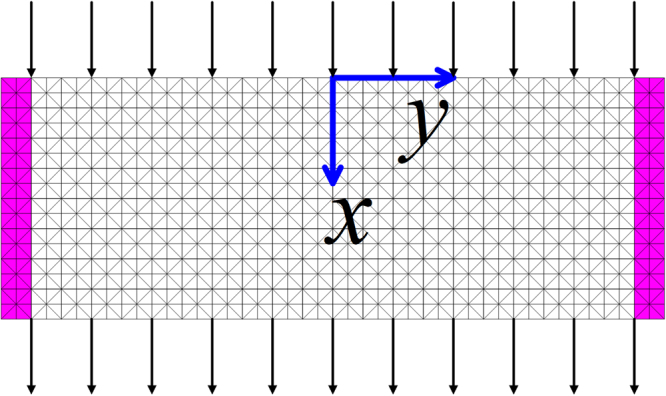
Model domain and flow configuration.

In this section, we perform three numerical experiments on a horizontal channel filled with a homogeneous and isotropic porous material, as shown in Figure 1. A D2Q9 implementation of the two-parameter model is used to simulate the fluid flow driven by a constant horizontal body force $({\tilde{F}}_{x},\,0)=(0.0001\times \tilde{\rho },\,0)$ from zero velocity until it becomes steady. The channel is discretized into 101 nodes in the vertical direction and 20 nodes in the horizontal direction. A periodical boundary condition is applied in the $\mathop{x}\limits^{ \sim }-$direction, while solid wall boundaries at the left and right columns of the nodes are prescribed with the non-slip boundary condition^12^. The relaxation time is chosen to be ${\tilde{\tau }}^{\ast }=2$.

We investigate the vertical profiles of the terminal velocity at the midpoint on the $x$-axis for three cases. In each case, we calculate the vertical profiles for two different media. Table 2 lists the parameters $({n}_{s},\eta )$ for media A and B and calculates ${\kappa }_{eff}$ and ${\nu }_{eff}$ using Eqs (14) and (15), respectively.

**Table 2:** Input parameters and calculated quantities for the three cases.

Case	Medium	${{\boldsymbol{n}}}_{{\boldsymbol{s}}}$	${\boldsymbol{\eta }}$	${{\boldsymbol{\kappa }}}_{{\boldsymbol{e}}{\boldsymbol{f}}{\boldsymbol{f}}}$	${{\boldsymbol{\nu }}}_{{\boldsymbol{e}}{\boldsymbol{f}}{\boldsymbol{f}}}$
1	A	0.3333	0.3000	0.0556	1.2330
	B	0.3333	0.8000	0.1819	1.6344
2	A	0.4444	0.8000	0.0488	4.1733
	B	0.0909	0.1087	0.0488	0.6574
3	A	0.2500	0.4000	0.1250	0.9722
	B	0.3750	0.8000	0.1250	2.0679

**Figure 2:**
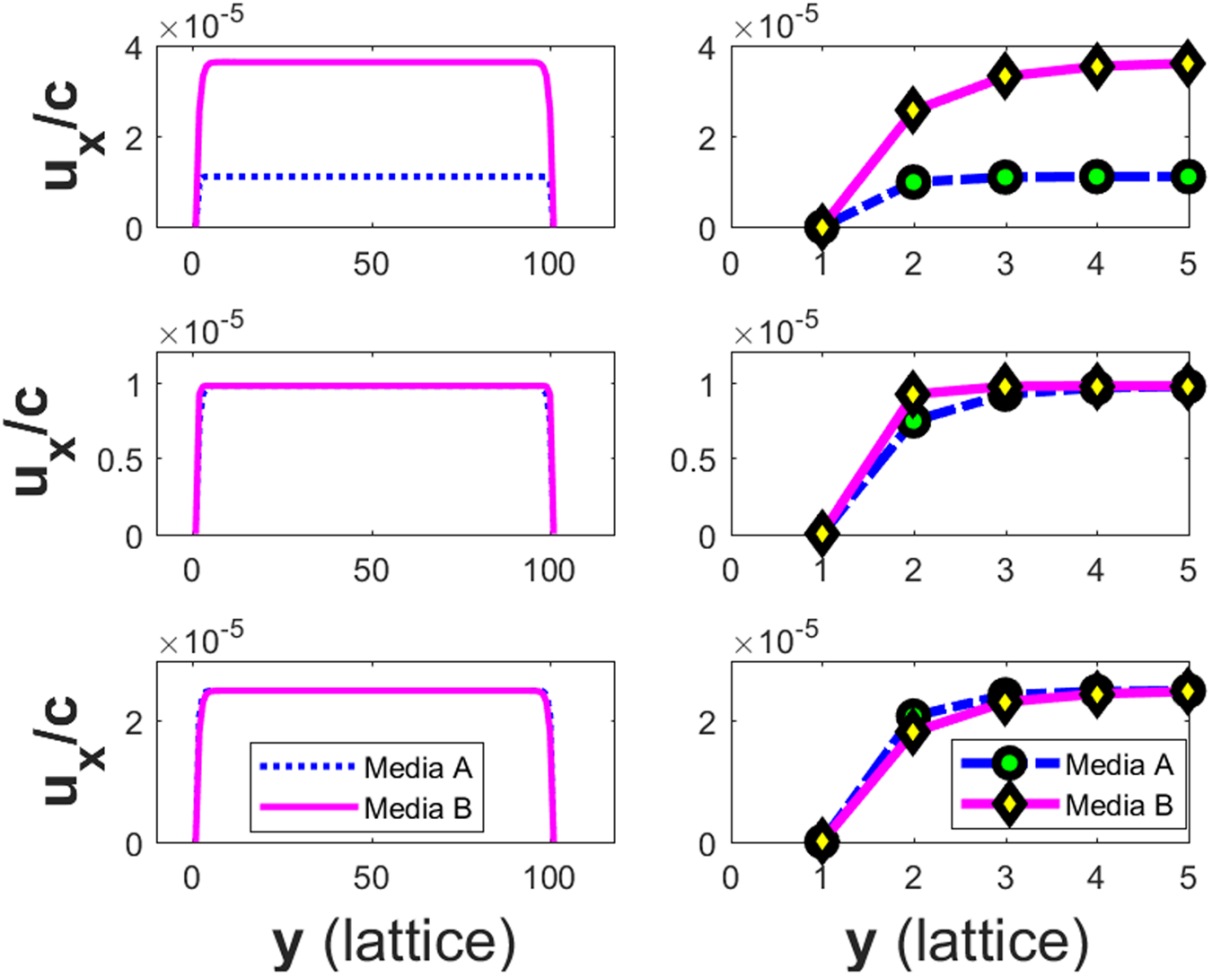
Left column: velocity profiles for Case 1 (Row 1), Case 2 (Row 2), and Case 3 (Row 3); Right column: zoom-in views of the velocity profiles at the left boundary for each case.

Figure 2 shows the velocity profiles for media A and B of Cases 1 to 3 in Table 2 on Rows 1 to 3, respectively. The left column shows an overall view of the profile; the details of the two velocity profiles near the left wall are shown in the right column. Note that the two plateau velocities are quite different because the two configurations lead to different permeability values.

Row 2 in Figure 2 shows the velocity profiles for media A and B in Case 2 on the left. Unlike Case 1, on Row 1, they are very close to each other at their plateaus and differ only near the boundary of the channel, as highlighted on the right. This is because the two configurations lead to similar permeability but different effective viscosity values.

Row 3 in Figure  2 shows the velocity profiles for media A and B in Case 3 on the left. Similar to Case 2, they are very close to each other and actually much closer in this case at their plateaus, but they still differ near the boundary of the channel, as highlighted on the right. Again, this is because the two configurations lead to identical permeability but two different effective viscosity values.

It is of interest to note the relative differences of the velocity profiles close to the boundary in the right column on Row 2 and Row 3. In Case 2, the velocity for medium A is greater than that for B, but the opposite is true in Case 3. These differences reflect precisely the behaviors of the Darcy-Brinkmen flow close to a wall; according to Eq. (16), the velocity is related to the product of the effective viscosity and the curvature of the velocity. In either case, the permeability of both media is similar or identical, and thus, a smaller effective viscosity results in a greater velocity.

## Numerical assessment of model accuracy and applicability

In this section, we assess the accuracy and applicability of our GLBM for modeling the Darcy-Brinkman flow in stratified channel models. In terms of the model accuracy, we compare the simulated velocity profiles of our model with analytical solutions of the Darcy-Brinkman flow. In terms of the model applicability, we consider one specific but critical aspect—the dependence of the simulated flow on the lattice cardinal orientation.

**Figure 3:**
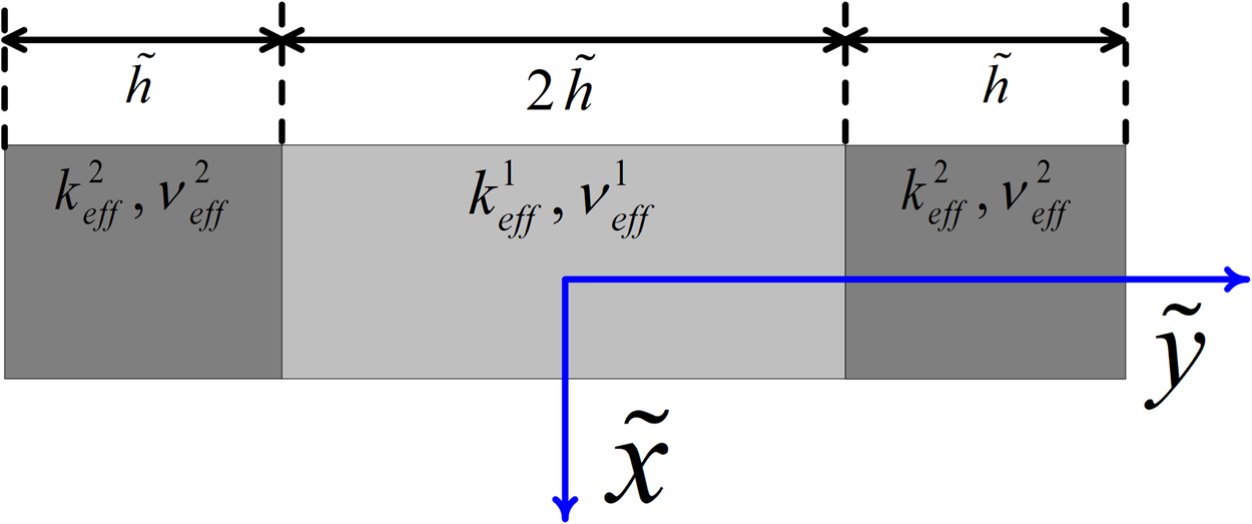
Sketch of a channel filled with two porous media.

We combine the assessment of the accuracy and applicability for two three-layer stratified channel models with the same geometrical configuration shown in Figure 3 but different $({n}_{s},\eta )$ values in Sections 5.1 and 5.2. Note that the middle unshaded layer is media1, defined by $({n}_{s1},{\eta }_{1})$, while the shaded layers at both sides are media2, defined by $({n}_{s2},{\eta }_{2})$. The widths of the three layers are $2\tilde{h}$ for the middle layer and $\tilde{h}$ for both side layers. The two models differ in their ${n}_{s}$ in media1 and media2 with their values switched. Section 5.3 demonstrates that with different combinations of model parameters, the resultant velocity profiles are identical as long as the macroscopic parameters are kept as constants. We provide a strategy to upgrade our model to an exact isotropic version in Section 5.4.

**Figure 4:**
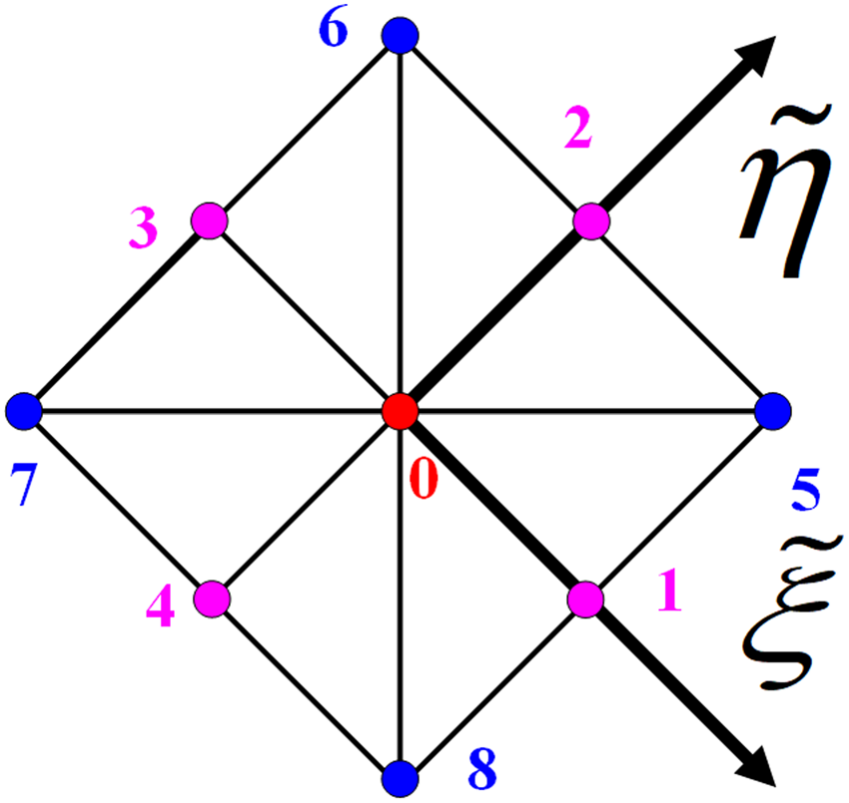
45° diagonal coordinate system.

**Figure 5:**
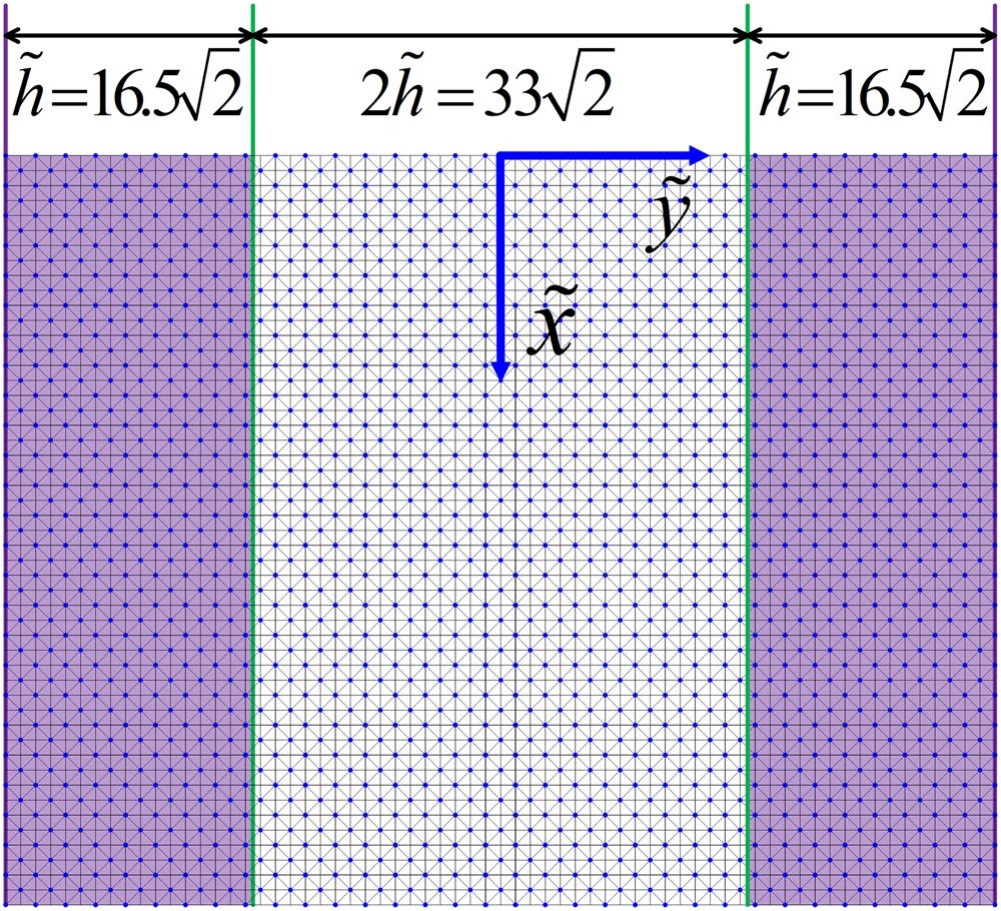
Diagonal lattice setup for a periodical flow boundary channel with the specified channel geometry and discretization (nx = 20, ny = 33).

In Sections 5.1 and 5.2, these two models are discretized using 45° diagonal lattices with different lattice densities. Figure 4 shows a 45° diagonal lattice unit in the lattice coordinate system, where the distance from the local node of 0 to that of 1 is unity, the distance from the local node of 0 to that of 5 is$\sqrt{2}$, and the same 9 particle streaming directions are defined in the local coordinate system $(\tilde{\xi },\tilde{\eta })$. One model is discretized at a low lattice density so that there are 33 diagonal lattice units across, i.e., $ny=33$, and 20 along the channel, i.e., $nx=20$, as shown in Figure  5; the other model is discretized at a high lattice density so that there are 401 or 201 diagonal lattice units across, i.e., $ny={\rm{401}}$ (or 201), and again $nx=20$. Note that each lattice consists of two types of nodes, even and odd, at

17 $$\{\begin{array}{c}\tilde{x}=2\,\ast i\,\,0\le i\le nx\\ \tilde{y}=2\,\ast j\,\,0\le j\le ny\end{array}$$

and

18 $$\{\begin{array}{c}\tilde{x}=2\,\ast i+1\,\,0\le i < nx\\ \tilde{y}=2\,\ast j+1\,\,0\le j < ny\end{array}$$

Note that this setup is convenient for implementing boundary conditions on boundary nodes for the global coordination system $(\tilde{x},\tilde{y})$, although the fluid is streamed following the local coordination system $(\tilde{\xi },\tilde{\eta })$.

In Figure  5, the two green lines mark two respective implicit interfaces between the two porous media, and they are located halfway between the two boundary nodes of media1 and media2 at one side each. The joint nodes were assumed to be located within media2. To achieve a symmetrical velocity profile, media1 contains one more diagonal lattice unit than media2 so that media1 and media2 cover the same channel width $2\tilde{h}$.

In terms of $(\tilde{\xi },\tilde{\eta })$ in Figure 4, the particles stream along 9 directions in the local coordinate system.

19 $$\begin{array}{cccccccccc}{{\bf{e}}}_{\alpha } & 0 & 1 & 2 & 3 & 4 & 5 & 6 & 7 & 8\\ \tilde{\xi } & 0 & 1 & 0 & -1 & 0 & 1 & -1 & -1 & 1\\ \tilde{\eta } & 0 & 0 & 1 & 0 & -1 & 1 & 1 & -1 & -1\end{array}$$

In each model, both periodical flow boundary conditions (PBC) and non-slip no-flux conditions (NSNF) are applied at the channel sides in simulations. Note that along the channel, a periodical boundary condition is always applied. In 5.1 and 5.2, we derive the analytical solutions of velocity and simulated velocities for both models under periodical and non-slip no-flux flow boundary conditions. Table 3 lists all four simulation cases in Sections 5.1 and 5.2. Note that we applied the same body force as in Section 4 in all simulations.

**Table 3:** Simulation cases for stratified channel models.

Case	${{\boldsymbol{n}}}_{{\boldsymbol{s}}1}$	${{\boldsymbol{\eta }}}_{1}$	${{\boldsymbol{n}}}_{{\boldsymbol{s}}2}$	${{\boldsymbol{\eta }}}_{2}$	${\boldsymbol{n}}{\boldsymbol{x}}$	${\boldsymbol{n}}{\boldsymbol{y}}$	BC
1	0.4	0.9	0.2	0.6	20	33	PBC
2	0.2	0.9	0.4	0.6	20	401	PBC
3	0.4	0.9	0.2	0.6	20	33	NSNF
4	0.2	0.9	0.4	0.6	20	201	NSNF

To derive the analytical solution, the Darcy-Brinkman flow is assumed to have been fully developed in every layer of media1 and media2 so that the following equations hold in the respective media:

20 $${v}_{eff}^{1}{\nabla }^{2}{\tilde{u}}_{1}-\frac{{\nu }_{f}}{{k}_{eff}^{1}}{\tilde{u}}_{1}+\frac{{\tilde{F}}_{x}}{\tilde{\rho }}=0$$

21 $${v}_{eff}^{2}{\nabla }^{2}{\tilde{u}}_{2}-\frac{{\nu }_{f}}{{k}_{eff}^{2}}{\tilde{u}}_{2}+\frac{{\tilde{F}}_{x}}{\tilde{\rho }}=0$$

### With a Periodical Boundary Condition

Assuming the channel in Figure 3 has a periodical flow boundary condition, the geometrical symmetry requires the following to hold:

22 $${\frac{d{\tilde{u}}_{1}(\tilde{y})}{d\tilde{y}}|}_{\tilde{y}=0}=0$$

23 $${\frac{d{\tilde{u}}_{2}(\tilde{y})}{d\tilde{y}}|}_{\tilde{y}=\pm 2\tilde{h}}=0$$

Then, define

24 $${\tilde{y}}_{2}=(2\tilde{h}-\tilde{y})$$

and ${\tilde{u}}_{1}(\tilde{y})$ and ${\tilde{u}}_{2}({\tilde{y}}_{2})$ are even functions of $\tilde{y}$ or ${\tilde{y}}_{2}$ at $-\tilde{h}\le \tilde{y}\le \tilde{h}$ and ${\tilde{y}}_{2}$ at $-\tilde{h}\le {\tilde{y}}_{2}\le \tilde{h}$, respectively.

We assume at each interface that the following conditions hold:

25 $${{\tilde{u}}_{1}(\tilde{y})|}_{\tilde{y}=\tilde{h}}={{\tilde{u}}_{2}(\tilde{y})|}_{\tilde{y}=\tilde{h}}$$

26 $${\frac{d{\tilde{u}}_{1}(\tilde{y})}{d\tilde{y}}|}_{\tilde{y}=\tilde{h}}={\frac{d{\tilde{u}}_{2}(\tilde{y})}{d\tilde{y}}|}_{\tilde{y}=\tilde{h}}$$

Then, the exact analytic solution can be found and reads as

27 $$\{\begin{array}{c}{\tilde{u}}_{1}(\tilde{y})={\tilde{u}}_{1}^{0}-\frac{({\tilde{u}}_{1}^{0}-{\tilde{u}}_{2}^{0})\cosh (\sqrt{\frac{{\nu }_{f}}{{k}_{eff}^{1}{v}_{eff}^{1}}}\tilde{y})}{\sinh (\sqrt{\frac{{\nu }_{f}}{{k}_{eff}^{1}{v}_{eff}^{1}}}\tilde{h})[\sqrt{\frac{{k}_{eff}^{2}{v}_{eff}^{2}}{{k}_{eff}^{1}{v}_{eff}^{1}}}\,\coth (\sqrt{\frac{{\nu }_{f}}{{k}_{eff}^{2}{v}_{eff}^{2}}}\tilde{h})+\,\coth (\sqrt{\frac{{\nu }_{f}}{{k}_{eff}^{1}{v}_{eff}^{1}}}\tilde{h})]}\\ -\tilde{h}\le \tilde{y}\le \tilde{h}\end{array}$$

28 $$\{\begin{array}{c}{\tilde{u}}_{2}({\tilde{y}}_{2})={\tilde{u}}_{2}^{0}+\frac{({\tilde{u}}_{1}^{0}-{\tilde{u}}_{2}^{0})\cosh (\sqrt{\frac{{\nu }_{f}}{{k}_{eff}^{2}{v}_{eff}^{2}}}{\tilde{y}}_{2})}{\sinh (\sqrt{\frac{{\nu }_{f}}{{k}_{eff}^{2}{v}_{eff}^{2}}}\tilde{h})[\coth (\sqrt{\frac{{\nu }_{f}}{{k}_{eff}^{2}{v}_{eff}^{2}}}\tilde{h})+\sqrt{\frac{{k}_{eff}^{1}{v}_{eff}^{1}}{{k}_{eff}^{2}{v}_{eff}^{2}}}\,\coth (\sqrt{\frac{{\nu }_{f}}{{k}_{eff}^{1}{v}_{eff}^{1}}}\tilde{h})]}\\ -\tilde{h}\le {\tilde{y}}_{2}\le \tilde{h}\end{array}$$

Note that

29 $${\tilde{u}}_{1}^{0}=\frac{{k}_{eff}^{1}{\tilde{F}}_{x}}{{\nu }_{f}\tilde{\rho }}$$

30 $${\tilde{u}}_{2}^{0}=\frac{{k}_{eff}^{2}{\tilde{F}}_{x}}{{\nu }_{f}\tilde{\rho }}$$

**Figure 6:**
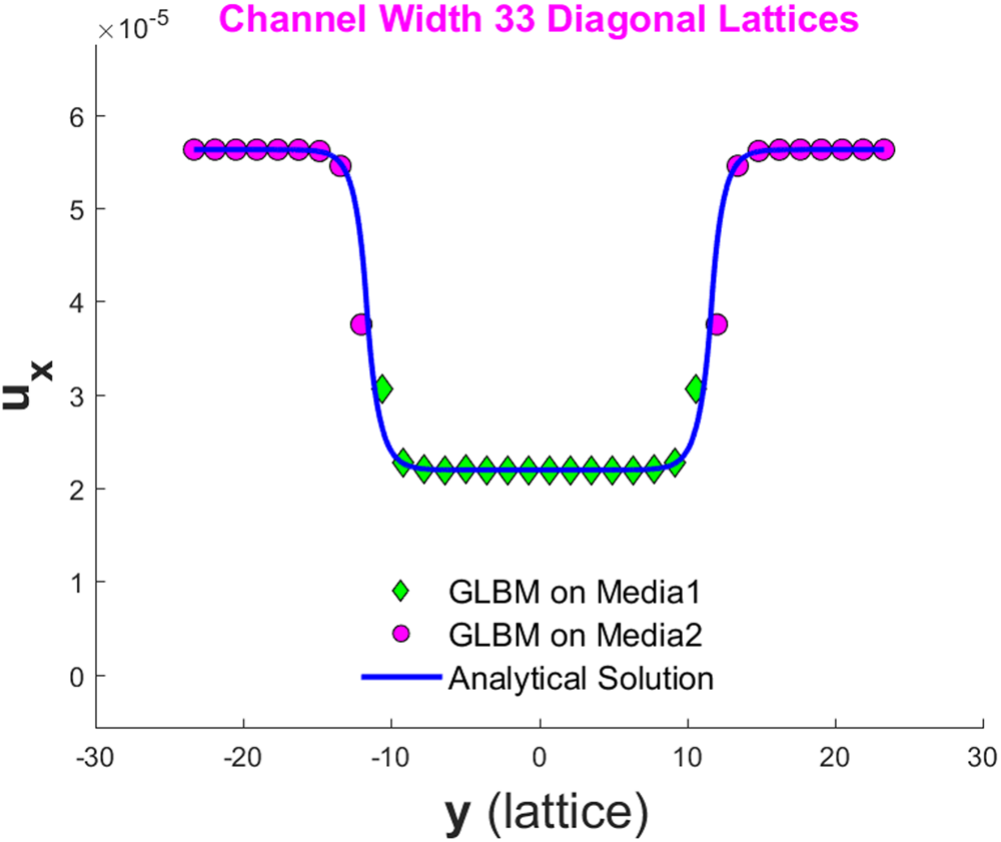
Velocity profile for a periodical boundary channel discretized to have nx = 20 and ny = 33 on a 45° diagonal lattice.

**Figure 7:**
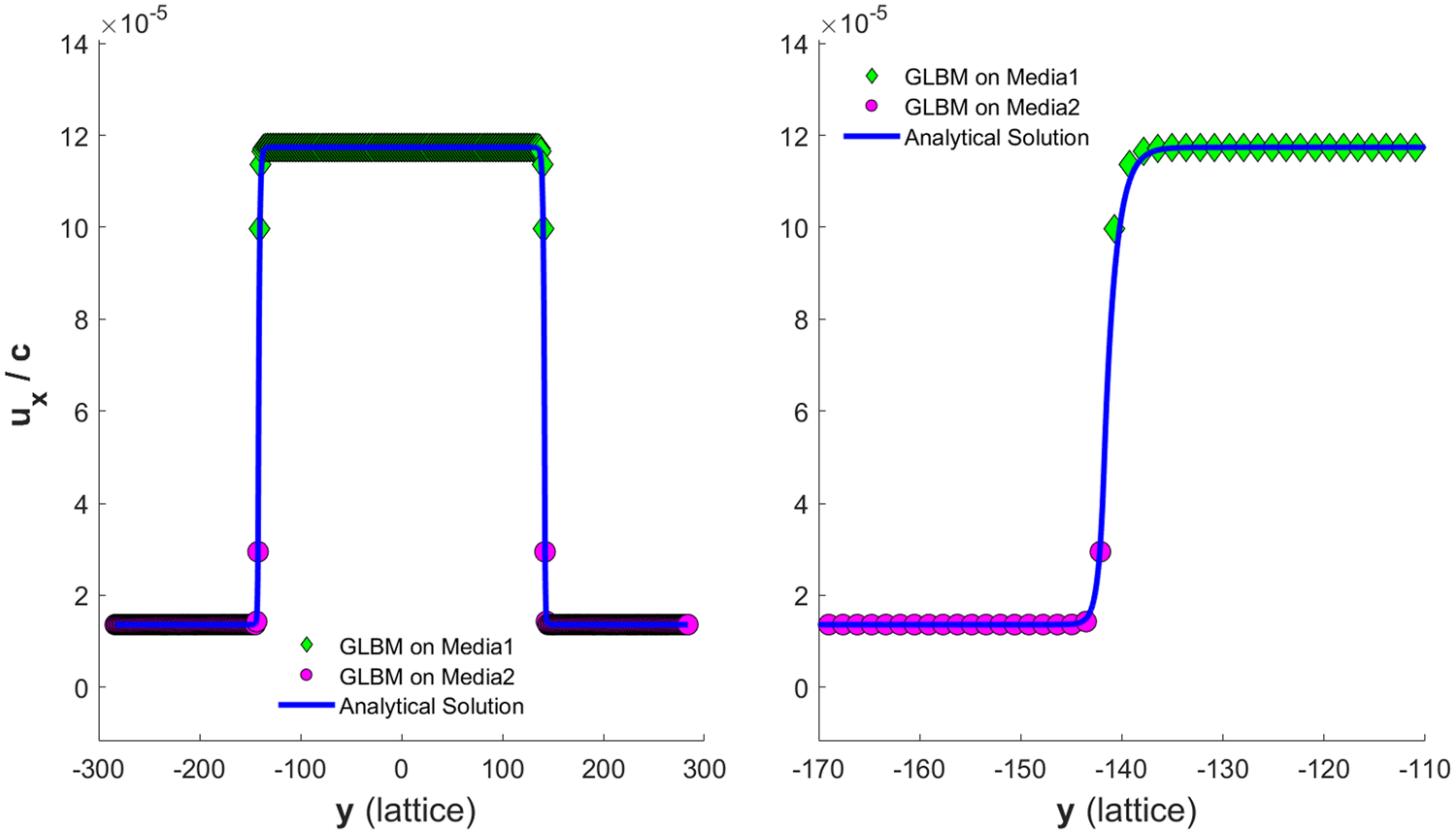
Left: velocity profile for a periodical boundary channel discretized to have nx = 20 and ny = 401 on a 45° diagonal lattice; Right: zoom-in view of the velocity profile about the left interface.

Figure 6 and Figure 7 show the simulated velocity profiles and corresponding analytical solutions for the two models for Cases 1 and 2, respectively. The left of Figure 7 shows the velocity profile for the whole channel, while the right shows the profile details at an interface.

### With a Nonslip Boundary Condition

Assuming the channel flow satisfies the nonslip boundary conditions,

31 $${\tilde{u}}_{2}(-2\tilde{h})={\tilde{u}}_{2}(2\tilde{h})=0$$

the analytical solution reads as

32 $$\{\begin{array}{c}{\tilde{u}}_{1}(\tilde{y})={\tilde{u}}_{1}^{0}-{c}_{1}^{0}\{\exp [\sqrt{\frac{{\nu }_{f}}{{k}_{eff}^{1}{v}_{eff}^{1}}}(\tilde{y}-\tilde{h})]+\exp [-\sqrt{\frac{{\nu }_{f}}{{k}_{eff}^{1}{v}_{eff}^{1}}}(\tilde{y}+\tilde{h})]\}\\ -\tilde{h}\le \tilde{y}\le \tilde{h}\end{array}$$

where

33 $${c}_{1}^{0}=\frac{{\tilde{u}}_{1}^{0}-{\tilde{u}}_{2}^{0}[1-2\exp (-\sqrt{\frac{{\nu }_{f}}{{k}_{eff}^{2}{v}_{eff}^{2}}}h)]}{\sqrt{\frac{{k}_{eff}^{2}{v}_{eff}^{2}}{{k}_{eff}^{1}{v}_{eff}^{1}}}[1-\exp (-\sqrt{\frac{{\nu }_{f}}{{k}_{eff}^{1}{v}_{eff}^{1}}}2\tilde{h})]+[1+\exp (-\sqrt{\frac{{\nu }_{f}}{{k}_{eff}^{1}{v}_{eff}^{1}}}2h)]}$$

34 $$\{\begin{array}{c}{\tilde{u}}_{2}(\tilde{y})={\tilde{u}}_{2}^{0}[1-\exp (-\sqrt{\frac{{\nu }_{f}}{{k}_{eff}^{2}{v}_{eff}^{2}}}{\tilde{y}}_{2})]+{c}_{2}^{0}\{\exp [\sqrt{\frac{{\nu }_{f}}{{k}_{eff}^{2}{v}_{eff}^{2}}}({\tilde{y}}_{2}-\tilde{h})]-\exp [-\sqrt{\frac{{\nu }_{f}}{{k}_{eff}^{2}{v}_{eff}^{2}}}({\tilde{y}}_{2}+\tilde{h})]\}\\ -\tilde{h}\le {\tilde{y}}_{2}\le \tilde{h}\end{array}$$

where

35 $${\tilde{y}}_{2}=(2\tilde{h}-\tilde{y})$$

36 $${c}_{2}^{0}=\frac{{\tilde{u}}_{1}^{0}-{\tilde{u}}_{2}^{0}[1-\exp (-\sqrt{\frac{{\nu }_{f}}{{k}_{eff}^{2}{v}_{eff}^{2}}}\tilde{h})]}{1-\exp (-\sqrt{\frac{{\nu }_{f}}{{k}_{eff}^{2}{v}_{eff}^{2}}}2\tilde{h})}-{c}_{1}^{0}\frac{1+\exp (-\sqrt{\frac{{\nu }_{f}}{{k}_{eff}^{1}{v}_{eff}^{1}}}2\tilde{h})}{1-\exp (-\sqrt{\frac{{\nu }_{f}}{{k}_{eff}^{2}{v}_{eff}^{2}}}2h)\tilde{h}}.$$

**Figure 8:**
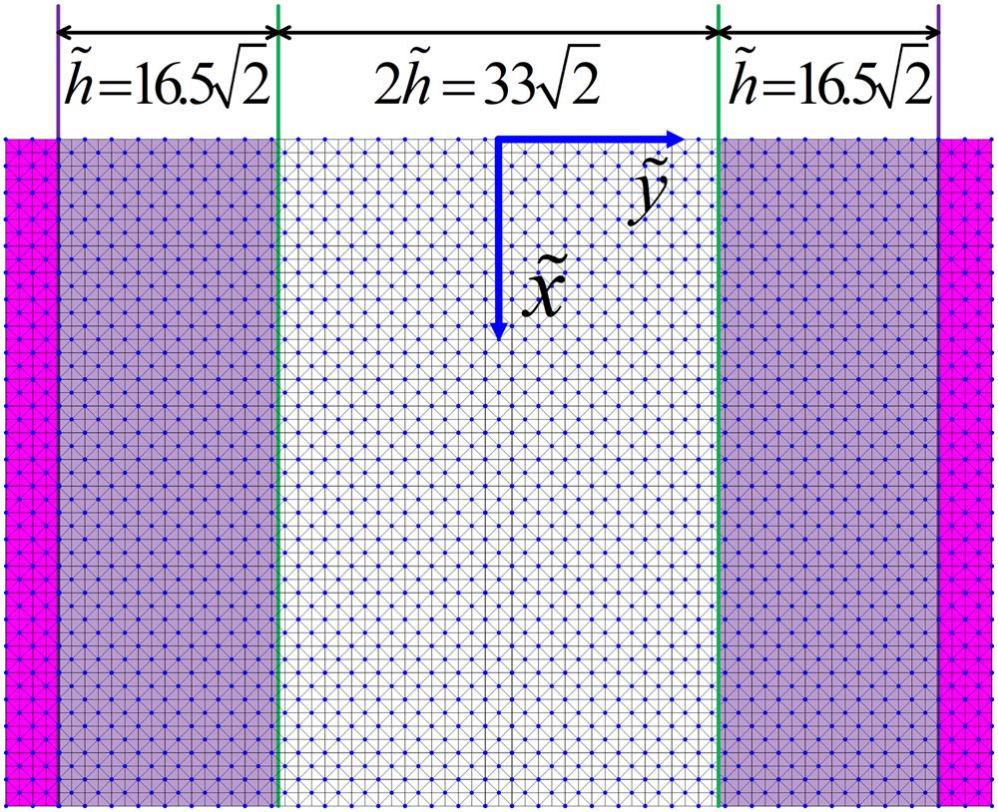
Diagonal lattice setup for a non-slip no-flow boundary condition channel with the specified channel geometry and discretization.

**Figure 9:**
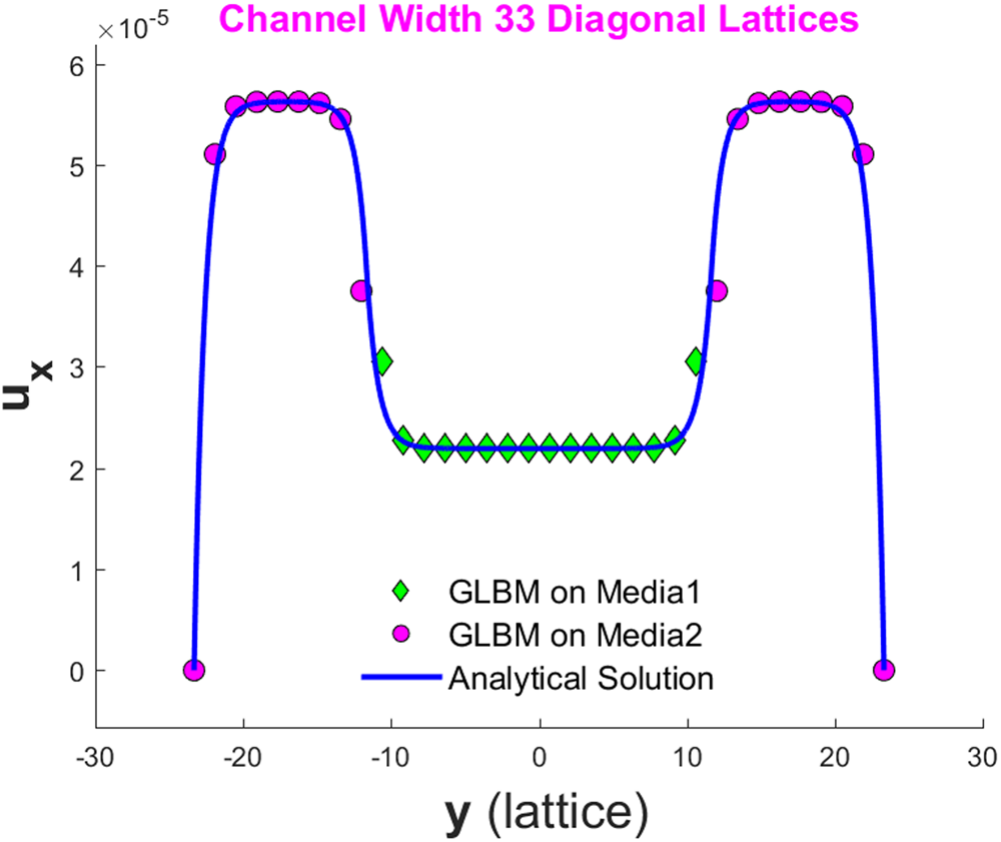
Velocity profile for a non-slip no-flow boundary channel discretized to have nx = 20 and ny = 33 on a 45° diagonal lattice.

**Figure 10:**
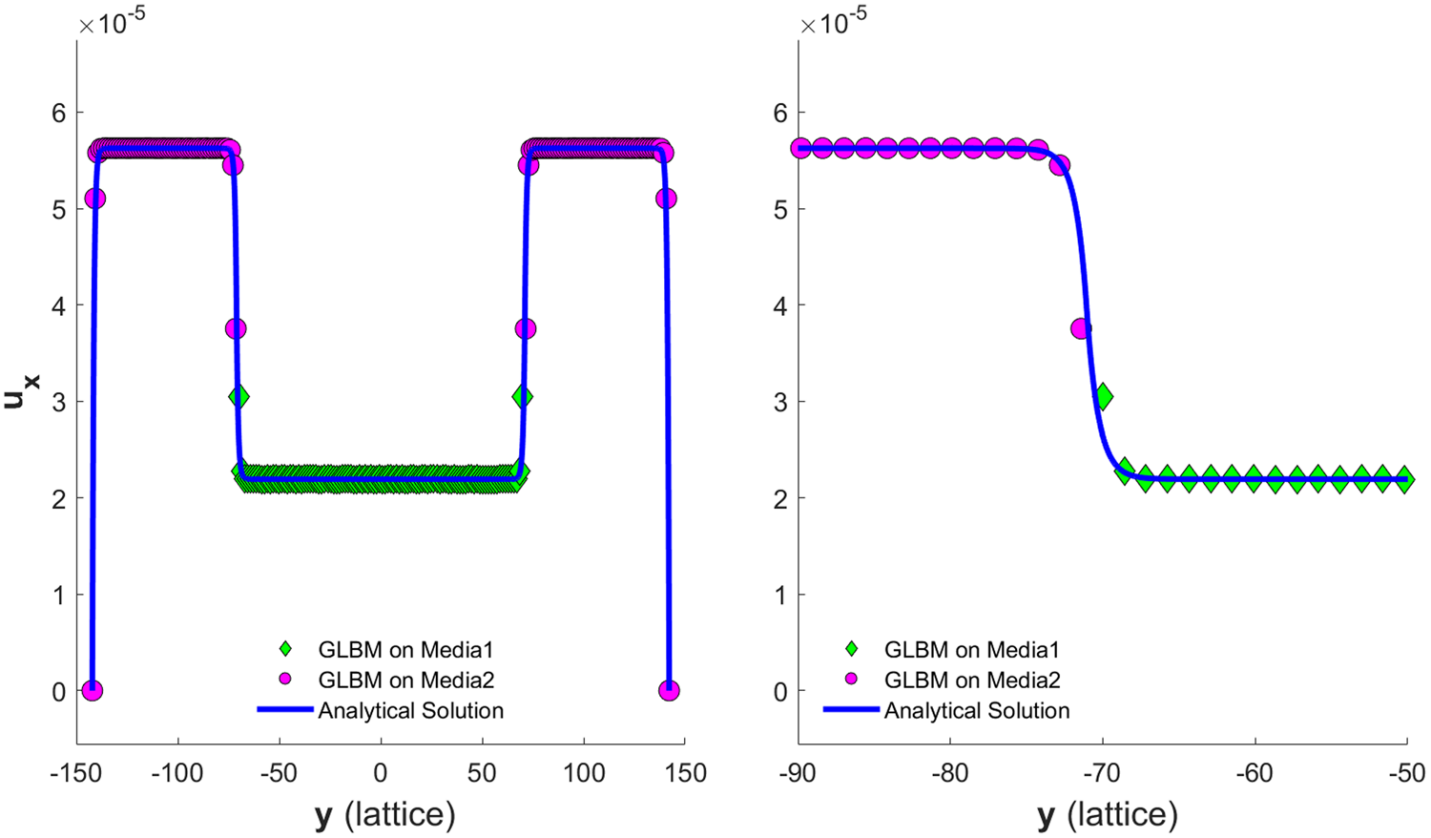
(Left) Velocity profile for a nonslip no-flow boundary channel discretized to have nx = 20 and ny = 201 on a 45° diagonal lattice. (Right) A zoom-in view of the velocity profile around the left interface.

To implement non-slip no-flux boundaries at both sides of the channel, as shown in Figure 8, two more solid diagonal nodes were added to each side of the boundaries. The velocity profiles for Case 3 are shown in Figure 9, while the velocity profiles for Case 4 are shown in Figure 10. The left of Figure 10 shows the velocity profile for the whole channel, while the right shows profile details about the left interface.

Our results above show that the simulated velocity is in good agreement with the analytical solution. This suggests that the anisotropic effect that arises from the rotation of the coordination system is not significant for all the cases we examine here. Note that we do not tune the magnitude of our homogenous body force in any simulation.

### Single Porous Media with a Nonslip Boundary Condition

Nie and Martys^9^ demonstrated that in a Brinkman-Force-Based (BF) LBM scheme, Chapman Enskog expansion does not give an effective viscosity to recover a correct Darcy-Brinkman equation. Note that the effective permeability was prescribed as an input parameter in the BF scheme. However, our model differs from the BF in that, in our model, both the effective viscosity and permeability are not prescribed or imposed but evolved. In what follows, we show that our model indeed produces velocity profiles in very good agreement with analytical profiles. As long as the macroscopic parameters, i.e., $\alpha$ and $r$, are kept constant (see Eq. (38) below), different combinations of the model parameters, as seen in Table 4, lead to exactly the same simulated velocity profile. In this section, we first derive an analytical velocity solution for a channel filled by a single porous media only and then compare that analytical velocity profile with three simulated profiles in which the simulations take different model parameters but constant macroscopic parameters (see Table 4).

**Table 4:** Simulation cases for single porous media channel models.

Case	${\tilde{{\boldsymbol{\tau }}}}^{{\boldsymbol{\ast }}}$	${{\boldsymbol{n}}}_{{\boldsymbol{s}}}$	${\boldsymbol{\eta }}$
1	2.0	0.4	0.6
2	3.0	0.3612	0.4323
3	10.0	0.3008	0.3012

In the case of ${k}_{eff}^{2}\to 0$, we have ${\tilde{u}}_{2}\equiv {\tilde{u}}_{2}^{0}=0$. Eq. (32) reduces to

37 $$\{\begin{array}{c}{\tilde{u}}_{1}(\tilde{y})={\tilde{u}}_{1}^{0}\{1-\frac{\exp [(\tilde{y}-\tilde{h})\sqrt{\frac{{\nu }_{f}}{{k}_{eff}^{1}{v}_{eff}^{1}}}]+\exp [-(\tilde{y}+\tilde{h})\sqrt{\frac{{\nu }_{f}}{{k}_{eff}^{1}{v}_{eff}^{1}}}]}{1+\exp (-2\tilde{h}\sqrt{\frac{{\nu }_{f}}{{k}_{eff}^{1}{v}_{eff}^{1}}})}\}\,\,\,0\le \tilde{y}\le \tilde{h}\\ {\tilde{u}}_{1}(\tilde{y})={\tilde{u}}_{1}(-\tilde{y})\,\,\,\,\,\,\,\,\,\,\,\,\,\,\,\,\,\,\,\,\,\,\,-\tilde{h}\le \tilde{y}\le 0\end{array}$$

For fluid flow in a single porous media channel with a nonslip boundary condition, define the macroscopic model parameters as follows:

38 $$\{\begin{array}{c}{\alpha }_{1}=\sqrt{\frac{{\nu }_{f}}{{k}_{eff}^{1}{v}_{eff}^{1}}}\\ {r}_{1}=\frac{{k}_{eff}^{1}}{{\nu }_{f}}\end{array}$$

Eq. (37) can be rewritten in terms of the macroscopic model parameters as

39 $$\{\begin{array}{c}{\tilde{u}}_{1}(\tilde{y})=\frac{{r}_{1}{\tilde{F}}_{x}}{\tilde{\rho }}\{1-\frac{\exp [(\tilde{y}-\tilde{h}){\alpha }_{1}]+\exp [-(\tilde{y}+\tilde{h}){\alpha }_{1}]}{1+\exp (-2\tilde{h}{\alpha }_{1})}\}\,\,\,0\le \tilde{y}\le \tilde{h}\\ {\tilde{u}}_{1}(\tilde{y})={\tilde{u}}_{1}(-\tilde{y})\,\,\,\,\,\,\,\,\,\,\,\,\,\,\,\,\,-\tilde{h}\le \tilde{y}\le 0\end{array}$$

In Table 4, ${\alpha }_{1}$ and ${r}_{1}$ are fixed with variable model parameters and relaxation time ${\tilde{\tau }}^{\ast }$ as

40 $$\{\begin{array}{c}{\alpha }_{1}=\sqrt{\frac{{\nu }_{f}}{{k}_{eff}^{1}{v}_{eff}^{1}}}=1.8380\\ {r}_{1}=\frac{{k}_{eff}^{1}}{{\nu }_{f}}=0.1364\end{array}$$

**Figure 11:**
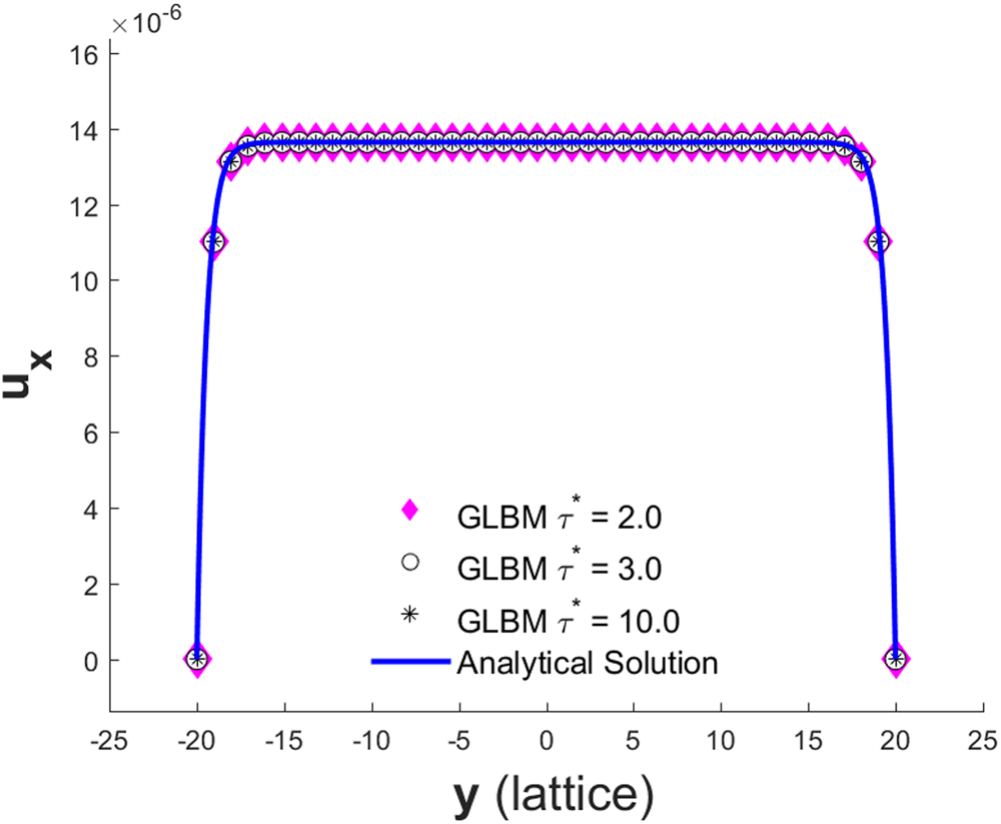
Velocity profile of a single porous media channel for Cases 1 to 3 in Table 4.

Then, the analytic solutions are identical for these three cases, i.e., Eq. (39). The simulation results compared with the corresponding analytic solutions are shown in Figure 11.

We also tested for two porous media channel flows with both periodical and nonslip boundary conditions, using different sets of model parameter combinations as listed in Table 4; all simulations result in the same velocity profile.

### Upgrade to an Isotopic Gray Lattice Boltzmann Model

In previous studies^9^, He’s^13^ derivation method was used to derive the steady-state Darcy-Brinkman equation from the lattice Boltzmann simulation of a generalized Naiver-Stokes equation^8^ with a force term that accounts for the Darcy flow effect of a homogenous porous medium, with fixed porosity and permeability, in a channel. Those authors showed that the effective viscosity coefficient in the steady-state Darcy-Brinkman equation depends on the orientation of the lattice with respect to the channel direction; it takes different values on a lattice that is orientated along a channel direction, referred to as a regular lattice, and on another lattice at 45° clockwise to the channel direction, referred to as diagonal lattice. This anisotropic effect raises a concern regarding the potential impacts on the simulated velocity field when a model is applied to a realistic porous medium where a channel is unlikely to align with the lattice.

We assess this anisotropic effect for the model proposed in this work. Note that in our model, both the effective viscosity and effective permeability are evolved rather than imposed, as in the BF scheme. By performing the same analysis above on our new GLBM, we show that it recovers an isotropic effective permeability but an anisotropic effective viscosity. In what follows, we first analyze the degree of anisotropy with respect to the model parameters and show that the anisotropy has a negligible impact on the velocity fields for practical purposes. Furthermore, we show that we can introduce a strategy to upgrade our new GLBM to an exactly isotropic version, an Isotropic Gray Lattice Boltzmann Model (IGLBM).

**Figure 12:**
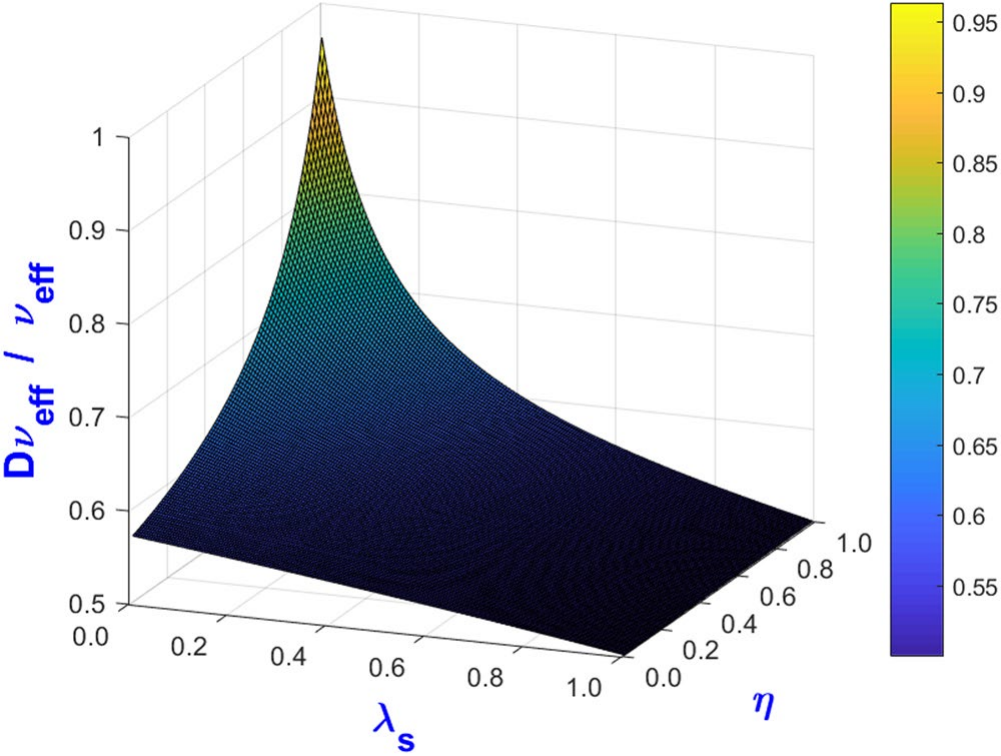
Effective viscosity ratio of GLBM at ${\tilde{\tau }}^{\ast }=2.0$.

Let us define the effective viscosity ratio as

41 $$R=\frac{D{v}_{eff}}{{v}_{eff}}$$

in which $D{v}_{eff}$ is the effective viscosity obtained by GLBM on the diagonal lattice. $D{v}_{eff}$ is determined from Eqs (47) and (49), which are derived in Appendix B in detail. Note that the effective viscosity on a diagonal lattice is always lower than that on a regular lattice, and the effective viscosity ratio satisfies 0.5 ≤ *R* ≤ 1. Figure 12 shows the variation of *R* vs. $\eta$ and ${\lambda }_{s}$, where ${\lambda }_{s}$ is linked to ${n}_{s}$ through Eq. (9). At the upper-left corner of both figures, where R approaches its maximum value of $R={R}_{\max }=1$, the effective viscosity becomes isotropic on both lattices. If the porosity approaches zero, i.e., ${\lambda }_{s}\to 1.0$, $R$ approaches its minimum value of $R\to {R}_{\min }=0.5$, the maximum anisotropy.

**Figure 13:**
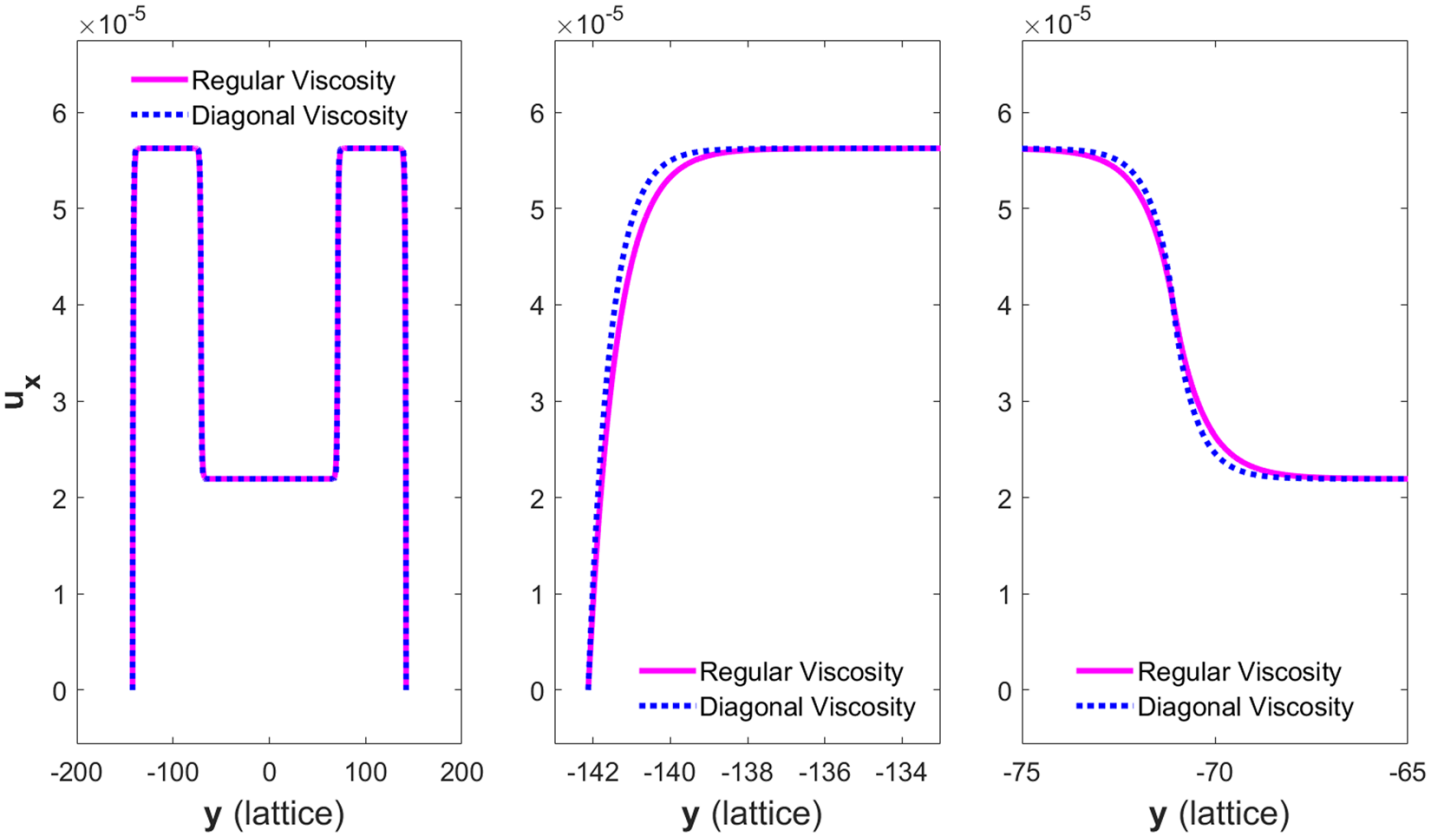
The analytical velocity profiles calculated using effective viscosity *v*_*eff*_ determined on a regular lattice and *Dv*_*eff*_ on a diagonal lattice. Left: overall view; Middle: zoom-in view near the left boundary; Right: zoom-in view about the left interface.

The numerical simulation results presented in the previous sections demonstrated that the velocity profile of GLBM is dominated by the effective permeability, whereas the effective viscosity only affects a very narrow region near a boundary or interface. To illustrate this, we solve Case 4 in Section 5.2 analytically using two effective viscosity values, as determined on the regular lattice and the diagonal lattice. Figure 13 shows that the differences in the velocity are miniscule and only occur at boundares or  the interfaces between the two media. This suggests that if the purpose of the simulation is to determine the velocity profile to perform a volumetric calculation of effective permeability, the anisotropic effect of the effective viscosity may be negligible.

To completely eliminate the anisotropic effect, we can introduce a strategy to upgrade our new GLBM to an exactly isotropic version, an Isotropic Gray Lattice Boltzmann Model (IGLBM). The idea is to relax the assumptions we made in developing GLBM to allow an additional parameter to be introduced to address the anisotropic effect in the effective viscosity. As recalled in Section 2, we have made a very strong assumption that the fraction of fluid particles to be bounced back is equal to the volume fraction of the solid phase. Under this assumption, the model parameter ${n}_{s}$ is linked to the volume fraction of the solid phase ${\lambda }_{s}$. This assumption could be relaxed, as the fraction of fluid particles to be bounced back in each direction $\alpha$ is a function of the solid phase volume, in which the fluid particles may interact during the streaming process along the $\alpha$ direction, ${f}_{\alpha }({V}_{s})$; in general, this function is not only related to the volume of solid phase ${V}_{s}$ but also to the pore geometrical structure ${g}_{s}$, i.e., ${f}_{\alpha }({V}_{s},{g}_{s})$. Then, Eq. (9) can be rewritten as

42 $${n}_{s\alpha }=\frac{{f}_{\alpha }({V}_{s},{g}_{s})}{1+{f}_{\alpha }({V}_{s},{g}_{s})}$$

In Section 3, we have made another strong assumption that the fraction of fluid particles to be bounced back is equal in all 4 pairs of opposite directions. Note that the length of the streaming path in diagonal directions is $\sqrt{2}$times the length in right angle directions of 0° and 90°. Therefore, in principle, the fraction of fluid particles to be bounced back in a diagonal direction should be more than that in the orthogonal directions, as the volume of solid phase met by streaming fluid particles in the diagonal directions is greater than that in the rectangular directions. It is understood that this factor is what induces the anisotropic effective viscosity. To achieve isotropic effective viscosity, we upgrade our model to the following version:

43 $${\tilde{f}}_{\alpha }(\tilde{{\bf{r}}}+{{\bf{e}}}_{\alpha },\tilde{t}+1)=\{\begin{array}{c}\eta [(1-{n}_{1}){\tilde{f}}_{\alpha }^{c}(\tilde{{\bf{r}}},{\tilde{t}}^{\ast })+{n}_{1}\,{\tilde{f}}_{\bar{\alpha }}^{c}(\tilde{{\bf{r}}},{\tilde{t}}^{\ast })]\,(\alpha =1,2,3,4)\\ \eta [(1-{n}_{2}){\tilde{f}}_{\alpha }^{c}(\tilde{{\bf{r}}},{\tilde{t}}^{\ast })+{n}_{2}\,{\tilde{f}}_{\bar{\alpha }}^{c}(\tilde{{\bf{r}}},{\tilde{t}}^{\ast })]\,(\alpha =5,6,7,8)\\ (1-\eta )\tilde{\rho }+\eta \,{\tilde{f}}_{0}^{c}\,\,\,\,\,\,\,\,\,\,\,(\alpha =0)\end{array}$$

in which

44 $$0\le {n}_{1}\le {n}_{2}\le 0.5$$

In Appendix B, we prove that the upgraded model recovers a similar macroscopic Darcy-Brinkman equation as Eq. (11) but with a slightly different coefficient:

45 $$\{\begin{array}{c}A=(1-{\rm{2}}\gamma )(1-{n}_{{\rm{2}}})+\frac{(1-2{n}_{{\rm{2}}})}{6({\tilde{\tau }}^{\ast }-1)}\\ B=\omega (1-{\rm{2}}\gamma )+\frac{(1-2{n}_{{\rm{2}}})}{3}(\frac{\eta }{{\tilde{\tau }}^{\ast }}-\frac{1}{{\tilde{\tau }}^{\ast }-1})\\ C=[{\rm{2}}\gamma \omega -\frac{(1-2{n}_{{\rm{2}}})}{3}(\frac{\eta }{{\tilde{\tau }}^{\ast }}-\frac{1}{{\tilde{\tau }}^{\ast }-1})]{\tilde{\tau }}^{\ast }\end{array}$$

where

46 $$\{\begin{array}{c}\omega =\frac{{\tilde{\tau }}^{\ast }}{\eta ({\tilde{\tau }}^{\ast }-1)}+\eta (1-\frac{1}{{\tilde{\tau }}^{\ast }})(1-2{n}_{{\rm{2}}})-2(1-{n}_{{\rm{2}}})\\ \gamma =\frac{(1-2{n}_{{\rm{1}}})\eta }{3[{\tilde{\tau }}^{\ast }-\eta ({\tilde{\tau }}^{\ast }-1)(1-2{n}_{{\rm{1}}})]}\end{array}$$

If He’s derivation method was carried out on a diagonal lattice for IGLBM at the low Mach number limitation $|{\tilde{u}}_{x}|\ll 1$, the model would also recover a similar macroscopic Darcy-Brinkman equation as Eq. (11); the coefficients are

47 $$\{\begin{array}{c}DA=({\rm{1}}-{n}_{1})(1-2\zeta )+\frac{(1-2{n}_{1})}{3({\tilde{\tau }}^{\ast }-1)}\\ DB=\omega (1-2\zeta )+\frac{2(1-2{n}_{1})}{3}(\frac{\eta }{{\tilde{\tau }}^{\ast }}-\frac{1}{{\tilde{\tau }}^{\ast }-1})\\ DC=2[\omega \zeta -\frac{(1-2{n}_{1})}{3}(\frac{\eta }{{\tilde{\tau }}^{\ast }}-\frac{1}{{\tilde{\tau }}^{\ast }-1})]{\tilde{\tau }}^{\ast }\end{array}$$

where

48 $$\{\begin{array}{c}\omega =(1-2{n}_{1})\eta (1-\frac{1}{{\tilde{\tau }}^{\ast }})+\frac{{\tilde{\tau }}^{\ast }}{\eta ({\tilde{\tau }}^{\ast }-1)}-2(1-{n}_{1})\\ \zeta =\frac{(1-2{n}_{2})\eta }{6[{\tilde{\tau }}^{\ast }-\eta ({\tilde{\tau }}^{\ast }-1)(1-2{n}_{2})]}\end{array}$$

One can define the effective permeability and viscosity on the diagonal lattice as

49 $$D{v}_{eff}=\frac{DA}{2DC}$$

50 $$D{k}_{eff}=\frac{DC}{DB}{\nu }_{f}$$

It can be proved that $D{k}_{eff}\equiv {k}_{eff}$, i.e., the upgraded model always recovers isotropic effective permeability for any ${n}_{1}$ and ${n}_{2}$ that satisfy Eq. (44). By denoting ${n}_{2}$ as ${n}_{s}$ to achieve isotropic effective viscosity, we solve the following equation symbolically:

51 $$D{v}_{eff}(\eta ,{n}_{1},{n}_{s})-{v}_{eff}(\eta ,{n}_{1},{n}_{s})=0$$

which gives

52 $${n}_{1}=\frac{\eta (2{n}_{s}-3{\tilde{\tau }}^{\ast })({\tilde{\tau }}^{\ast }-1)+{\tilde{\tau }}^{\ast }[4{n}_{s}+3({\tilde{\tau }}^{\ast }-1)-6{\tilde{\tau }}^{\ast }{n}_{s}]}{{\tilde{\tau }}^{\ast }-3{({\tilde{\tau }}^{\ast })}^{2}+\eta ({\tilde{\tau }}^{\ast }-1)[2+3{\tilde{\tau }}^{\ast }(2{n}_{s}-3)]}$$

**Figure 14:**
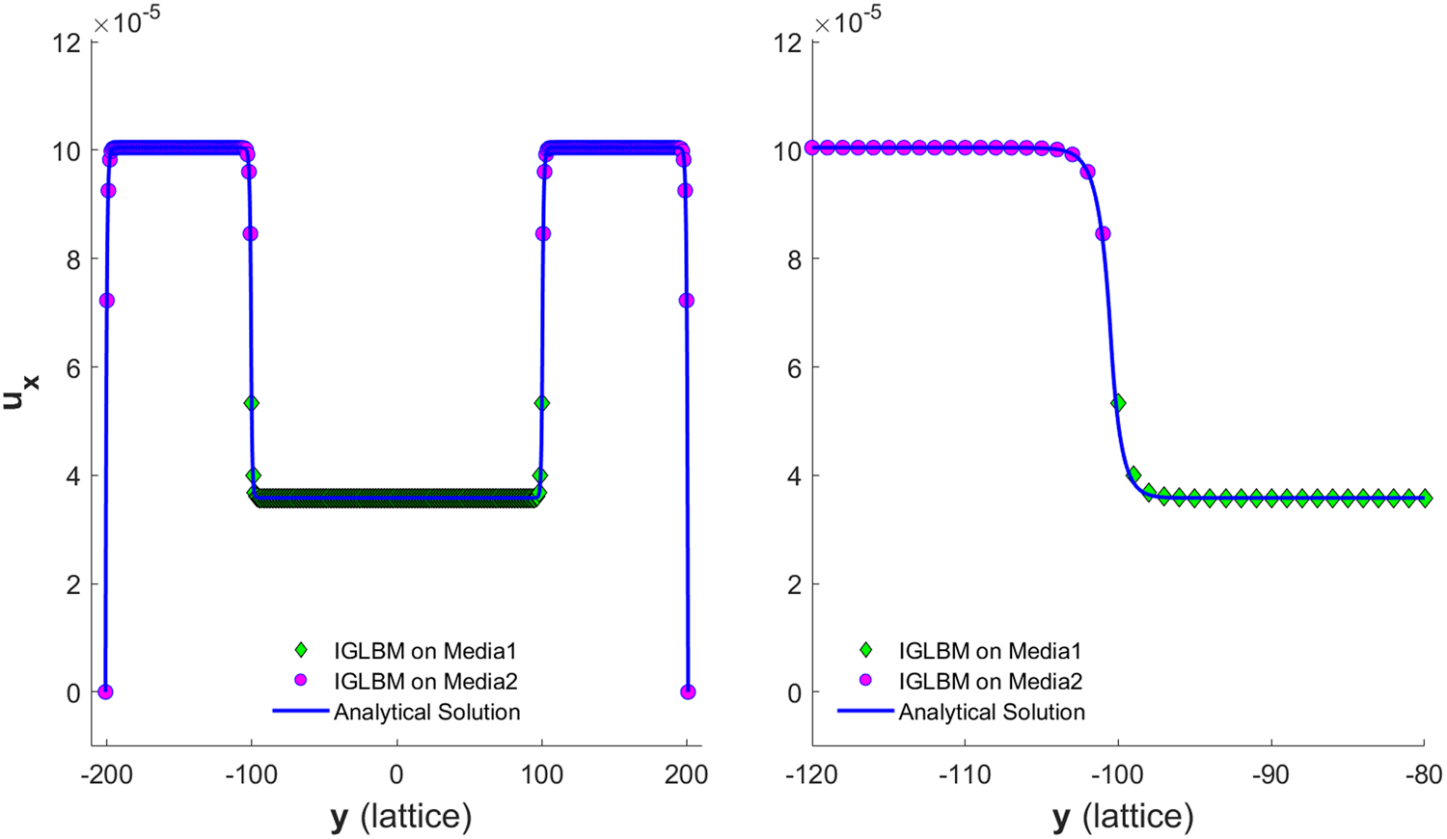
Left: velocity profile of IGLBM in a regular channel; Right: zoom-in view of the velocity profile about the left interface.

By deliberately choosing ${n}_{1}$ according to Eq. (52), IGLBM yields an isotropic effective viscosity. Figure 14 shows a velocity profile of IGLBM with non-slip and no-flux boundary conditions in a channel with two stratified porous media. The model parameters are

53 $${\tilde{\tau }}^{\ast }=2,\,\,({\eta }^{1},{n}_{s}^{1})=(0.8,0.45),\,({\eta }^{1},{n}_{s}^{2})=(0.6,0.2)$$

The corresponding ${n}_{1}^{1}$ and ${n}_{1}^{2}$ values can be calculated through Eq. (52); here, the superscripts stand for porous media 1 and 2.

Note that Eq. (52) is a complex formulation and needs to be evaluated accurately to ensure the accuracy of the numerical simulation. Our test shows that if the equation is evaluated with a long double-precision floating point, the simulation result will be sufficiently accurate.

## Discussion

In the two-parameter model given above, we assume that a post-collision PDF in a direction is first repartitioned into two parts with respect to $(1-{n}_{s})$, along that direction, and ${n}_{s}$, along the opposite direction. Before being streamed, each part is further divided into two subparts with respect to $\eta$ and $(1-\eta )$. The subpart associated with $\eta$ undergoes streaming, while the other subpart sticks to the null-velocity PDF component.

In classical LB, a model is built to represent fluid particle movements at a single length scale, and at each loop (collision and streaming steps), the model has to satisfy mass, momentum and energy conservation. For a GLBM that contains extra repartition steps, it conserves only the mass and not the momentum and kinetic energy. A fraction is lost or dissipated from the grid scale to individual aggregate cells due to the collision of fluid particles with solids at those cells. At each aggregate cell, the collision induces a resistance force that is exerted on fluid particles by the solid and within-cell movements of fluid particles constrained by the local pore structure. At the grid scale, the lost fluid momentum and energy are accounted for in our models by reassigning part of the ready-to-stream fluid particles at each nonzero PDF component (by a factor 1-$\eta$, as defined in Eq. (10)) to the null-velocity PDF component at each aggregate cell.

The momentum and energy losses can be determined for both the single- and two- parameter GLBMs. For the two-parameter model presented above, we can derive Eq. (54) from Eq. (10):

54 $${\tilde{f}}_{\alpha }^{out}(\tilde{{\bf{r}}},{\tilde{t}}^{\ast \ast })+{\tilde{f}}_{\bar{\alpha }}^{out}(\tilde{{\bf{r}}},{\tilde{t}}^{\ast \ast })=\eta [{\tilde{f}}_{\alpha }^{c}(\tilde{{\bf{r}}},{\tilde{t}}^{\ast })+{\tilde{f}}_{\bar{\alpha }}^{c}(\tilde{{\bf{r}}},{\tilde{t}}^{\ast })]$$

Thus, the momentum loss is equal to

55 $${\rm{\Delta }}({{\bf{m}}}_{\alpha }+{{\bf{m}}}_{\tilde{\alpha }})=[(1-\eta )+2{n}_{s}\eta ][{\tilde{f}}_{\alpha }^{c}(\tilde{{\bf{r}}},{\tilde{t}}^{\ast })-{\tilde{f}}_{\bar{\alpha }}^{c}(\tilde{{\bf{r}}},{\tilde{t}}^{\ast })]{{\bf{e}}}_{\alpha }$$

The energy loss is equal to

56 $$\begin{array}{rcl}{\rm{\Delta }}({E}_{\alpha }+{E}_{\tilde{\alpha }}) & = & \{{[{\tilde{f}}_{\alpha }^{c}(\tilde{{\bf{r}}},{\tilde{t}}^{\ast })]}^{2}+{[{\tilde{f}}_{\bar{\alpha }}^{c}(\tilde{{\bf{r}}},{\tilde{t}}^{\ast })]}^{2}\}(1-{\eta }^{2})\\  &  & +\,2{\eta }^{2}(1-{n}_{s}){n}_{s}{[{\tilde{f}}_{\alpha }^{c}(\tilde{{\bf{r}}},{\tilde{t}}^{\ast })-{\tilde{f}}_{\bar{\alpha }}^{c}(\tilde{{\bf{r}}},{\tilde{t}}^{\ast })]}^{2}\end{array}$$

For our single-parameter model, as a special case of the two-parameter model at $\eta =1$, its momentum and energy losses are defined by Eqs (57) and (58), respectively.

57 $${\rm{\Delta }}({{\bf{m}}}_{\alpha }+{{\bf{m}}}_{\tilde{\alpha }})=2{n}_{s}[{\tilde{f}}_{\alpha }^{c}(\tilde{{\bf{r}}},{\tilde{t}}^{\ast })-{\tilde{f}}_{\bar{\alpha }}^{c}(\tilde{{\bf{r}}},{\tilde{t}}^{\ast })]{{\bf{e}}}_{\alpha }$$

58 $${\rm{\Delta }}({E}_{\alpha }+{E}_{\tilde{\alpha }})=2{n}_{s}(1-{n}_{s}){[{\tilde{f}}_{\alpha }^{c}(\tilde{{\bf{r}}},{\tilde{t}}^{\ast })-{\tilde{f}}_{\bar{\alpha }}^{c}(\tilde{{\bf{r}}},{\tilde{t}}^{\ast })]}^{2}$$

To accurately compare the velocity profiles of media A and B, we define the coefficient of momentum loss in Eq. (55) as follows:

59 $${\beta }_{m}=(1-\eta )+2{n}_{s}\eta $$

**Figure 15:**
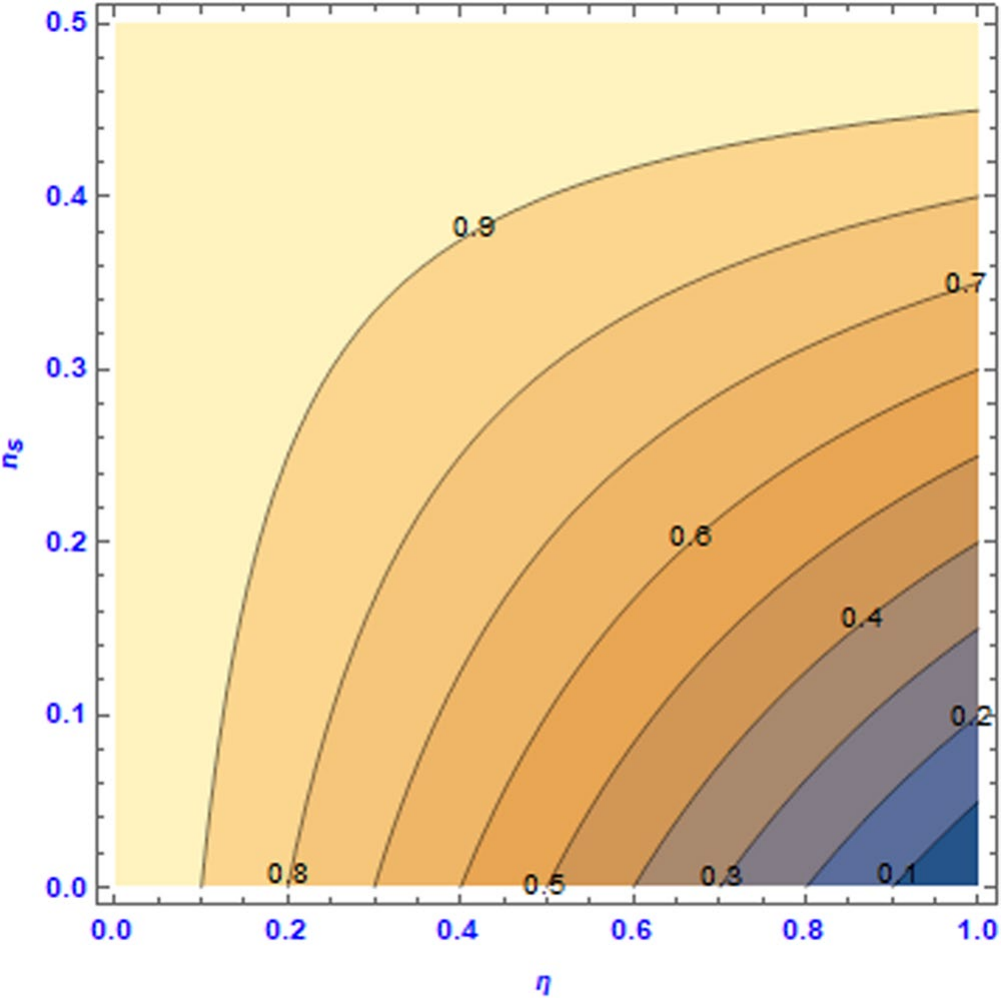
Contours of the momentum loss coefficient *β*_*m*_ with respect to (*n*_*s*_, *η*).

Figure 15 shows the contours of the coefficient of the momentum loss with respect to ${n}_{s}$ and $\eta$. To make a precise analysis of simulated velocity across different model configurations with respect to the effective viscosity and permeability, one may select ${n}_{s}$ and $\eta$ so that ${\beta }_{m}$ falls on the same contour.

In fact, Case 3 in Section 4 is purposely designed by selecting $\eta$ and ${n}_{s}$ pairs so that ${\beta }_{m}$ is equal to 0.8. Therefore, we can conclude unambiguously that the differences in the effective viscosity are solely responsible for the observed velocity differences, rather than different momentum losses.

It should be emphasized that in both the two-parameter GLBM and our single-parameter GLBM^1^, a repartition step is added between the LBM collision and streaming steps. The iterations are as follows: (1) update the state variables, density and velocity using ${\tilde{f}}_{q}^{in}(\tilde{t})$ from the post-streaming step; (2) calculate ${\tilde{f}}_{q}^{eq}(\tilde{t})$ using the state variables; (3) calculate ${\tilde{f}}_{q}^{c}({\tilde{t}}^{\ast })$ based on ${\tilde{f}}_{q}^{eq}(\tilde{t})$ and ${f}_{q}^{in}(t)$ in the collision step; (4) calculate ${\tilde{f}}_{q}^{out}({\tilde{t}}^{\ast \ast })$ in the repartition step; (5) calculate ${\tilde{f}}_{q}^{in}(\tilde{t}+1)$ from ${\tilde{f}}_{q}^{out}({\tilde{t}}^{\ast \ast })$ on all neighbor nodes; and then go back to step (1). The order of the iteration steps is crucially important, as the velocity defined by the incoming PDF is only physically meaningful and is required to calculate ${\tilde{f}}_{q}^{eq}(\tilde{t})$ before ${\tilde{f}}_{q}^{c}({\tilde{t}}^{\ast })$ and ${\tilde{f}}_{q}^{out}({\tilde{t}}^{\ast \ast })$ can be calculated. Therefore, we do not agree with the authors of^14,15^ on their claim that our GLBM^1^ is a special case of their Brinkman-Body-Force-Based (BBF) and Two Relaxation Time (TRT) model^14–16^ because our iteration is different. Our GLBM has only one collision step, the relaxation time ${\tilde{\tau }}^{\ast }$ is the same as the BGK model, and its physical meaning is very clear in gas kinetic theory. More details about the difference between GLBM and BBF can be found in Appendix C with references^17–43^.

Another fundamental difference between GLBM and the BBF^7,8^ model is that both the effective viscosity and effective permeability are evolved from model parameters in GLBM (refer to Appendix A for details), while BBF imposes cell permeability in order to construct a body force to be added to every collision step. The work reported in^9^ demonstrates that the effective viscosity of the BBF scheme depends on the imposed permeability; refer to Eq. (11) and Eq. (12) in their paper for details. If ${k}_{eff}\to 0$, then ${\nu }_{eff}\to \infty$. The nature of the effective viscosity being dependent on the effective permeability in their model is purely caused by imposing a reactive force relating to the Darcy flow as a part of the active body force. This effective viscosity is nonphysical for engineering applications. The work reported in^9^ also demonstrates that the BBF scheme recovers an anisotropic macroscopic equation. The TRT model^16^ introduces a second collision time, which is called a magic parameter, and this additional model parameter can be used to eliminate the anisotropic effective viscosity of the BBF scheme. We show that our GLBM also predicts a weak anisotropic effective viscosity, but it can be upgraded to an exact isotropic version through Eq. (52). Setting ${n}_{1}\ne {n}_{2}={n}_{s}$ is equivalent to introducing one more model parameter, while ${n}_{1}$ plays a very similar role in the isotropic GLBM as the magic parameter in the TRT model^16^. In section 5, Figure 11 shows that as long as the macroscopic parameters on the left-hand side of Eq. (40) are kept as the same, our GLBM predicts the same velocity profile with different model parameters. Figure 1 in reference paper^9^ demonstrates that even if the macroscopic parameters are kept the same, the BBF scheme predicts a different velocity profile with different relaxation time ${\tilde{\tau }}^{\ast }$.

In our previous work^1^, we found that the GLBM of Walsh *et al*.^5^ creates a velocity “jump” across an interface of two permeability-contrasting layers. The “jump” specifically refers to a situation in which the simulated velocities at any pair of lattice nodes nearest to the interface are not only discontinues but also magnitude reversing, meaning that the velocity at the node in the low-permeability medium is greater than that at the node in the high-permeability medium. The author of^14^ claims that our GLBM showed a large ‘jump’ for a case with a high permeability ratio; however, we believe that the author meant that the velocity is discontinuous. The velocities obtained in that work (see Fig. 4(c) of that work)^14^ and in this work (see Figure 7) do not show any magnitude-reversing velocity but only discontinuous velocity. An analytical solution of the Darcy-Brinkman equations (Eqs (27) and (28)) provides a benchmark solution for model comparison. When substituting model parameters into the corresponding equations, we show that$\frac{{\tilde{u}}_{1}(\tilde{h}+0.5/\sqrt{2})-{\tilde{u}}_{2}(\tilde{h}-0.5/\sqrt{2})}{\max ({\tilde{u}}_{1})}\approx 0.5$; this means that our model predicts a velocity consistent with the analytical solution. In Fig. 4(c) of^14^, there are noticeable discrepancies between the simulated velocity and the analytical solution at the interfaces, which we believe are due mainly to too few lattice units being used in the simulation. In addition, we noted that the prescribed periodical boundary condition can incur a very strong fluid interaction at those interfaces. Therefore, we believe the terminology “velocity jump” should be used with caution, as we suggested above, to distinguish fundamental uncharacteristic differences in model behaviors among GLBMs.

## Conclusions

In this paper, first, we have proved that ${n}_{s}=\frac{{\lambda }_{s}}{1+{\lambda }_{s}}$ can hold under repeated bouncing back at local porous structures and therefore confirmed that it is reasonable to estimate ${n}_{s}$ from the local fraction of the solid phase. Second, we introduced a two-parameter model and derived mathematic formulae to determine the effective viscosity and effective permeability. We have shown that the model works well numerically, and the two parameters can be adjusted independently to simulate cases of variable effective viscosity and permeability. The simulated velocity for stratified channel models is shown to be in very good agreement with the analytical solution, even when the channel direction is not aligned with the lattice cardinal direction. We have provided a strategy to upgrade our GLBM to an isotropic version, IGLBM. We have also demonstrated that the effect of anisotropic effective viscosity on the velocity profile is negligible. To avoid the difficulty of model parameter calibration, we believe that it is not necessary to carry out upgrading. We argue that only the uncharacteristic magnitude-reversing velocity across a media interface should be regarded as a ‘jump’.

## Electronic supplementary material

Appendix A, B and C
