# Supplementary material for: Extending a Gray Lattice Boltzmann Model for Simulating Fluid Flow in Multi-Scale Porous Media
Source: Sci Rep. 2018 Apr 11;8:5826. doi: 10.1038/s41598-018-24151-2 (PMC5895624; doi:10.1038/s41598-018-24151-2)
Supplement: Supplementary file 1 — Appendix A, B and C [file 41598_2018_24151_MOESM1_ESM.pdf]

Extending a Gray Lattice Boltzmann Model for Simulating Fluid Flow in Multi-scale Porous Media

Jiujiang Zhu[a], Jingsheng Ma[b]

[a] *School of Civil Engineering, Wuyi University, China*
[b] *Institute of Petroleum Engineering, Heriot-Watt University, Edinburgh, UK*

Appendix A: Effective Viscosity and Permeability of Proposed Model

Two dimension coordinate system and D2Q9 lattice stencil are shown in Figure 1 and Figure 2 respectively

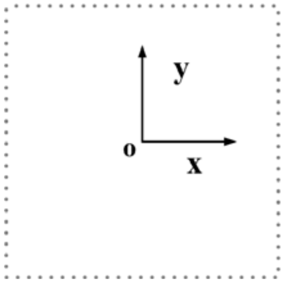

Figure 1 Coordination system

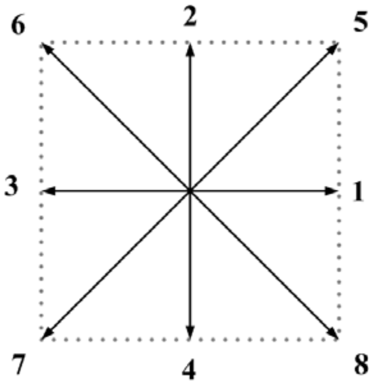

Figure 2 D2Q9 lattice

In coordinate system $\tilde{x}\tilde{y}$

$$\mathbf{e}_\alpha \quad (\alpha = 0,1,\cdots,8) \tag{1}$$

$$\begin{array}{cccccccccc} \mathbf{e} & 0 & 1 & 2 & 3 & 4 & 5 & 6 & 7 & 8 \\ \xi & 0 & 1 & 0 & -1 & 0 & 1 & -1 & -1 & 1 \\ \eta & 0 & 0 & 1 & 0 & -1 & 1 & 1 & -1 & -1 \end{array} \tag{2}$$

$$w_\alpha = \begin{cases} 4/9 & \alpha=0 \\ 1/9 & \alpha=1,2,3,4 \\ 1/36 & \alpha=5,6,7,8 \end{cases} \tag{3}$$

Define

$$\Gamma_\alpha(\tilde{\mathbf{u}}) = w_\alpha \left[1 + \frac{\mathbf{e}_\alpha \cdot \tilde{\mathbf{u}}}{\tilde{\theta}} + \frac{(\mathbf{e}_\alpha \cdot \tilde{\mathbf{u}})^2}{2\tilde{\theta}^2} - \frac{\tilde{\mathbf{u}}^2}{2\tilde{\theta}} \right] \tag{4}$$

Then equilibrium PDF reads

$$\tilde{f}_\alpha^{eq}(\tilde{\mathbf{r}},\tilde{t}) = \tilde{\rho}\Gamma_\alpha(\tilde{\mathbf{u}}) \tag{5}$$

Define collision PDF

$$\tilde{f}_\alpha^{c}(\tilde{\mathbf{r}},\tilde{t}^*) = \left(1-\frac{1}{\tilde{\tau}^*}\right)\tilde{f}_\alpha(\tilde{\mathbf{r}},\tilde{t}) + \left[\frac{\tilde{\rho}}{\tilde{\tau}^*} + \frac{\tilde{\mathbf{F}}\cdot(\mathbf{e}_\alpha - \tilde{\mathbf{u}})}{\tilde{\theta}}\right]\Gamma_\alpha(\tilde{\mathbf{u}}) \tag{6}$$

If $\alpha \neq 0$, our iteration scheme of new GLBM reads

$$\frac{1}{\eta}\tilde{f}_\alpha(\tilde{\mathbf{r}}+\mathbf{e}_\alpha,\tilde{t}+1) = \left[(1-n_s)\tilde{f}_\alpha^{c}(\tilde{\mathbf{r}},\tilde{t}^*) + n_s\tilde{f}_{\bar{\alpha}}^{c}(\tilde{\mathbf{r}},\tilde{t}^*)\right] \tag{7}$$

Substituting Eq. (6) into Eq. (7), iteration scheme for our new GLBM reads

$$\begin{aligned} \frac{1}{\eta}\tilde{f}_\alpha(\tilde{\mathbf{r}}+\mathbf{e}_\alpha,\tilde{t}+1) &= \left(1-\frac{1}{\tilde{\tau}^*}\right)\left[(1-n_s)\tilde{f}_\alpha(\tilde{\mathbf{r}},\tilde{t}) + n_s\tilde{f}_{\bar{\alpha}}(\tilde{\mathbf{r}},\tilde{t})\right] \\ &+ \left[\frac{\tilde{\rho}}{\tilde{\tau}^*} + \frac{\tilde{\mathbf{F}}\cdot(\mathbf{e}_\alpha - \tilde{\mathbf{u}})}{\tilde{\theta}}\right]\Gamma_\alpha(\tilde{\mathbf{u}}) - n_s\left\{\left[\frac{\tilde{\rho}}{\tilde{\tau}^*} + \frac{\tilde{\mathbf{F}}\cdot(\mathbf{e}_\alpha - \tilde{\mathbf{u}})}{\tilde{\theta}}\right]\Gamma_\alpha(\tilde{\mathbf{u}}) - \left[\frac{\tilde{\rho}}{\tilde{\tau}^*} + \frac{\tilde{\mathbf{F}}\cdot(\mathbf{e}_{\bar{\alpha}} - \tilde{\mathbf{u}})}{\tilde{\theta}}\right]\Gamma_{\bar{\alpha}}(\tilde{\mathbf{u}})\right\} \end{aligned} \tag{8}$$

The momentum equation can be expressed as

$$\sum_{\alpha} \tilde{f}_{\alpha} \mathbf{e}_{\alpha} = \sum_{\alpha} \tilde{f}_{\alpha}^{eq} \mathbf{e}_{\alpha} = \tilde{\rho} \tilde{\mathbf{u}} \tag{9}$$

or

$$\left(\tilde{f}_1 - \tilde{f}_3 \right) + \left(\tilde{f}_5 + \tilde{f}_8 - \tilde{f}_6 - \tilde{f}_7 \right) = \tilde{\rho} \tilde{u}_x \tag{10}$$

$$\left(\tilde{f}_2 - \tilde{f}_4 \right) + \left(\tilde{f}_5 + \tilde{f}_6 - \tilde{f}_7 - \tilde{f}_8 \right) = \tilde{\rho} \tilde{u}_y \tag{11}$$

We investigate a steady state flow along x-axis, steady state condition reads

$$\frac{\partial(\ )}{\partial \tilde{t}} = 0, \quad \frac{\partial(\ )}{\partial \tilde{x}} = 0, \quad \tilde{\rho} = const, \quad \tilde{\theta} = const, \quad \tilde{u}_{\tilde{y}} = const = 0, \quad \tilde{u}_x = \tilde{u}_x \left(\tilde{y} \right) \tag{12}$$

Assume $\tilde{\mathbf{F}} = \left\{ \tilde{F}_x, 0 \right\} = \tilde{F}_x \mathbf{e}_1$ and $\tilde{\mathbf{u}} = \left\{ \tilde{u}_x, 0 \right\} = \tilde{u}_x \mathbf{e}_1$, thus

$$\Gamma_{\alpha} \left(\tilde{\mathbf{u}} \right) - \Gamma_{\bar{\alpha}} \left(\tilde{\mathbf{u}} \right) = 2 w_{\alpha} \frac{\mathbf{e}_{\alpha} \cdot \tilde{\mathbf{u}}}{\tilde{\theta}} = 6 w_{\alpha} \left(\mathbf{e}_{\alpha} \cdot \mathbf{e}_1 \right) \tilde{u}_x \tag{13}$$

$$\Gamma_{\alpha} \left(\tilde{\mathbf{u}} \right) + \Gamma_{\bar{\alpha}} \left(\tilde{\mathbf{u}} \right) = 2 w_{\alpha} \left[1 + \frac{\left(\mathbf{e}_{\alpha} \cdot \tilde{\mathbf{u}} \right)^2}{2 \tilde{\theta}^2} - \frac{\tilde{\mathbf{u}}^2}{2 \tilde{\theta}} \right] = w_{\alpha} \left\{ 2 + 3 \tilde{u}_x^2 \left[3 \left(\mathbf{e}_{\alpha} \cdot \mathbf{e}_1 \right)^2 - 1 \right] \right\} \tag{14}$$

$$\begin{aligned} &\left[\frac{\tilde{\rho}}{\tilde{\tau}^*} + \frac{\tilde{\mathbf{F}} \cdot \left(\mathbf{e}_{\alpha} - \tilde{\mathbf{u}} \right)}{\tilde{\theta}} \right] \Gamma_{\alpha} \left(\tilde{\mathbf{u}} \right) - \left[\frac{\tilde{\rho}}{\tilde{\tau}^*} + \frac{\tilde{\mathbf{F}} \cdot \left(\mathbf{e}_{\bar{\alpha}} - \tilde{\mathbf{u}} \right)}{\tilde{\theta}} \right] \Gamma_{\bar{\alpha}} \left(\tilde{\mathbf{u}} \right) \\ &= \frac{6 w_{\alpha} \left(\mathbf{e}_{\alpha} \cdot \mathbf{e}_1 \right)}{\tilde{\tau}^*} \left(\tilde{\rho} \tilde{u}_x + \tilde{\tau}^* \tilde{F}_x \right) - 27 w_{\alpha} \tilde{F}_x \tilde{u}_x^2 \left[1 - \left(\mathbf{e}_{\alpha} \cdot \mathbf{e}_1 \right)^2 \right] \left(\mathbf{e}_{\alpha} \cdot \mathbf{e}_1 \right) \end{aligned} \tag{15}$$

Notice that for any direction α, last term of right hand side of Eq. (15) is always 0, thus

$$\left[\frac{\tilde{\rho}}{\tilde{\tau}^*} + \frac{\tilde{\mathbf{F}} \cdot \left(\mathbf{e}_{\alpha} - \tilde{\mathbf{u}} \right)}{\tilde{\theta}} \right] \Gamma_{\alpha} \left(\tilde{\mathbf{u}} \right) - \left[\frac{\tilde{\rho}}{\tilde{\tau}^*} + \frac{\tilde{\mathbf{F}} \cdot \left(\mathbf{e}_{\bar{\alpha}} - \tilde{\mathbf{u}} \right)}{\tilde{\theta}} \right] \Gamma_{\bar{\alpha}} \left(\tilde{\mathbf{u}} \right) = \frac{6 w_{\alpha} \left(\mathbf{e}_{\alpha} \cdot \mathbf{e}_1 \right)}{\tilde{\tau}^*} \left(\tilde{\rho} \tilde{u}_x + \tilde{\tau}^* \tilde{F}_x \right) \tag{16}$$

Substituting Eq. (16) into Eq. (8), finally we have, if $\alpha \neq 0$, iteration scheme reads

$$\begin{aligned} \frac{1}{\eta} \tilde{f}_{\alpha} \left(\tilde{\mathbf{r}} + \mathbf{e}_{\alpha}, \tilde{t} + 1 \right) &= \left(1 - \frac{1}{\tilde{\tau}^*} \right) \left[\left(1 - n_s \right) \tilde{f}_{\alpha} \left(\tilde{\mathbf{r}}, \tilde{t} \right) + n_s \tilde{f}_{\bar{\alpha}} \left(\tilde{\mathbf{r}}, \tilde{t} \right) \right] \\ &+ \left[\frac{\tilde{\rho}}{\tilde{\tau}^*} + \frac{\tilde{\mathbf{F}} \cdot \left(\mathbf{e}_{\alpha} - \tilde{\mathbf{u}} \right)}{\tilde{\theta}} \right] \Gamma_{\alpha} \left(\tilde{\mathbf{u}} \right) - \frac{6 n_s w_{\alpha} \left(\mathbf{e}_{\alpha} \cdot \mathbf{e}_1 \right)}{\tilde{\tau}^*} \left(\tilde{\rho} \tilde{u}_x + \tilde{\tau}^* \tilde{F}_x \right) \end{aligned} \tag{17}$$

From Eq. (17) we have

$$\frac{1}{\eta}\tilde{f}_1^{\,j}=\left(1-\frac{1}{\tilde{\tau}^*}\right)\left[\left(1-n_s\right)\tilde{f}_1^{\,j}+n_s\tilde{f}_3^{\,j}\right]$$
$$+\left[\frac{\tilde{\rho}}{\tilde{\tau}^*}+\frac{\tilde{\mathbf{F}}\cdot\left(\mathbf{e}_1-\tilde{\mathbf{u}}^j\right)}{\tilde{\theta}}\right]\Gamma_1\left(\mathbf{u}^j\right)-\frac{2n_s}{3\tilde{\tau}^*}\left(\tilde{\rho}\tilde{u}_x^j+\tilde{\tau}^*\tilde{F}_x\right) \tag{18}$$

$$\frac{1}{\eta}\tilde{f}_3^{\,j}=\left(1-\frac{1}{\tilde{\tau}^*}\right)\left[\left(1-n_s\right)\tilde{f}_3^{\,j}+n_s\tilde{f}_1^{\,j}\right]$$
$$+\left[\frac{\tilde{\rho}}{\tilde{\tau}^*}+\frac{\tilde{\mathbf{F}}\cdot\left(\mathbf{e}_3-\tilde{\mathbf{u}}^j\right)}{\tilde{\theta}}\right]\Gamma_3\left(\tilde{\mathbf{u}}^j\right)+\frac{2n_s}{3\tilde{\tau}^*}\left(\tilde{\rho}\tilde{u}_x^j+\tilde{\tau}^*\tilde{F}_x\right) \tag{19}$$

Eq. (18) - Eq. (19) we have

$$\frac{1}{\eta}\left(\tilde{f}_1^{\,j}-\tilde{f}_3^{\,j}\right)=\left(1-\frac{1}{\tilde{\tau}^*}\right)\left(1-2n_s\right)\left(\tilde{f}_1^{\,j}-\tilde{f}_3^{\,j}\right)$$
$$+\left[\frac{\tilde{\rho}}{\tilde{\tau}^*}+\frac{\tilde{\mathbf{F}}\cdot\left(\mathbf{e}_1-\tilde{\mathbf{u}}^j\right)}{\tilde{\theta}}\right]\Gamma_1\left(\mathbf{u}^j\right)-\left[\frac{\tilde{\rho}}{\tilde{\tau}^*}+\frac{\tilde{\mathbf{F}}\cdot\left(\mathbf{e}_3-\tilde{\mathbf{u}}^j\right)}{\tilde{\theta}}\right]\Gamma_3\left(\tilde{\mathbf{u}}^j\right) \tag{20}$$
$$-\frac{4n_s}{3\tilde{\tau}^*}\left(\tilde{\rho}\tilde{u}_x^j+\tilde{\tau}^*\tilde{F}_x\right)$$

Substituting Eq. into Eq. (20)

$$\frac{1}{\eta}\left(\tilde{f}_1^{\,j}-\tilde{f}_3^{\,j}\right)=\left(1-\frac{1}{\tilde{\tau}^*}\right)\left(1-2n_s\right)\left(\tilde{f}_1^{\,j}-\tilde{f}_3^{\,j}\right)+\frac{2\left(1-2n_s\right)}{3\tilde{\tau}^*}\left(\tilde{\rho}\tilde{u}_x^j+\tilde{\tau}^*\tilde{F}_x\right) \tag{21}$$

$$\left(\tilde{f}_1^{\,j}-\tilde{f}_3^{\,j}\right)=2\frac{\left(1-2n_s\right)\eta}{3\left[\tilde{\tau}^*-\eta\left(\tilde{\tau}^*-1\right)\left(1-2n_s\right)\right]}\left(\tilde{\rho}\tilde{u}_x^j+\tilde{\tau}^*\tilde{F}_x\right) \tag{22}$$

Denote

$$\gamma=\frac{\left(1-2n_s\right)\eta}{3\left[\tilde{\tau}^*-\eta\left(\tilde{\tau}^*-1\right)\left(1-2n_s\right)\right]} \tag{23}$$

Eq. (22) can be rewritten as

$$\left(\tilde{f}_1^{\,j}-\tilde{f}_3^{\,j}\right)=2\gamma\left(\tilde{\rho}\tilde{u}_x^j+\tilde{\tau}^*\tilde{F}_x^{\,j}\right) \tag{24}$$

Therefore

$$\left(\tilde{f}_1^{\,j-1} - \tilde{f}_3^{\,j-1} \right) = 2\gamma \left(\tilde{\rho}\tilde{u}_x^{j-1} + \tilde{\tau}^* \tilde{F}_x^{\,j-1} \right) \tag{25}$$

$$\left(\tilde{f}_1^{\,j+1} - \tilde{f}_3^{\,j+1} \right) = 2\gamma \left(\tilde{\rho}\tilde{u}_x^{j+1} + \tilde{\tau}^* \tilde{F}_x^{\,j+1} \right) \tag{26}$$

$$\left(\tilde{f}_1^{\,j-1} - \tilde{f}_3^{\,j-1} \right) + \left(\tilde{f}_1^{\,j+1} - \tilde{f}_3^{\,j+1} \right) = 2\gamma \left[\tilde{\rho} \left(\tilde{u}_x^{j-1} + \tilde{u}_x^{j+1} \right) + \tilde{\tau}^* \left(\tilde{F}_x^{\,j-1} + \tilde{F}_x^{\,j+1} \right) \right] \tag{27}$$

From Eq. (17)

$$\begin{aligned} \frac{1}{\eta} \tilde{f}_5^{\,j+1} &= \left(1 - \frac{1}{\tilde{\tau}^*} \right) \left[\left(1 - n_s \right) \tilde{f}_5^{\,j} + n_s \tilde{f}_7^{\,j} \right] \\ &+ \left[\frac{\tilde{\rho}}{\tilde{\tau}^*} + \frac{\tilde{\mathbf{F}} \cdot \left(\mathbf{e}_5 - \tilde{\mathbf{u}}^j \right)}{\tilde{\theta}} \right] \Gamma_5 \left(\tilde{\mathbf{u}}^j \right) - \frac{n_s}{6\tilde{\tau}^*} \left(\tilde{\rho}\tilde{u}_x^j + \tilde{\tau}^* \tilde{F}_x \right) \end{aligned} \tag{28}$$

$$\begin{aligned} \frac{1}{\eta} \tilde{f}_6^{\,j+1} &= \left(1 - \frac{1}{\tilde{\tau}^*} \right) \left[\left(1 - n_s \right) \tilde{f}_6^{\,j} + n_s \tilde{f}_8^{\,j} \right] \\ &+ \left[\frac{\tilde{\rho}}{\tilde{\tau}^*} + \frac{\tilde{\mathbf{F}} \cdot \left(\mathbf{e}_6 - \tilde{\mathbf{u}}^j \right)}{\tilde{\theta}} \right] \Gamma_6 \left(\tilde{\mathbf{u}}^j \right) + \frac{n_s}{6\tilde{\tau}^*} \left(\tilde{\rho}\tilde{u}_x^j + \tilde{\tau}^* \tilde{F}_x \right) \end{aligned} \tag{29}$$

$$\begin{aligned} \frac{1}{\eta} \tilde{f}_7^{\,j-1} &= \left(1 - \frac{1}{\tilde{\tau}^*} \right) \left[\left(1 - n_s \right) \tilde{f}_7^{\,j} + n_s \tilde{f}_5^{\,j} \right] \\ &+ \left[\frac{\tilde{\rho}}{\tilde{\tau}^*} + \frac{\tilde{\mathbf{F}} \cdot \left(\mathbf{e}_7 - \mathbf{u}^j \right)}{\tilde{\theta}} \right] \Gamma_7 \left(\tilde{\mathbf{u}}^j \right) + \frac{n_s}{6\tilde{\tau}^*} \left(\tilde{\rho}\tilde{u}_x^j + \tilde{\tau}^* \tilde{F}_x \right) \end{aligned} \tag{30}$$

$$\begin{aligned} \frac{1}{\eta} \tilde{f}_8^{\,j-1} &= \left(1 - \frac{1}{\tilde{\tau}^*} \right) \left[\left(1 - n_s \right) \tilde{f}_8^{\,j} + n_s \tilde{f}_6^{\,j} \right] \\ &+ \left[\frac{\tilde{\rho}}{\tilde{\tau}^*} + \frac{\tilde{\mathbf{F}} \cdot \left(\mathbf{e}_8 - \mathbf{u}^j \right)}{\tilde{\theta}} \right] \Gamma_8 \left(\tilde{\mathbf{u}}^j \right) - \frac{n_s}{6\tilde{\tau}^*} \left(\tilde{\rho}\tilde{u}_x^j + \tilde{\tau}^* \tilde{F}_x \right) \end{aligned} \tag{31}$$

Eq. (28) – Eq. (31) maybe rewritten as

$$\begin{aligned} \frac{1}{\eta} \tilde{f}_5^{\,j} &= \left(1 - \frac{1}{\tilde{\tau}^*} \right) \left[\left(1 - n_s \right) \tilde{f}_5^{\,j-1} + n_s \tilde{f}_7^{\,j-1} \right] \\ &+ \left[\frac{\tilde{\rho}}{\tilde{\tau}^*} + \frac{\tilde{\mathbf{F}} \cdot \left(\mathbf{e}_5 - \tilde{\mathbf{u}}^{j-1} \right)}{\tilde{\theta}} \right] \Gamma_5 \left(\tilde{\mathbf{u}}^{j-1} \right) - \frac{n_s}{6\tilde{\tau}^*} \left(\tilde{\rho}\tilde{u}_x^{j-1} + \tilde{\tau}^* \tilde{F}_x \right) \end{aligned} \tag{32}$$

$$\frac{1}{\eta}\tilde{f}_6^{\,j} = \left(1-\frac{1}{\tilde{\tau}^*}\right)\left[\left(1-n_s\right)\tilde{f}_6^{\,j-1} + n_s\tilde{f}_8^{\,j-1}\right] + \left[\frac{\tilde{\rho}}{\tilde{\tau}^*} + \frac{\tilde{\mathbf{F}}\cdot\left(\mathbf{e}_6 - \tilde{\mathbf{u}}^{j-1}\right)}{\tilde{\theta}}\right]\Gamma_6\left(\tilde{\mathbf{u}}^{j-1}\right) + \frac{n_s}{6\tilde{\tau}^*}\left(\tilde{\rho}\tilde{u}_x^{j-1} + \tilde{\tau}^*\tilde{F}_x\right) \tag{33}$$

$$\frac{1}{\eta}\tilde{f}_7^{\,j} = \left(1-\frac{1}{\tilde{\tau}^*}\right)\left[\left(1-n_s\right)\tilde{f}_7^{\,j+1} + n_s\tilde{f}_5^{\,j+1}\right] + \left[\frac{\tilde{\rho}}{\tilde{\tau}^*} + \frac{\tilde{\mathbf{F}}\cdot\left(\mathbf{e}_7 - \mathbf{u}^{j+1}\right)}{\tilde{\theta}}\right]\Gamma_7\left(\tilde{\mathbf{u}}^{j+1}\right) + \frac{n_s}{6\tilde{\tau}^*}\left(\tilde{\rho}\tilde{u}_x^{j+1} + \tilde{\tau}^*\tilde{F}_x\right) \tag{34}$$

$$\frac{1}{\eta}\tilde{f}_8^{\,j} = \left(1-\frac{1}{\tilde{\tau}^*}\right)\left[\left(1-n_s\right)\tilde{f}_8^{\,j+1} + n_s\tilde{f}_6^{\,j+1}\right] + \left[\frac{\tilde{\rho}}{\tilde{\tau}^*} + \frac{\tilde{\mathbf{F}}\cdot\left(\mathbf{e}_8 - \mathbf{u}^{j+1}\right)}{\tilde{\theta}}\right]\Gamma_8\left(\tilde{\mathbf{u}}^{j+1}\right) - \frac{n_s}{6\tilde{\tau}^*}\left(\tilde{\rho}\tilde{u}_x^{j+1} + \tilde{\tau}^*\tilde{F}_x\right) \tag{35}$$

From Eq. (10)

$$\left(\tilde{f}_5^{\,j-1} - \tilde{f}_6^{\,j-1}\right) + \left(\tilde{f}_8^{\,j-1} - \tilde{f}_7^{\,j-1}\right) = \tilde{\rho}\tilde{u}_x^{j-1} - \left(\tilde{f}_1^{\,j-1} - \tilde{f}_3^{\,j-1}\right) \tag{36}$$

$$\left(\tilde{f}_5^{\,j} - \tilde{f}_6^{\,j}\right) + \left(\tilde{f}_8^{\,j} - \tilde{f}_7^{\,j}\right) = \tilde{\rho}\tilde{u}_x^{j} - \left(\tilde{f}_1^{\,j} - \tilde{f}_3^{\,j}\right) \tag{37}$$

$$\left(\tilde{f}_5^{\,j+1} - \tilde{f}_6^{\,j+1}\right) + \left(\tilde{f}_8^{\,j+1} - \tilde{f}_7^{\,j+1}\right) = \tilde{\rho}\tilde{u}_x^{j+1} - \left(\tilde{f}_1^{\,j+1} - \tilde{f}_3^{\,j+1}\right) \tag{38}$$

Eq. (32) - Eq. (33) we have

$$\frac{1}{\eta}\left(\tilde{f}_5^{\,j} - \tilde{f}_6^{\,j}\right) = \left(1-\frac{1}{\tilde{\tau}^*}\right)\left[\left(1-n_s\right)\left(\tilde{f}_5^{\,j-1} - \tilde{f}_6^{\,j-1}\right) - n_s\left(\tilde{f}_8^{\,j-1} - \tilde{f}_7^{\,j-1}\right)\right] + \left[\frac{\tilde{\rho}}{\tilde{\tau}^*} + \frac{\tilde{\mathbf{F}}\cdot\left(\mathbf{e}_5 - \tilde{\mathbf{u}}^{j-1}\right)}{\tilde{\theta}}\right]\Gamma_5\left(\tilde{\mathbf{u}}^{j-1}\right) - \left[\frac{\tilde{\rho}}{\tilde{\tau}^*} + \frac{\tilde{\mathbf{F}}\cdot\left(\mathbf{e}_6 - \tilde{\mathbf{u}}^{j-1}\right)}{\tilde{\theta}}\right]\Gamma_6\left(\tilde{\mathbf{u}}^{j-1}\right) - \frac{n_s}{3\tilde{\tau}^*}\left(\tilde{\rho}\tilde{u}_x^{j-1} + \tilde{\tau}^*\tilde{F}_x\right) \tag{39}$$

$$\frac{1}{\eta}\left(\tilde{f}_5^{\,j} - \tilde{f}_6^{\,j}\right) = \left(1-\frac{1}{\tilde{\tau}^*}\right)\left(\tilde{f}_5^{\,j-1} - \tilde{f}_6^{\,j-1}\right) - \left(1-\frac{1}{\tilde{\tau}^*}\right)n_s\left[\left(\tilde{f}_5^{\,j-1} - \tilde{f}_6^{\,j-1}\right) + \left(\tilde{f}_8^{\,j-1} - \tilde{f}_7^{\,j-1}\right)\right] - \frac{n_s}{3\tilde{\tau}^*}\left(\tilde{\rho}\tilde{u}_x^{j-1} + \tilde{\tau}^*\tilde{F}_x^{j-1}\right) + \frac{\tilde{\rho}}{\tilde{\tau}^*}\left[\Gamma_5\left(\tilde{\mathbf{u}}^{j-1}\right) - \Gamma_6\left(\tilde{\mathbf{u}}^{j-1}\right)\right] + 3\tilde{\mathbf{F}}\cdot\left[\left(\mathbf{e}_5 - \tilde{\mathbf{u}}^{j-1}\right)\Gamma_5\left(\tilde{\mathbf{u}}^{j-1}\right) - \left(\mathbf{e}_6 - \tilde{\mathbf{u}}^{j-1}\right)\Gamma_6\left(\tilde{\mathbf{u}}^{j-1}\right)\right] \tag{40}$$

$$\frac{1}{\eta}\left(\tilde{f}_5^{\,j}-\tilde{f}_6^{\,j}\right)=\left(1-\frac{1}{\tilde{\tau}^*}\right)\left(\tilde{f}_5^{\,j-1}-\tilde{f}_6^{\,j-1}\right)-\left(1-\frac{1}{\tilde{\tau}^*}\right)n_s\left[\left(\tilde{f}_5^{\,j-1}-\tilde{f}_6^{\,j-1}\right)+\left(\tilde{f}_8^{\,j-1}-\tilde{f}_7^{\,j-1}\right)\right]$$
$$-\frac{n_s}{3\tilde{\tau}^*}\left(\tilde{\rho}\tilde{u}_x^{j-1}+\tilde{\tau}^*\tilde{F}_x\right)+\frac{1}{6\tilde{\tau}^*}\left(\tilde{\rho}\tilde{u}_x^{j-1}+\tilde{\tau}^*\tilde{F}_x\right) \tag{41}$$

$$\frac{1}{\eta}\left(\tilde{f}_5^{\,j}-\tilde{f}_6^{\,j}\right)=\left(1-\frac{1}{\tilde{\tau}^*}\right)\left(\tilde{f}_5^{\,j-1}-\tilde{f}_6^{\,j-1}\right)-\left(1-\frac{1}{\tilde{\tau}^*}\right)n_s\left[\left(\tilde{f}_5^{\,j-1}-\tilde{f}_6^{\,j-1}\right)+\left(\tilde{f}_8^{\,j-1}-\tilde{f}_7^{\,j-1}\right)\right]$$
$$+\frac{(1-2n_s)}{6\tilde{\tau}^*}\left(\tilde{\rho}\tilde{u}_x^{j-1}+\tilde{\tau}^*\tilde{F}_x\right) \tag{42}$$

Substituting Eq. (36) into Eq. (42)

$$\frac{1}{\eta}\left(\tilde{f}_5^{\,j}-\tilde{f}_6^{\,j}\right)=\left(1-\frac{1}{\tilde{\tau}^*}\right)\left(\tilde{f}_5^{\,j-1}-\tilde{f}_6^{\,j-1}\right)$$
$$-\left(1-\frac{1}{\tilde{\tau}^*}\right)n_s\left[\tilde{\rho}\tilde{u}_x^{j-1}-\left(\tilde{f}_1^{\,j-1}-\tilde{f}_3^{\,j-1}\right)\right]+\frac{(1-2n_s)}{6\tilde{\tau}^*}\left(\tilde{\rho}\tilde{u}_x^{j-1}+\tilde{\tau}^*\tilde{F}_x\right) \tag{43}$$

$$\frac{1}{\eta}\left(\tilde{f}_5^{\,j+1}-\tilde{f}_6^{\,j+1}\right)=\left(1-\frac{1}{\tilde{\tau}^*}\right)\left(\tilde{f}_5^{\,j}-\tilde{f}_6^{\,j}\right)$$
$$-\left(1-\frac{1}{\tilde{\tau}^*}\right)n_s\left[\tilde{\rho}\tilde{u}_x^{j}-\left(\tilde{f}_1^{\,j}-\tilde{f}_3^{\,j}\right)\right]+\frac{(1-2n_s)}{6\tilde{\tau}^*}\left(\tilde{\rho}\tilde{u}_x^{j}+\tilde{\tau}^*\tilde{F}_x\right) \tag{44}$$

From Eq. (43)

$$\left(1-\frac{1}{\tilde{\tau}^*}\right)\left(\tilde{f}_5^{\,j-1}-\tilde{f}_6^{\,j-1}\right)=\frac{1}{\eta}\left(\tilde{f}_5^{\,j}-\tilde{f}_6^{\,j}\right)$$
$$+\left(1-\frac{1}{\tilde{\tau}^*}\right)n_s\left[\tilde{\rho}\tilde{u}_x^{j-1}-\left(\tilde{f}_1^{\,j-1}-\tilde{f}_3^{\,j-1}\right)\right]-\frac{(1-2n_s)}{6\tilde{\tau}^*}\left(\tilde{\rho}\tilde{u}_x^{j-1}+\tilde{\tau}^*\tilde{F}_x\right) \tag{45}$$

If

$$\tilde{\tau}^*\neq 1 \tag{46}$$

$$\left(\tilde{f}_5^{\,j-1}-\tilde{f}_6^{\,j-1}\right)=\frac{\tilde{\tau}^*}{\eta\left(\tilde{\tau}^*-1\right)}\left(\tilde{f}_5^{\,j}-\tilde{f}_6^{\,j}\right)$$
$$+n_s\left[\tilde{\rho}\tilde{u}_x^{j-1}-\left(\tilde{f}_1^{\,j-1}-\tilde{f}_3^{\,j-1}\right)\right]-\frac{(1-2n_s)}{6\left(\tilde{\tau}^*-1\right)}\left(\tilde{\rho}\tilde{u}_x^{j-1}+\tilde{\tau}^*\tilde{F}_x\right) \tag{47}$$

Eq. (35) - (34) we have

$$\frac{1}{\eta}\left(\tilde{f}_8^{\,j}-\tilde{f}_7^{\,j}\right)=\left(1-\frac{1}{\tilde{\tau}^*}\right)\left[\left(1-n_s\right)\left(\tilde{f}_8^{\,j+1}-\tilde{f}_7^{\,j+1}\right)-n_s\left(\tilde{f}_5^{\,j+1}-\tilde{f}_6^{\,j+1}\right)\right]$$
$$-\frac{n_s}{3\tilde{\tau}^*}\left(\tilde{\rho}\tilde{u}_x^{\,j+1}+\tilde{\tau}^*\tilde{F}_x\right) \tag{48}$$
$$+\left[\frac{\tilde{\rho}}{\tilde{\tau}^*}+\frac{\tilde{\mathbf{F}}\cdot\left(\mathbf{e}_8-\mathbf{u}^{j+1}\right)}{\tilde{\theta}}\right]\Gamma_8\left(\tilde{\mathbf{u}}^{j+1}\right)-\left[\frac{\tilde{\rho}}{\tilde{\tau}^*}+\frac{\tilde{\mathbf{F}}\cdot\left(\mathbf{e}_7-\mathbf{u}^{j+1}\right)}{\tilde{\theta}}\right]\Gamma_7\left(\tilde{\mathbf{u}}^{j+1}\right)$$

$$\frac{1}{\eta}\left(\tilde{f}_8^{\,j}-\tilde{f}_7^{\,j}\right)=\left(1-\frac{1}{\tilde{\tau}^*}\right)\left(\tilde{f}_8^{\,j+1}-\tilde{f}_7^{\,j+1}\right)-\left(1-\frac{1}{\tilde{\tau}^*}\right)n_s\left[\left(\tilde{f}_8^{\,j+1}-\tilde{f}_7^{\,j+1}\right)+\left(\tilde{f}_5^{\,j+1}-\tilde{f}_6^{\,j+1}\right)\right]$$
$$+\frac{\left(1-2n_s\right)}{6\tilde{\tau}^*}\left(\tilde{\rho}\tilde{u}_x^{\,j+1}+\tilde{\tau}^*\tilde{F}_x\right) \tag{49}$$

Substituting Eq. (38) into Eq. (49)

$$\frac{1}{\eta}\left(\tilde{f}_8^{\,j}-\tilde{f}_7^{\,j}\right)=\left(1-\frac{1}{\tilde{\tau}^*}\right)\left(\tilde{f}_8^{\,j+1}-\tilde{f}_7^{\,j+1}\right)-\left(1-\frac{1}{\tilde{\tau}^*}\right)n_s\left[\tilde{\rho}\tilde{u}_x^{\,j+1}-\left(\tilde{f}_1^{\,j+1}-\tilde{f}_3^{\,j+1}\right)\right]$$
$$+\frac{\left(1-2n_s\right)}{6\tilde{\tau}^*}\left(\tilde{\rho}\tilde{u}_x^{\,j+1}+\tilde{\tau}^*\tilde{F}_x\right) \tag{50}$$

Thus

$$\frac{1}{\eta}\left(\tilde{f}_8^{\,j-1}-\tilde{f}_7^{\,j-1}\right)=\left(1-\frac{1}{\tilde{\tau}^*}\right)\left(\tilde{f}_8^{\,j}-\tilde{f}_7^{\,j}\right)-\left(1-\frac{1}{\tilde{\tau}^*}\right)n_s\left[\tilde{\rho}\tilde{u}_x^{\,j}-\left(\tilde{f}_1^{\,j}-\tilde{f}_3^{\,j}\right)\right]$$
$$+\frac{\left(1-2n_s\right)}{6\tilde{\tau}^*}\left(\tilde{\rho}\tilde{u}_x^{\,j}+\tilde{\tau}^*\tilde{F}_x\right) \tag{51}$$

From Eq. (50)

$$\left(1-\frac{1}{\tilde{\tau}^*}\right)\left(\tilde{f}_8^{\,j+1}-\tilde{f}_7^{\,j+1}\right)=\frac{1}{\eta}\left(\tilde{f}_8^{\,j}-\tilde{f}_7^{\,j}\right)+\left(1-\frac{1}{\tilde{\tau}^*}\right)n_s\left[\tilde{\rho}\tilde{u}_x^{\,j+1}-\left(\tilde{f}_1^{\,j+1}-\tilde{f}_3^{\,j+1}\right)\right]$$
$$-\frac{\left(1-2n_s\right)}{6\tilde{\tau}^*}\left(\tilde{\rho}\tilde{u}_x^{\,j+1}+\tilde{\tau}^*\tilde{F}_x\right) \tag{52}$$

if

$$\tilde{\tau}^*\neq 1 \tag{53}$$

$$\left(\tilde{f}_8^{\,j+1}-\tilde{f}_7^{\,j+1}\right)=\frac{\tilde{\tau}^*}{\eta\left(\tilde{\tau}^*-1\right)}\left(\tilde{f}_8^{\,j}-\tilde{f}_7^{\,j}\right)+n_s\left[\tilde{\rho}\tilde{u}_x^{\,j+1}-\left(\tilde{f}_1^{\,j+1}-\tilde{f}_3^{\,j+1}\right)\right]$$
$$-\frac{\left(1-2n_s\right)}{6\left(\tilde{\tau}^*-1\right)}\left(\tilde{\rho}\tilde{u}_x^{\,j+1}+\tilde{\tau}^*\tilde{F}_x\right) \tag{54}$$

From Eq. (44) and Eq. (47)

$$\begin{aligned}\left(\tilde{f}_5^{\,j-1}-\tilde{f}_6^{\,j-1}\right)+\left(\tilde{f}_5^{\,j+1}-\tilde{f}_6^{\,j+1}\right)=\left[\frac{\tilde{\tau}^*}{\eta\left(\tilde{\tau}^*-1\right)}+\frac{\eta\left(\tilde{\tau}^*-1\right)}{\tilde{\tau}^*}\right]\left(\tilde{f}_5^{\,j}-\tilde{f}_6^{\,j}\right)\\+n_s\left[\tilde{\rho}\tilde{u}_x^{j-1}-\left(\tilde{f}_1^{\,j-1}-\tilde{f}_3^{\,j-1}\right)\right]-\eta\left(1-\frac{1}{\tilde{\tau}^*}\right)n_s\left[\tilde{\rho}\tilde{u}_x^{j}-\left(\tilde{f}_1^{\,j}-\tilde{f}_3^{\,j}\right)\right]\\-\frac{\left(1-2n_s\right)}{6\left(\tilde{\tau}^*-1\right)}\left(\tilde{\rho}\tilde{u}_x^{j-1}+\tilde{\tau}^*\tilde{F}_x\right)+\frac{\eta\left(1-2n_s\right)}{6\tilde{\tau}^*}\left(\tilde{\rho}\tilde{u}_x^{j}+\tilde{\tau}^*\tilde{F}_x\right)\end{aligned}\tag{55}$$

From Eq. (51) and Eq. (54)

$$\begin{aligned}\left(\tilde{f}_8^{\,j-1}-\tilde{f}_7^{\,j-1}\right)+\left(\tilde{f}_8^{\,j+1}-\tilde{f}_7^{\,j+1}\right)=\left[\frac{\tilde{\tau}^*}{\eta\left(\tilde{\tau}^*-1\right)}+\frac{\eta\left(\tilde{\tau}^*-1\right)}{\tilde{\tau}^*}\right]\left(\tilde{f}_8^{\,j}-\tilde{f}_7^{\,j}\right)\\+n_s\left[\tilde{\rho}\tilde{u}_x^{j+1}-\left(\tilde{f}_1^{\,j+1}-\tilde{f}_3^{\,j+1}\right)\right]-\eta\left(1-\frac{1}{\tilde{\tau}^*}\right)n_s\left[\tilde{\rho}\tilde{u}_x^{j}-\left(\tilde{f}_1^{\,j}-\tilde{f}_3^{\,j}\right)\right]\\-\frac{\left(1-2n_s\right)}{6\left(\tilde{\tau}^*-1\right)}\left(\tilde{\rho}\tilde{u}_x^{j+1}+\tilde{\tau}^*\tilde{F}_x\right)+\frac{\eta\left(1-2n_s\right)}{6\tilde{\tau}^*}\left(\tilde{\rho}\tilde{u}_x^{j}+\tilde{\tau}^*\tilde{F}_x\right)\end{aligned}\tag{56}$$

From Eq. (36) and Eq. (38)

$$\begin{aligned}\left[\left(\tilde{f}_5^{\,j-1}-\tilde{f}_6^{\,j-1}\right)+\left(\tilde{f}_5^{\,j+1}-\tilde{f}_6^{\,j+1}\right)\right]+\left[\left(\tilde{f}_8^{\,j-1}-\tilde{f}_7^{\,j-1}\right)+\left(\tilde{f}_8^{\,j+1}-\tilde{f}_7^{\,j+1}\right)\right]\\=\tilde{\rho}\left(\tilde{u}_x^{j-1}+\tilde{u}_x^{j+1}\right)-\left[\left(\tilde{f}_1^{\,j-1}-\tilde{f}_3^{\,j-1}\right)+\left(\tilde{f}_1^{\,j+1}-\tilde{f}_3^{\,j+1}\right)\right]\end{aligned}\tag{57}$$

Substituting Eq. (55) and Eq. (56) into Eq. (57)

$$\begin{aligned}\left[\frac{\tilde{\tau}^*}{\eta\left(\tilde{\tau}^*-1\right)}+\frac{\eta\left(\tilde{\tau}^*-1\right)}{\tilde{\tau}^*}\right]\left[\left(\tilde{f}_5^{\,j}-\tilde{f}_6^{\,j}\right)+\left(\tilde{f}_8^{\,j}-\tilde{f}_7^{\,j}\right)\right]\\+n_s\left\{\tilde{\rho}\left(\tilde{u}_x^{j-1}+\tilde{u}_x^{j+1}\right)-\left[\left(\tilde{f}_1^{\,j-1}-\tilde{f}_3^{\,j-1}\right)+\left(\tilde{f}_1^{\,j+1}-\tilde{f}_3^{\,j+1}\right)\right]\right\}\\-2\eta\left(1-\frac{1}{\tilde{\tau}^*}\right)n_s\left[\tilde{\rho}\tilde{u}_x^{j}-\left(\tilde{f}_1^{\,j}-\tilde{f}_3^{\,j}\right)\right]\\+\frac{\eta\left(1-2n_s\right)}{3\tilde{\tau}^*}\left(\tilde{\rho}\tilde{u}_x^{j}+\tilde{\tau}^*\tilde{F}_x\right)-\frac{\left(1-2n_s\right)}{6\left(\tilde{\tau}^*-1\right)}\left[\tilde{\rho}\left(\tilde{u}_x^{j-1}+\tilde{u}_x^{j+1}\right)+2\tilde{\tau}^*\tilde{F}_x\right]\\=\tilde{\rho}\left(\tilde{u}_x^{j-1}+\tilde{u}_x^{j+1}\right)-\left[\left(\tilde{f}_1^{\,j-1}-\tilde{f}_3^{\,j-1}\right)+\left(\tilde{f}_1^{\,j+1}-\tilde{f}_3^{\,j+1}\right)\right]\end{aligned}\tag{58}$$

Substituting Eq. (37) into Eq. (58)

$$
\begin{aligned}
&\left[\frac{\tilde{\tau}^*}{\eta\left(\tilde{\tau}^*-1\right)}+\frac{\eta\left(\tilde{\tau}^*-1\right)\left(1-2n_s\right)}{\tilde{\tau}^*}\right]\left[\tilde{\rho}\tilde{u}_x^j-\left(\tilde{f}_1^{\,j}-\tilde{f}_3^{\,j}\right)\right] \\
&+\frac{\eta\left(1-2n_s\right)}{3\tilde{\tau}^*}\left(\tilde{\rho}\tilde{u}_x^j+\tilde{\tau}^*\tilde{F}_x\right)-\frac{\left(1-2n_s\right)}{6\left(\tilde{\tau}^*-1\right)}\left[\tilde{\rho}\left(\tilde{u}_x^{j-1}+\tilde{u}_x^{j+1}\right)+2\tilde{\tau}^*\tilde{F}_x\right] \\
&=\left(1-n_s\right)\left\{\tilde{\rho}\left(\tilde{u}_x^{j-1}+\tilde{u}_x^{j+1}\right)-\left[\left(\tilde{f}_1^{\,j-1}-\tilde{f}_3^{\,j-1}\right)+\left(\tilde{f}_1^{\,j+1}-\tilde{f}_3^{\,j+1}\right)\right]\right\}
\end{aligned}
\tag{59}
$$

Substituting Eq. (24) - Eq. (27) into Eq. (59) we have

$$
\begin{aligned}
&\left[\frac{\tilde{\tau}^*}{\eta\left(\tilde{\tau}^*-1\right)}+\frac{\eta\left(\tilde{\tau}^*-1\right)\left(1-2n_s\right)}{\tilde{\tau}^*}\right]\left[\tilde{\rho}\tilde{u}_x^j-2\gamma\left(\tilde{\rho}\tilde{u}_x^j+\tilde{\tau}^*\tilde{F}_x\right)\right] \\
&+\frac{\eta\left(1-2n_s\right)}{3\tilde{\tau}^*}\left(\tilde{\rho}\tilde{u}_x^j+\tilde{\tau}^*\tilde{F}_x\right)-\frac{\left(1-2n_s\right)}{6\left(\tilde{\tau}^*-1\right)}\left[\tilde{\rho}\left(\tilde{u}_x^{j-1}+\tilde{u}_x^{j+1}\right)+2\tilde{\tau}^*\tilde{F}_x\right] \\
&=\left(1-n_s\right)\left\{\tilde{\rho}\left(\tilde{u}_x^{j-1}+\tilde{u}_x^{j+1}\right)-2\gamma\left[\tilde{\rho}\left(\tilde{u}_x^{j-1}+\tilde{u}_x^{j+1}\right)+2\tilde{\tau}^*\tilde{F}_x\right]\right\}
\end{aligned}
\tag{60}
$$

$$
\begin{aligned}
&\left[\frac{\tilde{\tau}^*}{\eta\left(\tilde{\tau}^*-1\right)}+\frac{\eta\left(\tilde{\tau}^*-1\right)\left(1-2n_s\right)}{\tilde{\tau}^*}\right]\left(\tilde{\rho}\tilde{u}_x^j\right) \\
&+\left\{\frac{\eta\left(1-2n_s\right)}{3\tilde{\tau}^*}-2\gamma\left[\frac{\tilde{\tau}^*}{\eta\left(\tilde{\tau}^*-1\right)}+\frac{\eta\left(\tilde{\tau}^*-1\right)\left(1-2n_s\right)}{\tilde{\tau}^*}\right]\right\}\left(\tilde{\rho}\tilde{u}_x^j+\tilde{\tau}^*\tilde{F}_x\right) \\
&+\left[\left(2\gamma-1\right)\left(1-n_s\right)-\frac{\left(1-2n_s\right)}{6\left(\tilde{\tau}^*-1\right)}\right]\left[\tilde{\rho}\left(\tilde{u}_x^{j-1}+\tilde{u}_x^{j+1}\right)\right] \\
&+2\left[2\gamma\left(1-n_s\right)-\frac{\left(1-2n_s\right)}{6\left(\tilde{\tau}^*-1\right)}\right]\left(\tilde{\tau}^*\tilde{F}_x\right)=0
\end{aligned}
\tag{61}
$$

$$
\begin{aligned}
&\left[\frac{\tilde{\tau}^*}{\eta\left(\tilde{\tau}^*-1\right)}+\frac{\eta\left(\tilde{\tau}^*-1\right)\left(1-2n_s\right)}{\tilde{\tau}^*}\right]\left(\tilde{\rho}\tilde{u}_x^j\right)\\
&+\left\{\frac{\eta\left(1-2n_s\right)}{3\tilde{\tau}^*}-2\gamma\left[\frac{\tilde{\tau}^*}{\eta\left(\tilde{\tau}^*-1\right)}+\frac{\eta\left(\tilde{\tau}^*-1\right)\left(1-2n_s\right)}{\tilde{\tau}^*}\right]\right\}\left(\tilde{\rho}\tilde{u}_x^j\right)\\
&+\left\{\frac{\eta\left(1-2n_s\right)}{3\tilde{\tau}^*}-2\gamma\left[\frac{\tilde{\tau}^*}{\eta\left(\tilde{\tau}^*-1\right)}+\frac{\eta\left(\tilde{\tau}^*-1\right)\left(1-2n_s\right)}{\tilde{\tau}^*}\right]\right\}\left(\tilde{\tau}^*\tilde{F}_x\right)\\
&+\left[\left(2\gamma-1\right)\left(1-n_s\right)-\frac{\left(1-2n_s\right)}{6\left(\tilde{\tau}^*-1\right)}\right]\left[\tilde{\rho}\left(\tilde{u}_x^{j-1}+\tilde{u}_x^{j+1}\right)\right]\\
&+2\left[2\gamma\left(1-n_s\right)-\frac{\left(1-2n_s\right)}{6\left(\tilde{\tau}^*-1\right)}\right]\left(\tilde{\tau}^*\tilde{F}_x\right)=0
\end{aligned}
\tag{62}
$$

$$
\begin{aligned}
&\left[\frac{\tilde{\tau}^*}{\eta\left(\tilde{\tau}^*-1\right)}+\frac{\eta\left(\tilde{\tau}^*-1\right)\left(1-2n_s\right)}{\tilde{\tau}^*}\right]\left(\tilde{\rho}\tilde{u}_x^j\right)\\
&+\left\{\frac{\eta\left(1-2n_s\right)}{3\tilde{\tau}^*}-2\gamma\left[\frac{\tilde{\tau}^*}{\eta\left(\tilde{\tau}^*-1\right)}+\frac{\eta\left(\tilde{\tau}^*-1\right)\left(1-2n_s\right)}{\tilde{\tau}^*}\right]\right\}\left(\tilde{\rho}\tilde{u}_x^j\right)\\
&+2\left[\left(2\gamma-1\right)\left(1-n_s\right)-\frac{\left(1-2n_s\right)}{6\left(\tilde{\tau}^*-1\right)}\right]\left(\tilde{\rho}\tilde{u}_x^j\right)\\
&+\left\{\frac{\eta\left(1-2n_s\right)}{3\tilde{\tau}^*}-2\gamma\left[\frac{\tilde{\tau}^*}{\eta\left(\tilde{\tau}^*-1\right)}+\frac{\eta\left(\tilde{\tau}^*-1\right)\left(1-2n_s\right)}{\tilde{\tau}^*}\right]\right\}\left(\tilde{\tau}^*\tilde{F}_x\right)\\
&+2\left[2\gamma\left(1-n_s\right)-\frac{\left(1-2n_s\right)}{6\left(\tilde{\tau}^*-1\right)}\right]\left(\tilde{\tau}^*\tilde{F}_x\right)\\
&+\left[\left(2\gamma-1\right)\left(1-n_s\right)-\frac{\left(1-2n_s\right)}{6\left(\tilde{\tau}^*-1\right)}\right]\left[\tilde{\rho}\left(\tilde{u}_x^{j-1}+\tilde{u}_x^{j+1}-2\tilde{u}_x^j\right)\right]=0
\end{aligned}
\tag{63}
$$

$$
+\left\{\begin{array}{c}\dfrac{\eta(1-2n_s)}{3\tilde{\tau}^*}-(2\gamma-1)\left[\dfrac{\tilde{\tau}^*}{\eta(\tilde{\tau}^*-1)}+\dfrac{\eta(\tilde{\tau}^*-1)(1-2n_s)}{\tilde{\tau}^*}\right]\\ +2\left[(2\gamma-1)(1-n_s)-\dfrac{(1-2n_s)}{6(\tilde{\tau}^*-1)}\right]\end{array}\right\}\left(\tilde{\rho}\tilde{u}_x^j\right)
$$

$$
+\left\{2\left[2\gamma(1-n_s)-\frac{(1-2n_s)}{6(\tilde{\tau}^*-1)}\right]+\frac{\eta(1-2n_s)}{3\tilde{\tau}^*}-2\gamma\left[\frac{\tilde{\tau}^*}{\eta(\tilde{\tau}^*-1)}+\frac{\eta(\tilde{\tau}^*-1)(1-2n_s)}{\tilde{\tau}^*}\right]\right\}\left(\tilde{\tau}^*\tilde{F}_x\right)
$$

$$
+\left[(2\gamma-1)(1-n_s)-\frac{(1-2n_s)}{6(\tilde{\tau}^*-1)}\right]\left[\tilde{\rho}\left(\tilde{u}_x^{j-1}+\tilde{u}_x^{j+1}-2\tilde{u}_x^j\right)\right]=0 \tag{64}
$$

$$
+\left\{\frac{\eta(1-2n_s)}{3\tilde{\tau}^*}-(2\gamma-1)\left[\frac{\tilde{\tau}^*}{\eta(\tilde{\tau}^*-1)}+\frac{\eta(\tilde{\tau}^*-1)(1-2n_s)}{\tilde{\tau}^*}-2(1-n_s)\right]-\frac{(1-2n_s)}{3(\tilde{\tau}^*-1)}\right\}\left(\tilde{\rho}\tilde{u}_x^j\right)
$$

$$
+\left\{\frac{\eta(1-2n_s)}{3\tilde{\tau}^*}-2\gamma\left[\frac{\tilde{\tau}^*}{\eta(\tilde{\tau}^*-1)}+\frac{\eta(\tilde{\tau}^*-1)(1-2n_s)}{\tilde{\tau}^*}-2(1-n_s)\right]-\frac{(1-2n_s)}{3(\tilde{\tau}^*-1)}\right\}\left(\tilde{\tau}^*\tilde{F}_x\right)
$$

$$
+\left[(2\gamma-1)(1-n_s)-\frac{(1-2n_s)}{6(\tilde{\tau}^*-1)}\right]\left[\tilde{\rho}\left(\tilde{u}_x^{j-1}+\tilde{u}_x^{j+1}-2\tilde{u}_x^j\right)\right]=0 \tag{65}
$$

Denote

$$
\omega=\frac{\tilde{\tau}^*}{\eta(\tilde{\tau}^*-1)}+\eta\left(1-\frac{1}{\tilde{\tau}^*}\right)(1-2n_s)-2(1-n_s) \tag{66}
$$

Then Eq. (65) can be rewritten as

$$
\left[(1-2\gamma)(1-n_s)+\frac{(1-2n_s)}{6(\tilde{\tau}^*-1)}\right]\tilde{\rho}\left(\tilde{u}_x^{j-1}+\tilde{u}_x^{j+1}-2\tilde{u}_x^j\right)
$$

$$
-\left[\omega(1-2\gamma)+\frac{(1-2n_s)}{3}\left(\frac{\eta}{\tilde{\tau}^*}-\frac{1}{\tilde{\tau}^*-1}\right)\right]\left(\tilde{\rho}\tilde{u}_x^j\right) \tag{67}
$$

$$
+\left[2\gamma\omega-\frac{(1-2n_s)}{3}\left(\frac{\eta}{\tilde{\tau}^*}-\frac{1}{\tilde{\tau}^*-1}\right)\right]\tilde{\tau}^*\left(\tilde{F}_x\right)=0
$$

Denote

$$\begin{cases} A = (1-2\gamma)(1-n_s) + \dfrac{(1-2n_s)}{6(\tilde{\tau}^*-1)} \\ B = \omega(1-2\gamma) + \dfrac{(1-2n_s)}{3}\left(\dfrac{\eta}{\tilde{\tau}^*} - \dfrac{1}{\tilde{\tau}^*-1}\right) \\ C = \left[2\gamma\omega - \dfrac{(1-2n_s)}{3}\left(\dfrac{\eta}{\tilde{\tau}^*} - \dfrac{1}{\tilde{\tau}^*-1}\right)\right]\tilde{\tau}^* \end{cases} \tag{68}$$

Then Eq. (67) can be rewritten as

$$A\left(\tilde{u}_x^{j-1} + \tilde{u}_x^{j+1} - 2\tilde{u}_x^j\right) - B\tilde{u}_x^j + C\frac{\tilde{F}_x^{\,j}}{\tilde{\rho}} = 0 \tag{69}$$

Extending a Gray Lattice Boltzmann Model for Simulating Fluid Flow in Multi-scale Porous Media

Jiujiang Zhu[a], Jingsheng Ma[b]

[a] *School of Civil Engineering, Wuyi University, China*
[b] *Institute of Petroleum Engineering, Heriot-Watt University, Edinburgh, UK*

Appendix B: Effective Viscosity and Permeability for $n_1 \neq n_2$

Two dimension coordinate system and D2Q9 lattice stencil are shown in Figure 1 and Figure 2 respectively

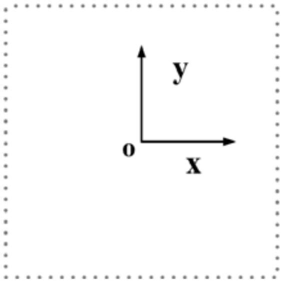

Figure 1 Coordination system

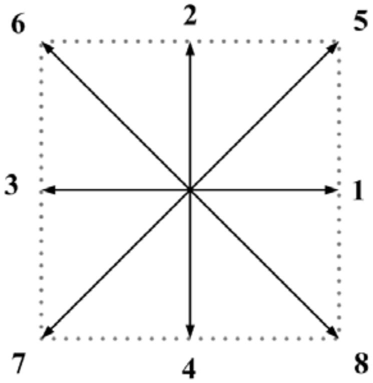

Figure 2 D2Q9 lattice

In coordinate system $\tilde{x}\tilde{y}$

$$\mathbf{e}_\alpha \quad (\alpha = 0,1,\cdots,8) \tag{1}$$

$$\begin{array}{cccccccccc} \mathbf{e} & 0 & 1 & 2 & 3 & 4 & 5 & 6 & 7 & 8 \\ \xi & 0 & 1 & 0 & -1 & 0 & 1 & -1 & -1 & 1 \\ \eta & 0 & 0 & 1 & 0 & -1 & 1 & 1 & -1 & -1 \end{array} \tag{2}$$

$$w_\alpha = \begin{cases} 4/9 & \alpha=0 \\ 1/9 & \alpha=1,2,3,4 \\ 1/36 & \alpha=5,6,7,8 \end{cases} \tag{3}$$

Define

$$\Gamma_\alpha(\tilde{\mathbf{u}}) = w_\alpha \left[1 + \frac{\mathbf{e}_\alpha \cdot \tilde{\mathbf{u}}}{\tilde{\theta}} + \frac{(\mathbf{e}_\alpha \cdot \tilde{\mathbf{u}})^2}{2\tilde{\theta}^2} - \frac{\tilde{\mathbf{u}}^2}{2\tilde{\theta}}\right] \tag{4}$$

Then equilibrium PDF reads

$$\tilde{f}_\alpha^{eq}(\tilde{\mathbf{r}},\tilde{t}) = \tilde{\rho}\Gamma_\alpha(\tilde{\mathbf{u}}) \tag{5}$$

Define collision PDF

$$\tilde{f}_\alpha^{c}(\tilde{\mathbf{r}},\tilde{t}^*) = \left(1 - \frac{1}{\tilde{\tau}^*}\right)\tilde{f}_\alpha(\tilde{\mathbf{r}},\tilde{t}) + \left[\frac{\tilde{\rho}}{\tilde{\tau}^*} + \frac{\tilde{\mathbf{F}}\cdot(\mathbf{e}_\alpha - \tilde{\mathbf{u}})}{\tilde{\theta}}\right]\Gamma_\alpha(\tilde{\mathbf{u}}) \tag{6}$$

If $\alpha \neq 0$, our iteration scheme of new GLBM reads

$$\tilde{f}_\alpha(\tilde{\mathbf{r}} + \mathbf{e}_\alpha, \tilde{t}+1) = \begin{cases} \eta\left[(1-n_1)\tilde{f}_\alpha^c(\tilde{\mathbf{r}},\tilde{t}^*) + n_1\tilde{f}_{\bar{\alpha}}^c(\tilde{\mathbf{r}},\tilde{t}^*)\right] & (\alpha = 1,2,3,4) \\ \eta\left[(1-n_2)\tilde{f}_\alpha^c(\tilde{\mathbf{r}},\tilde{t}^*) + n_2\tilde{f}_{\bar{\alpha}}^c(\tilde{\mathbf{r}},\tilde{t}^*)\right] & (\alpha = 5,6,7,8) \end{cases} \tag{7}$$

Substituting Eq. (6) into Eq. (7), iteration scheme for our new GLBM reads, If $\alpha = 1,2,3,4$

$$\begin{aligned} \frac{1}{\eta}\tilde{f}_\alpha(\tilde{\mathbf{r}} + \mathbf{e}_\alpha, \tilde{t}+1) &= \left(1 - \frac{1}{\tilde{\tau}^*}\right)\left[(1-n_1)\tilde{f}_\alpha(\tilde{\mathbf{r}},\tilde{t}) + n_1\tilde{f}_{\bar{\alpha}}(\tilde{\mathbf{r}},\tilde{t})\right] \\ &+ \left[\frac{\tilde{\rho}}{\tilde{\tau}^*} + \frac{\tilde{\mathbf{F}}\cdot(\mathbf{e}_\alpha - \tilde{\mathbf{u}})}{\tilde{\theta}}\right]\Gamma_\alpha(\tilde{\mathbf{u}}) - n_1\left\{\left[\frac{\tilde{\rho}}{\tilde{\tau}^*} + \frac{\tilde{\mathbf{F}}\cdot(\mathbf{e}_\alpha - \tilde{\mathbf{u}})}{\tilde{\theta}}\right]\Gamma_\alpha(\tilde{\mathbf{u}}) - \left[\frac{\tilde{\rho}}{\tilde{\tau}^*} + \frac{\tilde{\mathbf{F}}\cdot(\mathbf{e}_{\bar{\alpha}} - \tilde{\mathbf{u}})}{\tilde{\theta}}\right]\Gamma_{\bar{\alpha}}(\tilde{\mathbf{u}})\right\} \end{aligned} \tag{8}$$

If $\alpha = 5,6,7,8$

$$\frac{1}{\eta}\tilde{f}_\alpha\left(\tilde{\mathbf{r}}+\mathbf{e}_\alpha,\tilde{t}+1\right)=\left(1-\frac{1}{\tilde{\tau}^*}\right)\left[\left(1-n_2\right)\tilde{f}_\alpha\left(\tilde{\mathbf{r}},\tilde{t}\right)+n_2\tilde{f}_{\bar{\alpha}}\left(\tilde{\mathbf{r}},\tilde{t}\right)\right]$$
$$+\left[\frac{\tilde{\rho}}{\tilde{\tau}^*}+\frac{\tilde{\mathbf{F}}\cdot\left(\mathbf{e}_\alpha-\tilde{\mathbf{u}}\right)}{\tilde{\theta}}\right]\Gamma_\alpha\left(\tilde{\mathbf{u}}\right)-n_2\left\{\left[\frac{\tilde{\rho}}{\tilde{\tau}^*}+\frac{\tilde{\mathbf{F}}\cdot\left(\mathbf{e}_\alpha-\tilde{\mathbf{u}}\right)}{\tilde{\theta}}\right]\Gamma_\alpha\left(\tilde{\mathbf{u}}\right)-\left[\frac{\tilde{\rho}}{\tilde{\tau}^*}+\frac{\tilde{\mathbf{F}}\cdot\left(\mathbf{e}_{\bar{\alpha}}-\tilde{\mathbf{u}}\right)}{\tilde{\theta}}\right]\Gamma_{\bar{\alpha}}\left(\tilde{\mathbf{u}}\right)\right\} \tag{9}$$

The momentum equation can be expressed as

$$\sum_\alpha \tilde{f}_\alpha\mathbf{e}_\alpha=\sum_\alpha \tilde{f}_\alpha^{eq}\mathbf{e}_\alpha=\tilde{\rho}\tilde{\mathbf{u}} \tag{10}$$

or

$$\left(\tilde{f}_1-\tilde{f}_3\right)+\left(\tilde{f}_5+\tilde{f}_8-\tilde{f}_6-\tilde{f}_7\right)=\tilde{\rho}\tilde{u}_x \tag{11}$$

$$\left(\tilde{f}_2-\tilde{f}_4\right)+\left(\tilde{f}_5+\tilde{f}_6-\tilde{f}_7-\tilde{f}_8\right)=\tilde{\rho}\tilde{u}_y \tag{12}$$

We investigate a steady state flow along x-axis, steady state condition reads

$$\frac{\partial(\ )}{\partial\tilde{t}}=0,\quad \frac{\partial(\ )}{\partial\tilde{x}}=0,\quad \tilde{\rho}=const,\quad \tilde{\theta}=const,\quad \tilde{u}_{\tilde{y}}=const=0,\quad \tilde{u}_x=\tilde{u}_x\left(\tilde{y}\right) \tag{13}$$

Assume $\tilde{\mathbf{F}}=\left\{\tilde{F}_x,0\right\}=\tilde{F}_x\mathbf{e}_1$ and $\tilde{\mathbf{u}}=\left\{\tilde{u}_x,0\right\}=\tilde{u}_x\mathbf{e}_1$, thus

$$\Gamma_\alpha\left(\tilde{\mathbf{u}}\right)-\Gamma_{\bar{\alpha}}\left(\tilde{\mathbf{u}}\right)=2w_\alpha\frac{\mathbf{e}_\alpha\cdot\tilde{\mathbf{u}}}{\tilde{\theta}}=6w_\alpha\left(\mathbf{e}_\alpha\cdot\mathbf{e}_1\right)\tilde{u}_x \tag{14}$$

$$\Gamma_\alpha\left(\tilde{\mathbf{u}}\right)+\Gamma_{\bar{\alpha}}\left(\tilde{\mathbf{u}}\right)=2w_\alpha\left[1+\frac{\left(\mathbf{e}_\alpha\cdot\tilde{\mathbf{u}}\right)^2}{2\tilde{\theta}^2}-\frac{\tilde{\mathbf{u}}^2}{2\tilde{\theta}}\right]=w_\alpha\left\{2+3\tilde{u}_x^2\left[3\left(\mathbf{e}_\alpha\cdot\mathbf{e}_1\right)^2-1\right]\right\} \tag{15}$$

$$\left[\frac{\tilde{\rho}}{\tilde{\tau}^*}+\frac{\tilde{\mathbf{F}}\cdot\left(\mathbf{e}_\alpha-\tilde{\mathbf{u}}\right)}{\tilde{\theta}}\right]\Gamma_\alpha\left(\tilde{\mathbf{u}}\right)-\left[\frac{\tilde{\rho}}{\tilde{\tau}^*}+\frac{\tilde{\mathbf{F}}\cdot\left(\mathbf{e}_{\bar{\alpha}}-\tilde{\mathbf{u}}\right)}{\tilde{\theta}}\right]\Gamma_{\bar{\alpha}}\left(\tilde{\mathbf{u}}\right)$$
$$=\frac{6w_\alpha\left(\mathbf{e}_\alpha\cdot\mathbf{e}_1\right)}{\tilde{\tau}^*}\left(\tilde{\rho}\tilde{u}_x+\tilde{\tau}^*\tilde{F}_x\right)-27w_\alpha\tilde{F}_x\tilde{u}_x^2\left[1-\left(\mathbf{e}_\alpha\cdot\mathbf{e}_1\right)^2\right]\left(\mathbf{e}_\alpha\cdot\mathbf{e}_1\right) \tag{16}$$

Notice that for any direction α, last term of right hand side of Eq. (16) is always 0, thus

$$\left[\frac{\tilde{\rho}}{\tilde{\tau}^*}+\frac{\tilde{\mathbf{F}}\cdot\left(\mathbf{e}_\alpha-\tilde{\mathbf{u}}\right)}{\tilde{\theta}}\right]\Gamma_\alpha\left(\tilde{\mathbf{u}}\right)-\left[\frac{\tilde{\rho}}{\tilde{\tau}^*}+\frac{\tilde{\mathbf{F}}\cdot\left(\mathbf{e}_{\bar{\alpha}}-\tilde{\mathbf{u}}\right)}{\tilde{\theta}}\right]\Gamma_{\bar{\alpha}}\left(\tilde{\mathbf{u}}\right)=\frac{6w_\alpha\left(\mathbf{e}_\alpha\cdot\mathbf{e}_1\right)}{\tilde{\tau}^*}\left(\tilde{\rho}\tilde{u}_x+\tilde{\tau}^*\tilde{F}_x\right) \tag{17}$$

Substituting Eq. (17) into Eq. (8), finally we have, if $\alpha = 1,2,3,4$, iteration scheme reads

$$\begin{aligned}\frac{1}{\eta}\tilde{f}_\alpha\left(\tilde{\mathbf{r}}+\mathbf{e}_\alpha,\tilde{t}+1\right) &= \left(1-\frac{1}{\tilde{\tau}^*}\right)\left[\left(1-n_1\right)\tilde{f}_\alpha\left(\tilde{\mathbf{r}},\tilde{t}\right)+n_1\tilde{f}_{\bar{\alpha}}\left(\tilde{\mathbf{r}},\tilde{t}\right)\right] \\ &+\left[\frac{\tilde{\rho}}{\tilde{\tau}^*}+\frac{\tilde{\mathbf{F}}\cdot\left(\mathbf{e}_\alpha-\tilde{\mathbf{u}}\right)}{\tilde{\theta}}\right]\Gamma_\alpha\left(\tilde{\mathbf{u}}\right)-\frac{2n_1\left(\mathbf{e}_\alpha\cdot\mathbf{e}_1\right)}{3\tilde{\tau}^*}\left(\tilde{\rho}\tilde{u}_x+\tilde{\tau}^*\tilde{F}_x\right)\end{aligned} \tag{18}$$

Substituting Eq. (17) into Eq. (9), finally we have, if $\alpha = 5,6,7,8$, iteration scheme reads

$$\begin{aligned}\frac{1}{\eta}\tilde{f}_\alpha\left(\tilde{\mathbf{r}}+\mathbf{e}_\alpha,\tilde{t}+1\right) &= \left(1-\frac{1}{\tilde{\tau}^*}\right)\left[\left(1-n_2\right)\tilde{f}_\alpha\left(\tilde{\mathbf{r}},\tilde{t}\right)+n_2\tilde{f}_{\bar{\alpha}}\left(\tilde{\mathbf{r}},\tilde{t}\right)\right] \\ &+\left[\frac{\tilde{\rho}}{\tilde{\tau}^*}+\frac{\tilde{\mathbf{F}}\cdot\left(\mathbf{e}_\alpha-\tilde{\mathbf{u}}\right)}{\tilde{\theta}}\right]\Gamma_\alpha\left(\tilde{\mathbf{u}}\right)-\frac{n_2\left(\mathbf{e}_\alpha\cdot\mathbf{e}_1\right)}{6\tilde{\tau}^*}\left(\tilde{\rho}\tilde{u}_x+\tilde{\tau}^*\tilde{F}_x\right)\end{aligned} \tag{19}$$

From Eq. (18) we have

$$\begin{aligned}\frac{1}{\eta}\tilde{f}_1^j &= \left(1-\frac{1}{\tilde{\tau}^*}\right)\left[\left(1-n_1\right)\tilde{f}_1^j+n_1\tilde{f}_3^j\right] \\ &+\left[\frac{\tilde{\rho}}{\tilde{\tau}^*}+\frac{\tilde{\mathbf{F}}\cdot\left(\mathbf{e}_1-\tilde{\mathbf{u}}^j\right)}{\tilde{\theta}}\right]\Gamma_1\left(\mathbf{u}^j\right)-\frac{2n_1}{3\tilde{\tau}^*}\left(\tilde{\rho}\tilde{u}_x^j+\tilde{\tau}^*\tilde{F}_x\right)\end{aligned} \tag{20}$$

$$\begin{aligned}\frac{1}{\eta}\tilde{f}_3^j &= \left(1-\frac{1}{\tilde{\tau}^*}\right)\left[\left(1-n_1\right)\tilde{f}_3^j+n_1\tilde{f}_1^j\right] \\ &+\left[\frac{\tilde{\rho}}{\tilde{\tau}^*}+\frac{\tilde{\mathbf{F}}\cdot\left(\mathbf{e}_3-\tilde{\mathbf{u}}^j\right)}{\tilde{\theta}}\right]\Gamma_3\left(\tilde{\mathbf{u}}^j\right)+\frac{2n_1}{3\tilde{\tau}^*}\left(\tilde{\rho}\tilde{u}_x^j+\tilde{\tau}^*\tilde{F}_x\right)\end{aligned} \tag{21}$$

Eq. (20) - Eq. (21) we have

$$\begin{aligned}\frac{1}{\eta}\left(\tilde{f}_1^j-\tilde{f}_3^j\right) &= \left(1-\frac{1}{\tilde{\tau}^*}\right)\left(1-2n_1\right)\left(\tilde{f}_1^j-\tilde{f}_3^j\right) \\ &+\left[\frac{\tilde{\rho}}{\tilde{\tau}^*}+\frac{\tilde{\mathbf{F}}\cdot\left(\mathbf{e}_1-\tilde{\mathbf{u}}^j\right)}{\tilde{\theta}}\right]\Gamma_1\left(\mathbf{u}^j\right)-\left[\frac{\tilde{\rho}}{\tilde{\tau}^*}+\frac{\tilde{\mathbf{F}}\cdot\left(\mathbf{e}_3-\tilde{\mathbf{u}}^j\right)}{\tilde{\theta}}\right]\Gamma_3\left(\tilde{\mathbf{u}}^j\right) \\ &-\frac{4n_1}{3\tilde{\tau}^*}\left(\tilde{\rho}\tilde{u}_x^j+\tilde{\tau}^*\tilde{F}_x\right)\end{aligned} \tag{22}$$

Substituting Eq. into Eq. (22)

$$\frac{1}{\eta}\left(\tilde{f}_1^j-\tilde{f}_3^j\right)=\left(1-\frac{1}{\tilde{\tau}^*}\right)\left(1-2n_1\right)\left(\tilde{f}_1^j-\tilde{f}_3^j\right)+\frac{2\left(1-2n_1\right)}{3\tilde{\tau}^*}\left(\tilde{\rho}\tilde{u}_x^j+\tilde{\tau}^*\tilde{F}_x\right) \tag{23}$$

$$\left(\tilde{f}_1^{\,j}-\tilde{f}_3^{\,j}\right)=2\frac{\left(1-2n_1\right)\eta}{3\left[\tilde{\tau}^*-\eta_1\left(\tilde{\tau}^*-1\right)\left(1-2n_1\right)\right]}\left(\tilde{\rho}\tilde{u}_x^j+\tilde{\tau}^*\tilde{F}_x\right) \tag{24}$$

Denote

$$\gamma=\frac{\left(1-2n_1\right)\eta}{3\left[\tilde{\tau}^*-\eta\left(\tilde{\tau}^*-1\right)\left(1-2n_1\right)\right]} \tag{25}$$

Eq. (24) can be rewritten as

$$\left(\tilde{f}_1^{\,j}-\tilde{f}_3^{\,j}\right)=2\gamma\left(\tilde{\rho}\tilde{u}_x^j+\tilde{\tau}^*\tilde{F}_x\right) \tag{26}$$

Therefore

$$\left(\tilde{f}_1^{\,j-1}-\tilde{f}_3^{\,j-1}\right)=2\gamma\left(\tilde{\rho}\tilde{u}_x^{j-1}+\tilde{\tau}^*\tilde{F}_x\right) \tag{27}$$

$$\left(\tilde{f}_1^{\,j+1}-\tilde{f}_3^{\,j+1}\right)=2\gamma\left(\tilde{\rho}\tilde{u}_x^{j+1}+\tilde{\tau}^*\tilde{F}_x\right) \tag{28}$$

$$\left(\tilde{f}_1^{\,j-1}-\tilde{f}_3^{\,j-1}\right)+\left(\tilde{f}_1^{\,j+1}-\tilde{f}_3^{\,j+1}\right)=2\gamma\left[\tilde{\rho}\left(\tilde{u}_x^{j-1}+\tilde{u}_x^{j+1}\right)+\tilde{\tau}^*2\tilde{F}_x\right] \tag{29}$$

From Eq. (19)

$$\begin{aligned}\frac{1}{\eta}\tilde{f}_5^{\,j+1}&=\left(1-\frac{1}{\tilde{\tau}^*}\right)\left[\left(1-n_2\right)\tilde{f}_5^{\,j}+n_2\tilde{f}_7^{\,j}\right]\\&+\left[\frac{\tilde{\rho}}{\tilde{\tau}^*}+\frac{\tilde{\mathbf{F}}\cdot\left(\mathbf{e}_5-\tilde{\mathbf{u}}^j\right)}{\tilde{\theta}}\right]\Gamma_5\left(\tilde{\mathbf{u}}^j\right)-\frac{n_2}{6\tilde{\tau}^*}\left(\tilde{\rho}\tilde{u}_x^j+\tilde{\tau}^*\tilde{F}_x\right)\end{aligned} \tag{30}$$

$$\begin{aligned}\frac{1}{\eta}\tilde{f}_6^{\,j+1}&=\left(1-\frac{1}{\tilde{\tau}^*}\right)\left[\left(1-n_2\right)\tilde{f}_6^{\,j}+n_2\tilde{f}_8^{\,j}\right]\\&+\left[\frac{\tilde{\rho}}{\tilde{\tau}^*}+\frac{\tilde{\mathbf{F}}\cdot\left(\mathbf{e}_6-\tilde{\mathbf{u}}^j\right)}{\tilde{\theta}}\right]\Gamma_6\left(\tilde{\mathbf{u}}^j\right)+\frac{n_2}{6\tilde{\tau}^*}\left(\tilde{\rho}\tilde{u}_x^j+\tilde{\tau}^*\tilde{F}_x\right)\end{aligned} \tag{31}$$

$$\begin{aligned}\frac{1}{\eta}\tilde{f}_7^{\,j-1}&=\left(1-\frac{1}{\tilde{\tau}^*}\right)\left[\left(1-n_2\right)\tilde{f}_7^{\,j}+n_2\tilde{f}_5^{\,j}\right]\\&+\left[\frac{\tilde{\rho}}{\tilde{\tau}^*}+\frac{\tilde{\mathbf{F}}\cdot\left(\mathbf{e}_7-\mathbf{u}^j\right)}{\tilde{\theta}}\right]\Gamma_7\left(\tilde{\mathbf{u}}^j\right)+\frac{n_2}{6\tilde{\tau}^*}\left(\tilde{\rho}\tilde{u}_x^j+\tilde{\tau}^*\tilde{F}_x\right)\end{aligned} \tag{32}$$

$$\frac{1}{\eta}\tilde{f}_8^{\,j-1}=\left(1-\frac{1}{\tilde{\tau}^*}\right)\left[\left(1-n_2\right)\tilde{f}_8^{\,j}+n_2\tilde{f}_6^{\,j}\right]$$
$$+\left[\frac{\tilde{\rho}}{\tilde{\tau}^*}+\frac{\tilde{\mathbf{F}}\cdot\left(\mathbf{e}_8-\mathbf{u}^j\right)}{\tilde{\theta}}\right]\Gamma_8\left(\tilde{\mathbf{u}}^j\right)-\frac{n_2}{6\tilde{\tau}^*}\left(\tilde{\rho}\tilde{u}_x^j+\tilde{\tau}^*\tilde{F}_x\right) \tag{33}$$

Eq. (30) – Eq. (33) maybe rewritten as

$$\frac{1}{\eta}\tilde{f}_5^{\,j}=\left(1-\frac{1}{\tilde{\tau}^*}\right)\left[\left(1-n_2\right)\tilde{f}_5^{\,j-1}+n_2\tilde{f}_7^{\,j-1}\right]$$
$$+\left[\frac{\tilde{\rho}}{\tilde{\tau}^*}+\frac{\tilde{\mathbf{F}}\cdot\left(\mathbf{e}_5-\tilde{\mathbf{u}}^{j-1}\right)}{\tilde{\theta}}\right]\Gamma_5\left(\tilde{\mathbf{u}}^{j-1}\right)-\frac{n_2}{6\tilde{\tau}^*}\left(\tilde{\rho}\tilde{u}_x^{j-1}+\tilde{\tau}^*\tilde{F}_x\right) \tag{34}$$

$$\frac{1}{\eta}\tilde{f}_6^{\,j}=\left(1-\frac{1}{\tilde{\tau}^*}\right)\left[\left(1-n_2\right)\tilde{f}_6^{\,j-1}+n_2\tilde{f}_8^{\,j-1}\right]$$
$$+\left[\frac{\tilde{\rho}}{\tilde{\tau}^*}+\frac{\tilde{\mathbf{F}}\cdot\left(\mathbf{e}_6-\tilde{\mathbf{u}}^{j-1}\right)}{\tilde{\theta}}\right]\Gamma_6\left(\tilde{\mathbf{u}}^{j-1}\right)+\frac{n_2}{6\tilde{\tau}^*}\left(\tilde{\rho}\tilde{u}_x^{j-1}+\tilde{\tau}^*\tilde{F}_x\right) \tag{35}$$

$$\frac{1}{\eta}f_7^{\,j}=\left(1-\frac{1}{\tilde{\tau}^*}\right)\left[\left(1-n_2\right)\tilde{f}_7^{\,j+1}+n_2\tilde{f}_5^{\,j+1}\right]$$
$$+\left[\frac{\tilde{\rho}}{\tilde{\tau}^*}+\frac{\tilde{\mathbf{F}}\cdot\left(\mathbf{e}_7-\mathbf{u}^{j+1}\right)}{\tilde{\theta}}\right]\Gamma_7\left(\tilde{\mathbf{u}}^{j+1}\right)+\frac{n_2}{6\tilde{\tau}^*}\left(\tilde{\rho}\tilde{u}_x^{j+1}+\tilde{\tau}^*\tilde{F}_x\right) \tag{36}$$

$$\frac{1}{\eta}\tilde{f}_8^{\,j}=\left(1-\frac{1}{\tilde{\tau}^*}\right)\left[\left(1-n_s\right)_2\tilde{f}_8^{\,j+1}+n_2\tilde{f}_6^{\,j+1}\right]$$
$$+\left[\frac{\tilde{\rho}}{\tilde{\tau}^*}+\frac{\tilde{\mathbf{F}}\cdot\left(\mathbf{e}_8-\mathbf{u}^{j+1}\right)}{\tilde{\theta}}\right]\Gamma_8\left(\tilde{\mathbf{u}}^{j+1}\right)-\frac{n_2}{6\tilde{\tau}^*}\left(\tilde{\rho}\tilde{u}_x^{j+1}+\tilde{\tau}^*\tilde{F}_x\right) \tag{37}$$

From Eq. (11)

$$\left(\tilde{f}_5^{\,j-1}-\tilde{f}_6^{\,j-1}\right)+\left(\tilde{f}_8^{\,j-1}-\tilde{f}_7^{\,j-1}\right)=\tilde{\rho}\tilde{u}_x^{j-1}-\left(\tilde{f}_1^{\,j-1}-\tilde{f}_3^{\,j-1}\right) \tag{38}$$

$$\left(\tilde{f}_5^{\,j}-\tilde{f}_6^{\,j}\right)+\left(\tilde{f}_8^{\,j}-\tilde{f}_7^{\,j}\right)=\tilde{\rho}\tilde{u}_x^{j}-\left(\tilde{f}_1^{\,j}-\tilde{f}_3^{\,j}\right) \tag{39}$$

$$\left(\tilde{f}_5^{\,j+1}-\tilde{f}_6^{\,j+1}\right)+\left(\tilde{f}_8^{\,j+1}-\tilde{f}_7^{\,j+1}\right)=\tilde{\rho}\tilde{u}_x^{j+1}-\left(\tilde{f}_1^{\,j+1}-\tilde{f}_3^{\,j+1}\right) \tag{40}$$

From Eq. (34) - Eq. (35) we have

$$\frac{1}{\eta}\left(\tilde{f}_5^{\,j}-\tilde{f}_6^{\,j}\right)=\left(1-\frac{1}{\tilde{\tau}^*}\right)\left[\left(1-n_2\right)\left(\tilde{f}_5^{\,j-1}-\tilde{f}_6^{\,j-1}\right)-n_2\left(\tilde{f}_8^{\,j-1}-\tilde{f}_7^{\,j-1}\right)\right]$$
$$+\left[\frac{\tilde{\rho}}{\tilde{\tau}^*}+\frac{\tilde{\mathbf{F}}\cdot\left(\mathbf{e}_5-\tilde{\mathbf{u}}^{j-1}\right)}{\tilde{\theta}}\right]\Gamma_5\left(\tilde{\mathbf{u}}^{j-1}\right)-\left[\frac{\tilde{\rho}}{\tilde{\tau}^*}+\frac{\tilde{\mathbf{F}}\cdot\left(\mathbf{e}_6-\tilde{\mathbf{u}}^{j-1}\right)}{\tilde{\theta}}\right]\Gamma_6\left(\tilde{\mathbf{u}}^{j-1}\right) \tag{41}$$
$$-\frac{n_2}{3\tilde{\tau}^*}\left(\tilde{\rho}\tilde{u}_x^{j-1}+\tilde{\tau}^*\tilde{F}_x\right)$$

$$\frac{1}{\eta}\left(\tilde{f}_5^{\,j}-\tilde{f}_6^{\,j}\right)=\left(1-\frac{1}{\tilde{\tau}^*}\right)\left(\tilde{f}_5^{\,j-1}-\tilde{f}_6^{\,j-1}\right)-\left(1-\frac{1}{\tilde{\tau}^*}\right)n_2\left[\left(\tilde{f}_5^{\,j-1}-\tilde{f}_6^{\,j-1}\right)+\left(\tilde{f}_8^{\,j-1}-\tilde{f}_7^{\,j-1}\right)\right]$$
$$-\frac{n_2}{3\tilde{\tau}^*}\left(\tilde{\rho}\tilde{u}_x^{j-1}+\tilde{\tau}^*\tilde{F}_x\right)+\frac{\tilde{\rho}}{\tilde{\tau}^*}\left[\Gamma_5\left(\tilde{\mathbf{u}}^{j-1}\right)-\Gamma_6\left(\tilde{\mathbf{u}}^{j-1}\right)\right] \tag{42}$$
$$+3\tilde{\mathbf{F}}\cdot\left[\left(\mathbf{e}_5-\tilde{\mathbf{u}}^{j-1}\right)\Gamma_5\left(\tilde{\mathbf{u}}^{j-1}\right)-\left(\mathbf{e}_6-\tilde{\mathbf{u}}^{j-1}\right)\Gamma_6\left(\tilde{\mathbf{u}}^{j-1}\right)\right]$$

$$\frac{1}{\eta}\left(\tilde{f}_5^{\,j}-\tilde{f}_6^{\,j}\right)=\left(1-\frac{1}{\tilde{\tau}^*}\right)\left(\tilde{f}_5^{\,j-1}-\tilde{f}_6^{\,j-1}\right)-\left(1-\frac{1}{\tilde{\tau}^*}\right)n_2\left[\left(\tilde{f}_5^{\,j-1}-\tilde{f}_6^{\,j-1}\right)+\left(\tilde{f}_8^{\,j-1}-\tilde{f}_7^{\,j-1}\right)\right]$$
$$-\frac{n_2}{3\tilde{\tau}^*}\left(\tilde{\rho}\tilde{u}_x^{j-1}+\tilde{\tau}^*\tilde{F}_x\right)+\frac{1}{6\tilde{\tau}^*}\left(\tilde{\rho}\tilde{u}_x^{j-1}+\tilde{\tau}^*\tilde{F}_x\right) \tag{43}$$

$$\frac{1}{\eta}\left(\tilde{f}_5^{\,j}-\tilde{f}_6^{\,j}\right)=\left(1-\frac{1}{\tilde{\tau}^*}\right)\left(\tilde{f}_5^{\,j-1}-\tilde{f}_6^{\,j-1}\right)-\left(1-\frac{1}{\tilde{\tau}^*}\right)n_2\left[\left(\tilde{f}_5^{\,j-1}-\tilde{f}_6^{\,j-1}\right)+\left(\tilde{f}_8^{\,j-1}-\tilde{f}_7^{\,j-1}\right)\right]$$
$$+\frac{\left(1-2n_2\right)}{6\tilde{\tau}^*}\left(\tilde{\rho}\tilde{u}_x^{j-1}+\tilde{\tau}^*\tilde{F}_x\right) \tag{44}$$

Substituting Eq. (38) into Eq. (44)

$$\frac{1}{\eta}\left(\tilde{f}_5^{\,j}-\tilde{f}_6^{\,j}\right)=\left(1-\frac{1}{\tilde{\tau}^*}\right)\left(\tilde{f}_5^{\,j-1}-\tilde{f}_6^{\,j-1}\right)$$
$$-\left(1-\frac{1}{\tilde{\tau}^*}\right)n_2\left[\tilde{\rho}\tilde{u}_x^{j-1}-\left(\tilde{f}_1^{\,j-1}-\tilde{f}_3^{\,j-1}\right)\right]+\frac{\left(1-2n_2\right)}{6\tilde{\tau}^*}\left(\tilde{\rho}\tilde{u}_x^{j-1}+\tilde{\tau}^*\tilde{F}_x\right) \tag{45}$$

$$\frac{1}{\eta}\left(\tilde{f}_5^{\,j+1}-\tilde{f}_6^{\,j+1}\right)=\left(1-\frac{1}{\tilde{\tau}^*}\right)\left(\tilde{f}_5^{\,j}-\tilde{f}_6^{\,j}\right)$$
$$-\left(1-\frac{1}{\tilde{\tau}^*}\right)n_2\left[\tilde{\rho}\tilde{u}_x^{j}-\left(\tilde{f}_1^{\,j}-\tilde{f}_3^{\,j}\right)\right]+\frac{\left(1-2n_2\right)}{6\tilde{\tau}^*}\left(\tilde{\rho}\tilde{u}_x^{j}+\tilde{\tau}^*\tilde{F}_x\right) \tag{46}$$

From Eq. (45)

$$\left(1-\frac{1}{\tilde{\tau}^*}\right)\left(\tilde{f}_5^{\,j-1}-\tilde{f}_6^{\,j-1}\right)=\frac{1}{\eta}\left(\tilde{f}_5^{\,j}-\tilde{f}_6^{\,j}\right)$$
$$+\left(1-\frac{1}{\tilde{\tau}^*}\right)n_2\left[\tilde{\rho}\tilde{u}_x^{j-1}-\left(\tilde{f}_1^{\,j-1}-\tilde{f}_3^{\,j-1}\right)\right]-\frac{\left(1-2n_2\right)}{6\tilde{\tau}^*}\left(\tilde{\rho}\tilde{u}_x^{j-1}+\tilde{\tau}^*\tilde{F}_x\right) \tag{47}$$

If

$$\tilde{\tau}^* \neq 1 \tag{48}$$

$$\begin{aligned}\left(\tilde{f}_5^{j-1} - \tilde{f}_6^{j-1}\right) &= \frac{\tilde{\tau}^*}{\eta\left(\tilde{\tau}^* - 1\right)}\left(\tilde{f}_5^{j} - \tilde{f}_6^{j}\right) \\ &+ n_2\left[\tilde{\rho}\tilde{u}_x^{j-1} - \left(\tilde{f}_1^{j-1} - \tilde{f}_3^{j-1}\right)\right] - \frac{\left(1-2n_2\right)}{6\left(\tilde{\tau}^* - 1\right)}\left(\tilde{\rho}\tilde{u}_x^{j-1} + \tilde{\tau}^*\tilde{F}_x\right)\end{aligned} \tag{49}$$

Eq. (37) - (36) we have

$$\begin{aligned}\frac{1}{\eta}\left(\tilde{f}_8^{j} - \tilde{f}_7^{j}\right) &= \left(1 - \frac{1}{\tilde{\tau}^*}\right)\left[\left(1-n_2\right)\left(\tilde{f}_8^{j+1} - \tilde{f}_7^{j+1}\right) - n_2\left(\tilde{f}_5^{j+1} - \tilde{f}_6^{j+1}\right)\right] \\ &- \frac{n_2}{3\tilde{\tau}^*}\left(\tilde{\rho}\tilde{u}_x^{j+1} + \tilde{\tau}^*\tilde{F}_x\right) \\ &+ \left[\frac{\tilde{\rho}}{\tilde{\tau}^*} + \frac{\tilde{\mathbf{F}}\cdot\left(\mathbf{e}_8 - \mathbf{u}^{j+1}\right)}{\tilde{\theta}}\right]\Gamma_8\left(\tilde{\mathbf{u}}^{j+1}\right) - \left[\frac{\tilde{\rho}}{\tilde{\tau}^*} + \frac{\tilde{\mathbf{F}}\cdot\left(\mathbf{e}_7 - \mathbf{u}^{j+1}\right)}{\tilde{\theta}}\right]\Gamma_7\left(\tilde{\mathbf{u}}^{j+1}\right)\end{aligned} \tag{50}$$

$$\begin{aligned}\frac{1}{\eta}\left(\tilde{f}_8^{j} - \tilde{f}_7^{j}\right) &= \left(1 - \frac{1}{\tilde{\tau}^*}\right)\left(\tilde{f}_8^{j+1} - \tilde{f}_7^{j+1}\right) - \left(1 - \frac{1}{\tilde{\tau}^*}\right)n_2\left[\left(\tilde{f}_8^{j+1} - \tilde{f}_7^{j+1}\right) + \left(\tilde{f}_5^{j+1} - \tilde{f}_6^{j+1}\right)\right] \\ &+ \frac{\left(1-2n_2\right)}{6\tilde{\tau}^*}\left(\tilde{\rho}\tilde{u}_x^{j+1} + \tilde{\tau}^*\tilde{F}_x\right)\end{aligned} \tag{51}$$

Substituting Eq. (40) into Eq. (51)

$$\begin{aligned}\frac{1}{\eta}\left(\tilde{f}_8^{j} - \tilde{f}_7^{j}\right) &= \left(1 - \frac{1}{\tilde{\tau}^*}\right)\left(\tilde{f}_8^{j+1} - \tilde{f}_7^{j+1}\right) - \left(1 - \frac{1}{\tilde{\tau}^*}\right)n_2\left[\tilde{\rho}\tilde{u}_x^{j+1} - \left(\tilde{f}_1^{j+1} - \tilde{f}_3^{j+1}\right)\right] \\ &+ \frac{\left(1-2n_2\right)}{6\tilde{\tau}^*}\left(\tilde{\rho}\tilde{u}_x^{j+1} + \tilde{\tau}^*\tilde{F}_x\right)\end{aligned} \tag{52}$$

Thus

$$\begin{aligned}\frac{1}{\eta}\left(\tilde{f}_8^{j-1} - \tilde{f}_7^{j-1}\right) &= \left(1 - \frac{1}{\tilde{\tau}^*}\right)\left(\tilde{f}_8^{j} - \tilde{f}_7^{j}\right) - \left(1 - \frac{1}{\tilde{\tau}^*}\right)n_2\left[\tilde{\rho}\tilde{u}_x^{j} - \left(\tilde{f}_1^{j} - \tilde{f}_3^{j}\right)\right] \\ &+ \frac{\left(1-2n_2\right)}{6\tilde{\tau}^*}\left(\tilde{\rho}\tilde{u}_x^{j} + \tilde{\tau}^*\tilde{F}_x\right)\end{aligned} \tag{53}$$

From Eq. (52)

$$\left(1-\frac{1}{\tilde{\tau}^*}\right)\left(\tilde{f}_8^{j+1}-\tilde{f}_7^{j+1}\right)=\frac{1}{\eta}\left(\tilde{f}_8^{j}-\tilde{f}_7^{j}\right)+\left(1-\frac{1}{\tilde{\tau}^*}\right)n_2\left[\tilde{\rho}\tilde{u}_x^{j+1}-\left(\tilde{f}_1^{j+1}-\tilde{f}_3^{j+1}\right)\right] \\ -\frac{\left(1-2n_2\right)}{6\tilde{\tau}^*}\left(\tilde{\rho}\tilde{u}_x^{j+1}+\tilde{\tau}^*\tilde{F}_x\right) \tag{54}$$

if

$$\tilde{\tau}^* \neq 1 \tag{55}$$

$$\left(\tilde{f}_8^{j+1}-\tilde{f}_7^{j+1}\right)=\frac{\tilde{\tau}^*}{\eta\left(\tilde{\tau}^*-1\right)}\left(\tilde{f}_8^{j}-\tilde{f}_7^{j}\right)+n_2\left[\tilde{\rho}\tilde{u}_x^{j+1}-\left(\tilde{f}_1^{j+1}-\tilde{f}_3^{j+1}\right)\right] \\ -\frac{\left(1-2n_2\right)}{6\left(\tilde{\tau}^*-1\right)}\left(\tilde{\rho}\tilde{u}_x^{j+1}+\tilde{\tau}^*\tilde{F}_x\right) \tag{56}$$

From Eq. (46) and Eq. (49)

$$\left(\tilde{f}_5^{j-1}-\tilde{f}_6^{j-1}\right)+\left(\tilde{f}_5^{j+1}-\tilde{f}_6^{j+1}\right)=\left[\frac{\tilde{\tau}^*}{\eta\left(\tilde{\tau}^*-1\right)}+\frac{\eta\left(\tilde{\tau}^*-1\right)}{\tilde{\tau}^*}\right]\left(\tilde{f}_5^{j}-\tilde{f}_6^{j}\right) \\ +n_2\left[\tilde{\rho}\tilde{u}_x^{j-1}-\left(\tilde{f}_1^{j-1}-\tilde{f}_3^{j-1}\right)\right]-\eta\left(1-\frac{1}{\tilde{\tau}^*}\right)n_2\left[\tilde{\rho}\tilde{u}_x^{j}-\left(\tilde{f}_1^{j}-\tilde{f}_3^{j}\right)\right] \\ -\frac{\left(1-2n_2\right)}{6\left(\tilde{\tau}^*-1\right)}\left(\tilde{\rho}\tilde{u}_x^{j-1}+\tilde{\tau}^*\tilde{F}_x\right)+\frac{\eta\left(1-2n_2\right)}{6\tilde{\tau}^*}\left(\tilde{\rho}\tilde{u}_x^{j}+\tilde{\tau}^*\tilde{F}_x\right) \tag{57}$$

From Eq. (53) and Eq. (56)

$$\left(\tilde{f}_8^{j-1}-\tilde{f}_7^{j-1}\right)+\left(\tilde{f}_8^{j+1}-\tilde{f}_7^{j+1}\right)=\left[\frac{\tilde{\tau}^*}{\eta\left(\tilde{\tau}^*-1\right)}+\frac{\eta\left(\tilde{\tau}^*-1\right)}{\tilde{\tau}^*}\right]\left(\tilde{f}_8^{j}-\tilde{f}_7^{j}\right) \\ +n_2\left[\tilde{\rho}\tilde{u}_x^{j+1}-\left(\tilde{f}_1^{j+1}-\tilde{f}_3^{j+1}\right)\right]-\eta\left(1-\frac{1}{\tilde{\tau}^*}\right)n_2\left[\tilde{\rho}\tilde{u}_x^{j}-\left(\tilde{f}_1^{j}-\tilde{f}_3^{j}\right)\right] \\ -\frac{\left(1-2n_2\right)}{6\left(\tilde{\tau}^*-1\right)}\left(\tilde{\rho}\tilde{u}_x^{j+1}+\tilde{\tau}^*\tilde{F}_x\right)+\frac{\eta\left(1-2n_2\right)}{6\tilde{\tau}^*}\left(\tilde{\rho}\tilde{u}_x^{j}+\tilde{\tau}^*\tilde{F}_x\right) \tag{58}$$

From Eq. (38) and Eq. (40)

$$\left[\left(\tilde{f}_5^{j-1}-\tilde{f}_6^{j-1}\right)+\left(\tilde{f}_5^{j+1}-\tilde{f}_6^{j+1}\right)\right]+\left[\left(\tilde{f}_8^{j-1}-\tilde{f}_7^{j-1}\right)+\left(\tilde{f}_8^{j+1}-\tilde{f}_7^{j+1}\right)\right] \\ =\tilde{\rho}\left(\tilde{u}_x^{j-1}+\tilde{u}_x^{j+1}\right)-\left[\left(\tilde{f}_1^{j-1}-\tilde{f}_3^{j-1}\right)+\left(\tilde{f}_1^{j+1}-\tilde{f}_3^{j+1}\right)\right] \tag{59}$$

Substituting Eq. (57) and Eq. (58) into Eq. (59)

$$\left[\frac{\tilde{\tau}^*}{\eta\left(\tilde{\tau}^*-1\right)}+\frac{\eta\left(\tilde{\tau}^*-1\right)}{\tilde{\tau}^*}\right]\left[\left(\tilde{f}_5^{\,j}-\tilde{f}_6^{\,j}\right)+\left(\tilde{f}_8^{\,j}-\tilde{f}_7^{\,j}\right)\right]$$
$$+n_2\left\{\tilde{\rho}\left(\tilde{u}_x^{j-1}+\tilde{u}_x^{j+1}\right)-\left[\left(\tilde{f}_1^{\,j-1}-\tilde{f}_3^{\,j-1}\right)+\left(\tilde{f}_1^{\,j+1}-\tilde{f}_3^{\,j+1}\right)\right]\right\}$$
$$-2\eta\left(1-\frac{1}{\tilde{\tau}^*}\right)n_2\left[\tilde{\rho}\tilde{u}_x^j-\left(\tilde{f}_1^{\,j}-\tilde{f}_3^{\,j}\right)\right] \tag{60}$$
$$+\frac{\eta\left(1-2n_2\right)}{3\tilde{\tau}^*}\left(\tilde{\rho}\tilde{u}_x^j+\tilde{\tau}^*F_x\right)-\frac{\left(1-2n_2\right)}{6\left(\tilde{\tau}^*-1\right)}\left[\tilde{\rho}\left(\tilde{u}_x^{j-1}+\tilde{u}_x^{j+1}\right)+\tilde{\tau}^*2\tilde{F}_x\right]$$
$$=\tilde{\rho}\left(\tilde{u}_x^{j-1}+\tilde{u}_x^{j+1}\right)-\left[\left(\tilde{f}_1^{\,j-1}-\tilde{f}_3^{\,j-1}\right)+\left(\tilde{f}_1^{\,j+1}-\tilde{f}_3^{\,j+1}\right)\right]$$

Substituting Eq. (39) into Eq. (60)

$$\left[\frac{\tilde{\tau}^*}{\eta\left(\tilde{\tau}^*-1\right)}+\frac{\eta\left(\tilde{\tau}^*-1\right)\left(1-2n_2\right)}{\tilde{\tau}^*}\right]\left[\tilde{\rho}\tilde{u}_x^j-\left(\tilde{f}_1^{\,j}-\tilde{f}_3^{\,j}\right)\right]$$
$$+\frac{\eta\left(1-2n_2\right)}{3\tilde{\tau}^*}\left(\tilde{\rho}\tilde{u}_x^j+\tilde{\tau}^*\tilde{F}_x\right)-\frac{\left(1-2n_s\right)_2}{6\left(\tilde{\tau}^*-1\right)}\left[\tilde{\rho}\left(\tilde{u}_x^{j-1}+\tilde{u}_x^{j+1}\right)+\tilde{\tau}^*2\tilde{F}_x\right] \tag{61}$$
$$=\left(1-n_2\right)\left\{\tilde{\rho}\left(\tilde{u}_x^{j-1}+\tilde{u}_x^{j+1}\right)-\left[\left(\tilde{f}_1^{\,j-1}-\tilde{f}_3^{\,j-1}\right)+\left(\tilde{f}_1^{\,j+1}-\tilde{f}_3^{\,j+1}\right)\right]\right\}$$

Substituting Eq. (26) - Eq. (29) into Eq. (61) we have

$$\left[\frac{\tilde{\tau}^*}{\eta\left(\tilde{\tau}^*-1\right)}+\frac{\eta\left(\tilde{\tau}^*-1\right)\left(1-2n_2\right)}{\tilde{\tau}^*}\right]\left[\tilde{\rho}\tilde{u}_x^j-2\gamma\left(\tilde{\rho}\tilde{u}_x^j+\tilde{\tau}^*\tilde{F}_x\right)\right]$$
$$+\frac{\eta\left(1-2n_2\right)}{3\tilde{\tau}^*}\left(\tilde{\rho}\tilde{u}_x^j+\tilde{\tau}^*\tilde{F}_x\right)-\frac{\left(1-2n_2\right)}{6\left(\tilde{\tau}^*-1\right)}\left[\tilde{\rho}\left(\tilde{u}_x^{j-1}+\tilde{u}_x^{j+1}\right)+\tilde{\tau}^*2\tilde{F}_x\right] \tag{62}$$
$$=\left(1-n_2\right)\left\{\tilde{\rho}\left(\tilde{u}_x^{j-1}+\tilde{u}_x^{j+1}\right)-2\gamma\left[\tilde{\rho}\left(\tilde{u}_x^{j-1}+\tilde{u}_x^{j+1}\right)+\tilde{\tau}^*2\tilde{F}_x\right]\right\}$$

$$\left[\frac{\tilde{\tau}^*}{\eta\left(\tilde{\tau}^*-1\right)}+\frac{\eta\left(\tilde{\tau}^*-1\right)\left(1-2n_2\right)}{\tilde{\tau}^*}\right]\left(\tilde{\rho}\tilde{u}_x^j\right)$$
$$+\left\{\frac{\eta\left(1-2n_2\right)}{3\tilde{\tau}^*}-2\gamma\left[\frac{\tilde{\tau}^*}{\eta\left(\tilde{\tau}^*-1\right)}+\frac{\eta\left(\tilde{\tau}^*-1\right)\left(1-2n_2\right)}{\tilde{\tau}^*}\right]\right\}\left(\tilde{\rho}\tilde{u}_x^j+\tilde{\tau}^*\tilde{F}_x\right)$$
$$+\left[\left(2\gamma-1\right)\left(1-n_2\right)-\frac{\left(1-2n_2\right)}{6\left(\tilde{\tau}^*-1\right)}\right]\left[\tilde{\rho}\left(\tilde{u}_x^{j-1}+\tilde{u}_x^{j+1}\right)\right] \tag{63}$$
$$+\left[2\gamma\left(1-n_2\right)-\frac{\left(1-2n_2\right)}{6\left(\tilde{\tau}^*-1\right)}\right]\tilde{\tau}^*2\tilde{F}_x=0$$

$$
\begin{aligned}
&\left[\frac{\tilde{\tau}^*}{\eta\left(\tilde{\tau}^*-1\right)}+\frac{\eta\left(\tilde{\tau}^*-1\right)\left(1-2n_2\right)}{\tilde{\tau}^*}\right]\left(\tilde{\rho}\tilde{u}_x^j\right)\\
&+\left\{\frac{\eta\left(1-2n_2\right)}{3\tilde{\tau}^*}-2\gamma\left[\frac{\tilde{\tau}^*}{\eta\left(\tilde{\tau}^*-1\right)}+\frac{\eta\left(\tilde{\tau}^*-1\right)\left(1-2n_2\right)}{\tilde{\tau}^*}\right]\right\}\left(\tilde{\rho}\tilde{u}_x^j\right)\\
&+\left\{\frac{\eta\left(1-2n_2\right)}{3\tilde{\tau}^*}-2\gamma\left[\frac{\tilde{\tau}^*}{\eta\left(\tilde{\tau}^*-1\right)}+\frac{\eta\left(\tilde{\tau}^*-1\right)\left(1-2n_2\right)}{\tilde{\tau}^*}\right]\right\}\left(\tilde{\tau}^*\tilde{F}_x\right)\\
&+\left[\left(2\gamma-1\right)\left(1-n_2\right)-\frac{\left(1-2n_2\right)}{6\left(\tilde{\tau}^*-1\right)}\right]\left[\tilde{\rho}\left(\tilde{u}_x^{j-1}+\tilde{u}_x^{j+1}\right)\right]\\
&+\left[2\gamma\left(1-n_2\right)-\frac{\left(1-2n_2\right)}{6\left(\tilde{\tau}^*-1\right)}\right]\tilde{\tau}^*2\tilde{F}_x=0
\end{aligned}
\tag{64}
$$

$$
\begin{aligned}
&\left[\frac{\tilde{\tau}^*}{\eta\left(\tilde{\tau}^*-1\right)}+\frac{\eta\left(\tilde{\tau}^*-1\right)\left(1-2n_2\right)}{\tilde{\tau}^*}\right]\left(\tilde{\rho}\tilde{u}_x^j\right)\\
&+\left\{\frac{\eta\left(1-2n_2\right)}{3\tilde{\tau}^*}-2\gamma\left[\frac{\tilde{\tau}^*}{\eta\left(\tilde{\tau}^*-1\right)}+\frac{\eta\left(\tilde{\tau}^*-1\right)\left(1-2n_2\right)}{\tilde{\tau}^*}\right]\right\}\left(\tilde{\rho}\tilde{u}_x^j\right)\\
&+2\left[\left(2\gamma-1\right)\left(1-n_2\right)-\frac{\left(1-2n_2\right)}{6\left(\tilde{\tau}^*-1\right)}\right]\left(\tilde{\rho}\tilde{u}_x^j\right)\\
&+\left\{\frac{\eta\left(1-2n_2\right)}{3\tilde{\tau}^*}-2\gamma\left[\frac{\tilde{\tau}^*}{\eta\left(\tilde{\tau}^*-1\right)}+\frac{\eta\left(\tilde{\tau}^*-1\right)\left(1-2n_2\right)}{\tilde{\tau}^*}\right]\right\}\left(\tilde{\tau}^*\tilde{F}_x\right)\\
&+2\left[2\gamma\left(1-n_2\right)-\frac{\left(1-2n_2\right)}{6\left(\tilde{\tau}^*-1\right)}\right]\left(\tilde{\tau}^*\tilde{F}_x\right)\\
&+\left[\left(2\gamma-1\right)\left(1-n_2\right)-\frac{\left(1-2n_2\right)}{6\left(\tilde{\tau}^*-1\right)}\right]\left[\tilde{\rho}\left(\tilde{u}_x^{j-1}+\tilde{u}_x^{j+1}-2\tilde{u}_x^j\right)\right]=0
\end{aligned}
\tag{65}
$$

$$
+\left\{\begin{array}{c}\frac{\eta(1-2n_2)}{3\tilde{\tau}^*}-(2\gamma-1)\left[\frac{\tilde{\tau}^*}{\eta(\tilde{\tau}^*-1)}+\frac{\eta(\tilde{\tau}^*-1)(1-2n_2)}{\tilde{\tau}^*}\right] \\ +2\left[(2\gamma-1)(1-n_2)-\frac{(1-2n_2)}{6(\tilde{\tau}^*-1)}\right]\end{array}\right\}\left(\tilde{\rho}\tilde{u}_x^j\right)
$$

$$
+\left\{2\left[2\gamma(1-n_2)-\frac{(1-2n_2)}{6(\tilde{\tau}^*-1)}\right]+\frac{\eta(1-2n_2)}{3\tilde{\tau}^*}-2\gamma\left[\frac{\tilde{\tau}^*}{\eta(\tilde{\tau}^*-1)}+\frac{\eta(\tilde{\tau}^*-1)(1-2n_2)}{\tilde{\tau}^*}\right]\right\}\left(\tilde{\tau}^*\tilde{F}_x\right) \tag{66}
$$

$$
+\left[(2\gamma-1)(1-n_2)-\frac{(1-2n_2)}{6(\tilde{\tau}^*-1)}\right]\left[\tilde{\rho}\left(\tilde{u}_x^{j-1}+\tilde{u}_x^{j+1}-2\tilde{u}_x^j\right)\right]=0
$$

$$
+\left\{\frac{\eta(1-2n_2)}{3\tilde{\tau}^*}-(2\gamma-1)\left[\frac{\tilde{\tau}^*}{\eta(\tilde{\tau}^*-1)}+\frac{\eta(\tilde{\tau}^*-1)(1-2n_2)}{\tilde{\tau}^*}-2(1-n_2)\right]-\frac{(1-2n_2)}{3(\tilde{\tau}^*-1)}\right\}\left(\tilde{\rho}\tilde{u}_x^j\right)
$$

$$
+\left\{\frac{\eta(1-2n_2)}{3\tilde{\tau}^*}-2\gamma\left[\frac{\tilde{\tau}^*}{\eta(\tilde{\tau}^*-1)}+\frac{\eta(\tilde{\tau}^*-1)(1-2n_2)}{\tilde{\tau}^*}-2(1-n_2)\right]-\frac{(1-2n_2)}{3(\tilde{\tau}^*-1)}\right\}\left(\tilde{\tau}^*\tilde{F}_x\right) \tag{67}
$$

$$
+\left[(2\gamma-1)(1-n_2)-\frac{(1-2n_2)}{6(\tilde{\tau}^*-1)}\right]\left[\tilde{\rho}\left(\tilde{u}_x^{j-1}+\tilde{u}_x^{j+1}-2\tilde{u}_x^j\right)\right]=0
$$

Denote

$$
\omega=\frac{\tilde{\tau}^*}{\eta(\tilde{\tau}^*-1)}+\eta\left(1-\frac{1}{\tilde{\tau}^*}\right)(1-2n_2)-2(1-n_2) \tag{68}
$$

Then Eq. (67) can be rewritten as

$$
+\left\{\frac{\eta(1-2n_2)}{3\tilde{\tau}^*}-(2\gamma-1)\omega-\frac{(1-2n_2)}{3(\tilde{\tau}^*-1)}\right\}\left(\tilde{\rho}\tilde{u}_x^j\right)
$$

$$
+\left\{\frac{\eta(1-2n_2)}{3\tilde{\tau}^*}-2\gamma\omega-\frac{(1-2n_2)}{3(\tilde{\tau}^*-1)}\right\}\left(\tilde{\tau}^*\tilde{F}_x\right) \tag{69}
$$

$$
+\left[(2\gamma-1)(1-n_2)-\frac{(1-2n_2)}{6(\tilde{\tau}^*-1)}\right]\left[\tilde{\rho}\left(\tilde{u}_x^{j-1}+\tilde{u}_x^{j+1}-2\tilde{u}_x^j\right)\right]=0
$$

$$
\begin{aligned}
&\left[(1-2\gamma)(1-n_2)+\frac{(1-2n_2)}{6(\tilde{\tau}^*-1)}\right]\tilde{\rho}\left(\tilde{u}_x^{j-1}+\tilde{u}_x^{j+1}-2\tilde{u}_x^j\right) \\
&-\left[\omega(1-2\gamma)+\frac{(1-2n_2)}{3}\left(\frac{\eta}{\tilde{\tau}^*}-\frac{1}{\tilde{\tau}^*-1}\right)\right]\left(\tilde{\rho}\tilde{u}_x^j\right) \\
&+\left[2\gamma\omega-\frac{(1-2n_2)}{3}\left(\frac{\eta}{\tilde{\tau}^*}-\frac{1}{\tilde{\tau}^*-1}\right)\right]\tilde{\tau}^*\left(\tilde{F}_x\right)=0
\end{aligned}
\tag{70}
$$

Denote

$$
\begin{cases}
A=(1-2\gamma)(1-n_2)+\dfrac{(1-2n_2)}{6(\tilde{\tau}^*-1)} \\
B=\omega(1-2\gamma)+\dfrac{(1-2n_2)}{3}\left(\dfrac{\eta}{\tilde{\tau}^*}-\dfrac{1}{\tilde{\tau}^*-1}\right) \\
C=\left[2\gamma\omega-\dfrac{(1-2n_2)}{3}\left(\dfrac{\eta}{\tilde{\tau}^*}-\dfrac{1}{\tilde{\tau}^*-1}\right)\right]\tilde{\tau}^*
\end{cases}
\tag{71}
$$

Then Eq. (70) can be rewritten as

$$
A\left(\tilde{u}_x^{j-1}+\tilde{u}_x^{j+1}-2\tilde{u}_x^j\right)-B\tilde{u}_x^j+C\frac{\tilde{F}_x}{\tilde{\rho}}=0
\tag{72}
$$

$$
\begin{cases}
\omega=\dfrac{\tilde{\tau}^*}{\eta(\tilde{\tau}^*-1)}+\eta\left(1-\dfrac{1}{\tilde{\tau}^*}\right)(1-2n_2)-2(1-n_2) \\
\gamma=\dfrac{(1-2n_1)\eta}{3\left[\tilde{\tau}^*-\eta(\tilde{\tau}^*-1)(1-2n_1)\right]}
\end{cases}
\tag{73}
$$

Extending a Gray Lattice Boltzmann Model for Simulating Fluid Flow in Multi-scale Porous Media

Jiujiang Zhu[a], Jingsheng Ma[b]

[a] *School of Civil Engineering, Wuyi University, China*

[b] *Institute of Petroleum Engineering, Heriot-Watt University, Edinburgh, UK*

Appendix C: Compare GLBM with other model

1. Compare with Brinkman Body Force scheme (BBF)

Our GLBM involves the following 3 steps:

1: Collision step $\tilde{f}_\alpha^c\left(\tilde{\mathbf{r}},\tilde{t}\right)=\tilde{f}_\alpha\left(\tilde{\mathbf{r}},\tilde{t}\right)-\dfrac{\tilde{f}_\alpha\left(\tilde{\mathbf{r}},\tilde{t}\right)-\tilde{f}_\alpha^{eq}\left(\tilde{\mathbf{r}},\tilde{t}\right)}{\tilde{\tau}^*}+\dfrac{\tilde{\mathbf{F}}\cdot\left(\mathbf{e}_\alpha-\tilde{\mathbf{u}}\right)}{\tilde{\theta}}\Gamma_\alpha\left(\tilde{\mathbf{u}}\right)$ (1)

2: Repartition step (combined two repartition steps)

$$\tilde{f}_\alpha^{out}\left(\tilde{\mathbf{r}},\tilde{t}^{**}\right)=\begin{cases}\eta\left[\left(1-n_s\right)\tilde{f}_\alpha^c\left(\tilde{\mathbf{r}},\tilde{t}^*\right)+n_s\tilde{f}_{\bar{\alpha}}^c\left(\tilde{\mathbf{r}},\tilde{t}^*\right)\right] & \text{if } \alpha\neq 0\\ \left(1-\eta\right)\tilde{\rho}+\eta\tilde{f}_0^c & \text{if } \alpha=0\end{cases} \tag{2}$$

3: Streaming

$$\tilde{f}_\alpha\left(\tilde{\mathbf{r}}+\mathbf{e}_\alpha,\tilde{t}+1\right)=\tilde{f}_\alpha^{out}\left(\tilde{\mathbf{r}},\tilde{t}^{**}\right) \tag{3}$$

In order to compare GLBM with the Brinkman Body Force scheme, let assume the external body force consists of two parts:

$$\tilde{\mathbf{F}}\left(\tilde{\mathbf{u}},\tilde{\mathbf{r}},\tilde{t}\right)=\mathbf{G}\left(\tilde{\mathbf{r}},\tilde{t}\right)-k\tilde{\mathbf{u}}\left(\tilde{\mathbf{r}},\tilde{t}\right) \tag{4}$$

The first part $\mathbf{G}$ in Eq. (4), is the conventional external active body force while the second part is the reactive resistant body force by subscale pore media:

$$\tilde{\mathbf{F}}_R=-k\tilde{\mathbf{u}}=-\frac{\phi\nu_f}{\kappa}\tilde{\mathbf{u}} \tag{5}$$

in which

$$k = \frac{\phi \nu_f}{\kappa} \tag{6}$$

and $\kappa, \phi,$ and ν_f are the permeability and porosity of the subscale porous media and fluid viscosity.

In terms of $k, \eta,$ and n_s, two different models correspond to:

(1) Brinkman Body Force (BBF) LBM scheme

$$k = \frac{\phi \nu_f}{\kappa}; \quad \eta = 1; \quad n_s = 0 \tag{7}$$

(2) Gray Lattice Boltzmann (GLBM) model

$$k = 0; \quad \eta \neq 1; \quad n_s \neq 0 \tag{8}$$

For the most general model at

$$k \neq 0; \quad \eta \neq 1; \quad n_s \neq 0 \tag{9}$$

Let define the post streaming momentum (for $\alpha \neq 0$)

$$\mathbf{SM}_\alpha = \tilde{f}_\alpha(\tilde{\mathbf{r}}, \tilde{t})\mathbf{e}_\alpha + \tilde{f}_{\bar{\alpha}}(\tilde{\mathbf{r}}, \tilde{t})\mathbf{e}_{\bar{\alpha}} = \left[\tilde{f}_\alpha(\tilde{\mathbf{r}}, \tilde{t}) - \tilde{f}_{\bar{\alpha}}(\tilde{\mathbf{r}}, \tilde{t})\right]\mathbf{e}_\alpha \tag{10}$$

which is the momentum associated to a pair of opposite directions α and $\bar{\alpha}$ after streaming step.

Let define the post collision and post repartition momentums as:

$$\mathbf{CM}_\alpha = \tilde{f}^c_\alpha(\tilde{\mathbf{r}}, \tilde{t}^*)\mathbf{e}_\alpha + \tilde{f}^c_{\bar{\alpha}}(\tilde{\mathbf{r}}, \tilde{t}^*)\mathbf{e}_{\bar{\alpha}} = \left[\tilde{f}^c_\alpha(\tilde{\mathbf{r}}, \tilde{t}^*) - \tilde{f}^c_{\bar{\alpha}}(\tilde{\mathbf{r}}, \tilde{t}^*)\right]\mathbf{e}_\alpha \tag{11}$$

$$\mathbf{RM}_\alpha = \tilde{f}^{out}_\alpha(\tilde{\mathbf{r}}, \tilde{t}^{**})\mathbf{e}_\alpha + \tilde{f}^{out}_{\bar{\alpha}}(\tilde{\mathbf{r}}, \tilde{t}^{**})\mathbf{e}_{\bar{\alpha}} \tag{12}$$

Since

$$\begin{aligned}
&\tilde{f}_{\alpha}^{out}\left(\tilde{\mathbf{r}},\tilde{t}^{**}\right)\mathbf{e}_{\alpha}+\tilde{f}_{\bar{\alpha}}^{out}\left(\tilde{\mathbf{r}},\tilde{t}^{**}\right)\mathbf{e}_{\bar{\alpha}} \\
&=\eta\left[\left(1-n_s\right)\tilde{f}_{\alpha}^{c}\left(\tilde{\mathbf{r}},\tilde{t}^{*}\right)+n_s\tilde{f}_{\bar{\alpha}}^{c}\left(\tilde{\mathbf{r}},\tilde{t}^{*}\right)\right]\mathbf{e}_{\alpha} \\
&-\eta\left[\left(1-n_s\right)\tilde{f}_{\bar{\alpha}}^{c}\left(\tilde{\mathbf{r}},\tilde{t}^{*}\right)+n_s\tilde{f}_{\alpha}^{c}\left(\tilde{\mathbf{r}},\tilde{t}^{*}\right)\right]\mathbf{e}_{\alpha} \\
&=\eta\left(1-2n_s\right)\left[\tilde{f}_{\alpha}^{c}\left(\tilde{\mathbf{r}},\tilde{t}^{*}\right)-\tilde{f}_{\bar{\alpha}}^{c}\left(\tilde{\mathbf{r}},\tilde{t}^{*}\right)\right]\mathbf{e}_{\alpha}
\end{aligned} \tag{13}$$

Therefore

$$\mathbf{RM}_{\alpha}=\eta\left(1-2n_s\right)\left[\tilde{f}_{\alpha}^{c}\left(\tilde{\mathbf{r}},\tilde{t}^{*}\right)-\tilde{f}_{\bar{\alpha}}^{c}\left(\tilde{\mathbf{r}},\tilde{t}^{*}\right)\right]\mathbf{e}_{\alpha} \tag{14}$$

The momentum loss during the collision can be calculated as:

$$\begin{aligned}
\Delta\mathbf{CM}_{\alpha}&=\mathbf{SM}_{\alpha}-\mathbf{CM}_{\alpha} \\
&=\left[\tilde{f}_{\alpha}\left(\tilde{\mathbf{r}},\tilde{t}\right)-\tilde{f}_{\bar{\alpha}}\left(\tilde{\mathbf{r}},\tilde{t}\right)\right]\mathbf{e}_{\alpha}-\left[\tilde{f}_{\alpha}^{c}\left(\tilde{\mathbf{r}},\tilde{t}^{*}\right)-\tilde{f}_{\bar{\alpha}}^{c}\left(\tilde{\mathbf{r}},\tilde{t}^{*}\right)\right]\mathbf{e}_{\alpha}
\end{aligned} \tag{15}$$

Substituting Eq. (1) into Eq. (15)

$$\begin{aligned}
\Delta\mathbf{CM}_{\alpha}&=\mathbf{SM}_{\alpha}-\mathbf{CM}_{\alpha} \\
&=\left\{\left[\tilde{f}_{\alpha}\left(\tilde{\mathbf{r}},\tilde{t}\right)-\tilde{f}_{\alpha}^{c}\left(\tilde{\mathbf{r}},\tilde{t}^{*}\right)\right]-\left[\tilde{f}_{\bar{\alpha}}\left(\tilde{\mathbf{r}},\tilde{t}\right)-\tilde{f}_{\bar{\alpha}}^{c}\left(\tilde{\mathbf{r}},\tilde{t}^{*}\right)\right]\right\}\mathbf{e}_{\alpha} \\
&=\left\{\begin{array}{l}
\left[\dfrac{\tilde{f}_{\alpha}\left(\tilde{\mathbf{r}},\tilde{t}\right)-\tilde{f}_{\alpha}^{eq}\left(\tilde{\mathbf{r}},\tilde{t}\right)}{\tilde{\tau}^{*}}-\dfrac{\tilde{\mathbf{F}}\cdot\left(\mathbf{e}_{\alpha}-\tilde{\mathbf{u}}\right)}{\tilde{\theta}}\Gamma_{\alpha}\left(\tilde{\mathbf{u}}\right)\right] \\
-\left[\dfrac{\tilde{f}_{\bar{\alpha}}\left(\tilde{\mathbf{r}},\tilde{t}\right)-\tilde{f}_{\bar{\alpha}}^{eq}\left(\tilde{\mathbf{r}},\tilde{t}\right)}{\tilde{\tau}^{*}}-\dfrac{\tilde{\mathbf{F}}\cdot\left(\mathbf{e}_{\bar{\alpha}}-\tilde{\mathbf{u}}\right)}{\tilde{\theta}}\Gamma_{\bar{\alpha}}\left(\tilde{\mathbf{u}}\right)\right]
\end{array}\right\}\mathbf{e}_{\alpha}
\end{aligned} \tag{16}$$

$$\begin{aligned}
\Delta\mathbf{CM}_{\alpha}&=\left[\frac{\tilde{f}_{\alpha}\left(\tilde{\mathbf{r}},\tilde{t}\right)-\tilde{f}_{\alpha}^{eq}\left(\tilde{\mathbf{r}},\tilde{t}\right)}{\tilde{\tau}^{*}}-\frac{\tilde{f}_{\bar{\alpha}}\left(\tilde{\mathbf{r}},\tilde{t}\right)-\tilde{f}_{\bar{\alpha}}^{eq}\left(\tilde{\mathbf{r}},\tilde{t}\right)}{\tilde{\tau}^{*}}\right]\mathbf{e}_{\alpha} \\
&-\left[\frac{\tilde{\mathbf{F}}\cdot\left(\mathbf{e}_{\alpha}-\tilde{\mathbf{u}}\right)}{\tilde{\theta}}\Gamma_{\alpha}\left(\tilde{\mathbf{u}}\right)-\frac{\tilde{\mathbf{F}}\cdot\left(\mathbf{e}_{\bar{\alpha}}-\tilde{\mathbf{u}}\right)}{\tilde{\theta}}\Gamma_{\bar{\alpha}}\left(\tilde{\mathbf{u}}\right)\right]\mathbf{e}_{\alpha}
\end{aligned} \tag{17}$$

The momentum loss created by the external body force reads

$$\Delta\mathbf{CM}_{\alpha}=-\frac{\tilde{\mathbf{F}}}{\tilde{\theta}}\cdot\left[\left(\mathbf{e}_{\alpha}-\tilde{\mathbf{u}}\right)\Gamma_{\alpha}\left(\tilde{\mathbf{u}}\right)-\left(\mathbf{e}_{\bar{\alpha}}-\tilde{\mathbf{u}}\right)\Gamma_{\bar{\alpha}}\left(\tilde{\mathbf{u}}\right)\right]\mathbf{e}_{\alpha} \tag{18}$$

$$\Delta\mathbf{CM}_{\alpha}=-\frac{\tilde{\mathbf{F}}}{\tilde{\theta}}\cdot\left[\mathbf{e}_{\alpha}\Gamma_{\alpha}\left(\tilde{\mathbf{u}}\right)-\tilde{\mathbf{u}}\Gamma_{\alpha}\left(\tilde{\mathbf{u}}\right)-\mathbf{e}_{\bar{\alpha}}\Gamma_{\bar{\alpha}}\left(\tilde{\mathbf{u}}\right)+\tilde{\mathbf{u}}\Gamma_{\bar{\alpha}}\left(\tilde{\mathbf{u}}\right)\right]\mathbf{e}_{\alpha} \tag{19}$$

$$\Delta\mathbf{CM}_{\alpha}=-\frac{\tilde{\mathbf{F}}}{\tilde{\theta}}\cdot\left\{\mathbf{e}_{\alpha}\left[\Gamma_{\alpha}\left(\tilde{\mathbf{u}}\right)+\Gamma_{\bar{\alpha}}\left(\tilde{\mathbf{u}}\right)\right]-\tilde{\mathbf{u}}\left[\Gamma_{\alpha}\left(\tilde{\mathbf{u}}\right)-\Gamma_{\bar{\alpha}}\left(\tilde{\mathbf{u}}\right)\right]\right\}\mathbf{e}_{\alpha} \tag{20}$$

Since

$$\Gamma_\alpha(\tilde{\mathbf{u}}) - \Gamma_{\bar{\alpha}}(\tilde{\mathbf{u}}) = 2w_\alpha \frac{\mathbf{e}_\alpha \cdot \tilde{\mathbf{u}}}{\tilde{\theta}} = 6w_\alpha(\mathbf{e}_\alpha \cdot \mathbf{e}_1)\tilde{u}_x \tag{21}$$

$$\Gamma_\alpha(\tilde{\mathbf{u}}) + \Gamma_{\bar{\alpha}}(\tilde{\mathbf{u}}) = 2w_\alpha \left[1 + \frac{(\mathbf{e}_\alpha \cdot \tilde{\mathbf{u}})^2}{2\tilde{\theta}^2} - \frac{\tilde{\mathbf{u}}^2}{2\tilde{\theta}}\right] = w_\alpha \left\{2 + 3\tilde{u}_x^2\left[3(\mathbf{e}_\alpha \cdot \mathbf{e}_1)^2 - 1\right]\right\} \tag{22}$$

Substituting Eq. (21) and Eq. (22) into Eq. (20) and ignore the second order terms, , the following holds: $\Delta\mathbf{CM}_\alpha \approx -6w_\alpha\left(\tilde{\mathbf{F}} \cdot \mathbf{e}_\alpha\right)\mathbf{e}_\alpha$ (23)

The momentum loss in the collision step is created by applied external force.

The momentum loss during repartition is:

$$\Delta\mathbf{RM}_\alpha = \mathbf{SM}_\alpha - \mathbf{RM}_\alpha = \left[1 - \eta(1 - 2n_s)\right]\left[\tilde{f}_\alpha^c(\tilde{\mathbf{r}}, \tilde{t}^*) - \tilde{f}_{\bar{\alpha}}^c(\tilde{\mathbf{r}}, \tilde{t}^*)\right]\mathbf{e}_\alpha \tag{24}$$

Or

$$\Delta\mathbf{RM}_\alpha = \left[(1 - \eta) + 2n_s\eta\right]\mathbf{CM}_\alpha \tag{25}$$

The momentum loss in the repartition is created by interaction between fluid and solid scatters.

In BBF model, there is no reparation step; the model parameters satisfy Eq. (7), and the interaction between the fluid and solid phase is represented through inserting macroscopic resistance force $\tilde{\mathbf{F}}_R = -k\tilde{\mathbf{u}}$ into the lattice Boltzmann scheme. The momentum loss in every lattice direction all are proportional to macroscopic velocity $\tilde{\mathbf{u}}$, i.e.

$$\Delta\mathbf{CM}_\alpha \approx -6w_\alpha\left(\tilde{\mathbf{F}}_R \cdot \mathbf{e}_\alpha\right)\mathbf{e}_\alpha = 6kw_\alpha\left(\tilde{\mathbf{u}} \cdot \mathbf{e}_\alpha\right)\mathbf{e}_\alpha \tag{26}$$

If one makes a strong assumption that flow is linear or laminar across all three levels: macroscopic scale, lattice scale and subscale (or pore scale). Suppose at the macroscopic scale $\tilde{\mathbf{u}}$ is scaled to $\varpi\tilde{\mathbf{u}}$, saying for example $\varpi = 2$, then the velocity in each pore structure and everywhere will be scaled by the same factor ϖ. In this case

Eq. (26) holds exactly for each lattice and each direction. Notice that in this case, the second term of right hand side of Eq. (27) is negligible

$$\frac{D\mathbf{v}}{dt} = \frac{\partial \mathbf{v}}{\partial t} + \mathbf{v} \cdot \frac{\partial \mathbf{v}}{\partial \mathbf{r}} \tag{27}$$

Then the Navier-Stokes equation that controls the fluid flow across all the levels becomes linear, and the fluid viscosity

$$\nu = \frac{2\tilde{\tau}^* - 1}{6} \Delta t c^2 \tag{28}$$

can be eliminated from Navier-Stokes equation. This results in the macroscopic permeability to be independent of $\tilde{\tau}^*$. However, we consider this assumption being too strong, we refer to Eq. (26) as a static momentum loss formulation.

In our GLBM, the flow resistance incurred by a subscale pore structure is modelled by a partial bounced back reparation step, not in the form of a body force $\tilde{\mathbf{F}}_R$, and therefore Eq. (8) is satisfied and the momentum loss at a pair of opposite directions is determined by Eq. (25), which is proportional to the corresponding post collision momentum. We refer Eq. (25) as dynamic momentum loss formulation.

In terms of effective viscosities that are recovered from these two models, they are completely different; GLBM, in its improved form, is isotropic, whereas BBF is intrinsic anisotropic. For any given relaxation time $\tilde{\tau}^*$, the effective viscosity recovered by GLBM is finite and independent of effective permeability. However as reported in reference paper[9], the effective viscosity recovered by BBF model is correlated to effective permeability, and it approaches to infinity if effective permeability approach to 0.

2. Compare GLBM with Classical Lattice Boltzmann Model (CLBM) The main difference between GLBM and CLBM lies in that the former does not require pores to

be explicitly represented whereas the latter does. Figure 1 shows a unit lattic cell for GLBM that represents subscale porosu media on the left and a set of much smaller lattic cells for CLBM that represents pores and grains in subscale porosu media explicitly on the right. Note that each GLBM lattice cell is assumed much smaller than the model in size, but much larger than pore size whereas each lattice cell for CLBM is much smaller than the pore or grain size.

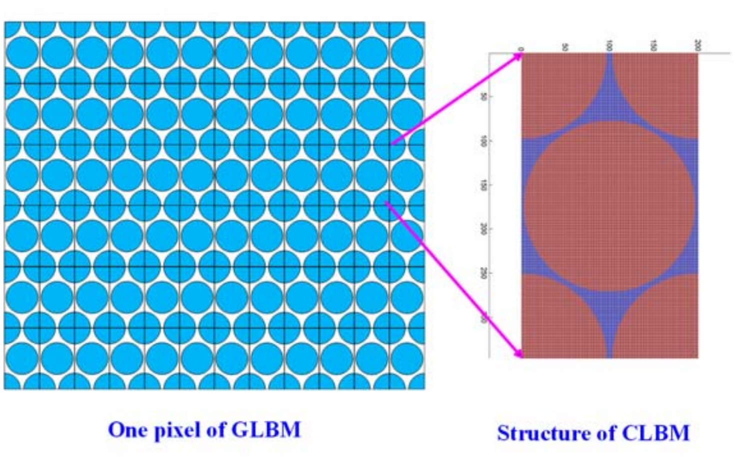

Figure 1 Illustration of an unit lattice for GLBM on the left and lattices for CLBM on the right

Reference papers[17-19] report numerical simulations of CLBM using two and multiple relaxation models. They show that if flow is laminar within pores, lattice Boltzmann simulation with exactly nonslip boundary condition will results in macroscopic permeability that is independent of $\tilde{\tau}^*$. This is consistent with the discussion in the last section. However, if nonslip boundary condition was implemented correctly, predicted macroscopic permeability will become dependent on $\tilde{\tau}^*$. It is well-known that a naive implementation of the bounce back boundary condition will not result in 0 velocity on the boundary by reference paper[13]. The reader is warned not to confuse the "bounce back" in CLBM and the partial "bounce back" in GLBM and not to attribute the viscosity-dependent effective permeability predicted by GLBM to the same effect. In GLBM, flow within a gray cell is not modelled explicitly below the

subscale but as its effective flow simulated by repartition for model parameters n_s and η without incurring actual "bounce back" operations. Finally, in all our simulations presented in this and early works, the exact nonslip boundary condition proposed in work[13] has been implemented on all solid nodes.

3. Viscosity Independent Permeability

In theory effective permeability can only become independent of $\tilde{\tau}^*$ if (1) any nonlinear effect is negligible; and (2) exact nonslip boundary condition at pore scale geometrical boundary is satisfied. If the flow at pore scale becomes turbulent in pore space, the nonlinear effect associated with it could be significant. Another possible example is fluid flow in very confined pore space where pore size is variable and comparable with free path of fluid particles. In this case, nonslip boundary condition does not hold.

It is well known that the Klinkenberg effect will significantly affect effective permeability when Knudsen number is high, where apparent permeability may be fluid viscosity dependent[20-43] like gas flow in unconventional shale and/o tight sandstone gas reservoirs.

It should be emphasized that our model parameters η, n_s are not used to make any assumptions on flow behaviors and flow and solid interaction at subscales. Therefore may be specialized to simulate any subscale flow behaviors, in principle, to meet a wide range of needs of applications including modelling shale gas and oil flow, although model parameters are phenomenal. Nevertheless, as shown below, given k_{eff} and viscosity from Eq. (15) in the main text one can show that

$$\eta = \frac{6k_{eff}}{\left(1-2n_s\right)\left[\left(2\tilde{\tau}^*-1\right)+6k_{eff}\right]} \quad (29)$$

as long as parameter η is chosen as Eq. (29), a fluid viscosity independent permeability is guaranteed.

In this case the momentum loss of GLBM reads

$$\Delta \mathbf{RM}_\alpha = \frac{\left(2\tilde{\tau}^* - 1\right)}{\left(2\tilde{\tau}^* - 1\right) + 6k_{eff}} \mathbf{CM}_\alpha \tag{30}$$

The momentum loss of BBF scheme reads

$$\Delta \mathbf{CM}_\alpha \approx \frac{6\phi \nu_f}{\kappa} w_\alpha \left(\tilde{\mathbf{u}} \cdot \mathbf{e}_\alpha\right) \mathbf{e}_\alpha \tag{31}$$

In Eq. (31), when $\kappa \to 0$, the coefficient of momentum loss approach to infinity while in Eq. (30), when $k_{eff} \to 0$ the coefficient of momentum loss approach to 1. This is another fundamental difference between BBF scheme and GLBM.

Eq. (29) indicate the model parameter η may depend not only on pore scale geometrical structure but also viscosity of fluid which fill the porous media. It should be emphasized that Eq. (29) puts model parameter η and effective permeability in an inverse order to mimic this special case, where effective permeability is independent of fluid viscosity. In most general case, effective permeability may dependent upon micro flow pattern instead of micro structure only, the ordinary order should be to calibrate two model parameters n_s and η first, then substituting model parameters into Eq. (14) and Eq. (15) of main text to get effective permeability and effective viscosity.

To summarize Appendix C, we would like make following three conclusions:

(1) To compare GLBM with BBF scheme, we think the momentum loss formulation Eq. (25) is more robust than Eq. (26).

(2) To compare GLBM with CLBM used in reference papers[17-19], CLBM used in the reference papers investigated linear problem, i.e. laminar flow with nonslip boundary

condition across all macroscopic scale, lattice scale and subscale levels; GLBM is not only suitable for linear problem but also nonlinear problem, where either/both high Knudsen number regime in pore scale level or/and turbulent flow exist in pore scale level.

(3) Three models, GLBM, BBF scheme and CLBM used in the references, are totally different from each other.
